# Decoupling the Chemical and Mechanical Strain Effect on Steering the CO_2_ Activation over CeO_2_-Based Oxides: An Experimental and DFT Approach

**DOI:** 10.1021/acsami.2c05714

**Published:** 2022-07-12

**Authors:** Kyriaki Polychronopoulou, Sara AlKhoori, Shaima AlBedwawi, Seba Alareeqi, Aseel G. S. Hussien, Michalis A. Vasiliades, Angelos M. Efstathiou, Klito C. Petallidou, Nirpendra Singh, Dalaver H. Anjum, Lourdes F. Vega, Mark A. Baker

**Affiliations:** † Department of Mechanical Engineering, Khalifa University of Science and Technology, Abu Dhabi 127788, United Arab Emirates; ‡ Center for Catalysis and Separations (CeCaS Center), Khalifa University of Science and Technology, Abu Dhabi 127788, United Arab Emirates; § Department of Chemical Engineering and Research and Innovation Center on CO2 and Hydrogen (RICH Center), Khalifa University of Science and Technology, Abu Dhabi 127788, United Arab Emirates; ∥ Department of Chemistry, Heterogeneous Catalysis Laboratory, University of Cyprus, 1 University Avenue, University Campus, 2109 Nicosia, Cyprus; ⊥ Department of Physics, Khalifa University of Science and Technology, Abu Dhabi 127788, United Arab Emirates; # The Surface Analysis Laboratory, Faculty of Engineering and Physical Sciences, University of Surrey, Guildford GU2 4DL, U.K.

**Keywords:** mechanochemistry, ball milling, surface tuning, strain engineering, CO_2_ activation, ceria, oxygen vacancies, ternary oxides, DFT, DRM, DRIFTS

## Abstract

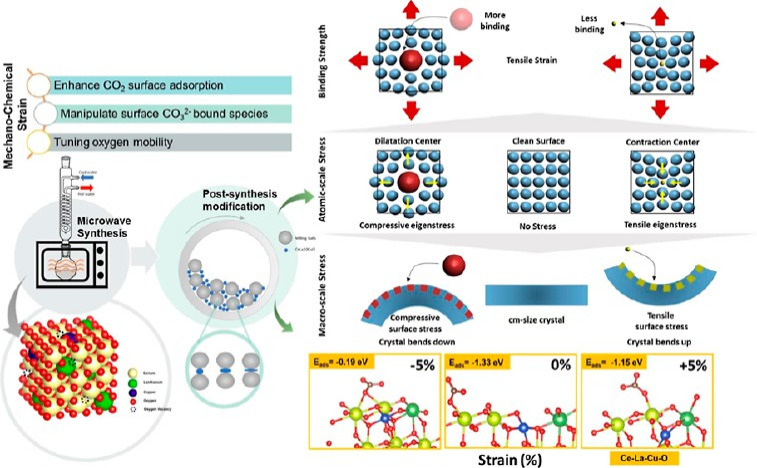

Doped ceria-based metal oxides are widely used as supports and stand-alone catalysts in reactions where CO_2_ is involved. Thus, it is important to understand how to tailor their CO_2_ adsorption behavior. In this work, steering the CO_2_ activation behavior of Ce–La–Cu–O ternary oxide surfaces through the combined effect of chemical and mechanical strain was thoroughly examined using both experimental and ab initio modeling approaches. Doping with aliovalent metal cations (La^3+^ or La^3+^/Cu^2+^) and post-synthetic ball milling were considered as the origin of the chemical and mechanical strain of CeO_2_, respectively. Experimentally, microwave-assisted reflux-prepared Ce–La–Cu–O ternary oxides were imposed into mechanical forces to tune the structure, redox ability, defects, and CO_2_ surface adsorption properties; the latter were used as key descriptors. The purpose was to decouple the combined effect of the chemical strain (ε_C_) and mechanical strain (ε_M_) on the modification of the Ce–La–Cu–O surface reactivity toward CO_2_ activation. During the ab initio calculations, the stability (energy of formation, *E*_O_v__^f^) of different configurations of oxygen vacant sites (O_v_) was assessed under biaxial tensile strain (ε > 0) and compressive strain (ε < 0), whereas the CO_2_-philicity of the surface was assessed at different levels of the imposed mechanical strain. The *E*_O_v__^f^ values were found to decrease with increasing tensile strain. The Ce–La–Cu–O(111) surface exhibited the lowest *E*_O_v__^f^ values for the single subsurface sites, implying that O_v_ may occur spontaneously upon Cu addition. The mobility of the surface and bulk oxygen anions in the lattice contributing to the O_v_ population was measured using ^16^O/^18^O transient isothermal isotopic exchange experiments; the maximum in the dynamic rate of ^16^O^18^O formation, *R*_max_(^16^O^18^O), was 13.1 and 8.5 μmol g^–1^ s^–1^ for pristine (chemically strained) and dry ball-milled (chemically and mechanically strained) oxides, respectively. The CO_2_ activation pathway (redox vs associative) was experimentally probed using in situ diffuse reflectance infrared Fourier transform spectroscopy. It was demonstrated that the mechanical strain increased up to 6 times the CO_2_ adsorption sites, though reducing their thermal stability. This result supports the mechanical actuation of the “carbonate”-bound species; the latter was in agreement with the density functional theory (DFT)-calculated C–O bond lengths and O–C–O angles. Ab initio studies shed light on the CO_2_ adsorption energy (*E*_ads_), suggesting a covalent bonding which is enhanced in the presence of doping and under tensile strain. Bader charge analysis probed the adsorbate/surface charge distribution and illustrated that CO_2_ interacts with the dual sites (acidic and basic ones) on the surface, leading to the formation of bidentate carbonate species. Density of states (DOS) studies revealed a significant *E*_g_ drop in the presence of double O_v_ and compressive strain, a finding with design implications in covalent type of interactions. To bridge this study with industrially important catalytic applications, Ni-supported catalysts were prepared using pristine and ball-milled oxides and evaluated for the dry reforming of methane reaction. Ball milling was found to induce modification of the metal–support interface and Ni catalyst reducibility, thus leading to an increase in the CH_4_ and CO_2_ conversions. This study opens new possibilities to manipulate the CO_2_ activation for a portfolio of heterogeneous reactions.

## 1 Introduction

In heterogeneous catalysis, strained surfaces can be found in both metal-supported catalysts and multi-elemental metal oxides due to a lattice constant mismatch between metal–support and host–guest interactions, respectively.^1^ Such lattice strain enhances the chemisorption properties of the catalytic surface significantly,^2^ either for the adsorbate or for the intermediate species formed under reaction conditions. As a strain normally manipulates the surface ability to form bonds, there is a great possibility in using a strain as a catalyst reactivity modifier/descriptor.^2^

Ball milling is a mature mechanical activation technique that can lead to the fabrication of nanocatalysts with anomalous properties compared to their bulk counterparts. Through mechanical activation, solid-state chemical reactions can be initiated/accelerated while causing various transformations and reactions, such as grain boundary disordering, amorphization, defect generation/migration, polymorphic transformations, ion coordination sphere change, and reduction in particle size.^3^ The main function that takes place during ball milling is that the material’s potential or its stored mechanical energy is enhanced in the presence of ball milling forces.^4^ This is translated into defects (point, line, and volume ones), surface and interface formation, strain and structural disorder, and changes in electronic states. Some of the parameters in the ball milling process that can alter the impact of mechanical activation in terms of the final catalyst properties (e.g., surface area, active metal dispersion, and binding strength of the intermediates) are the ball size, number of balls used, ball-to-powder ratio, rotational speed, milling time, and milling atmosphere.^5,6^ It is well known that the ceria morphology and thus its properties can be tuned through synthesis. A variety of synthetic methods have been used spanning from mild hydrothermal to form ceria nanotubes^7^ and nanorods with different types and distributions of oxygen vacancies^8^ to template-free microwave-assisted hydrothermal synthesis and urea homogeneous precipitation; the latter ceria materials were used for the preparation of Pt/CeO_2_ catalysts for the WGS reaction,^9^ demonstrating the synthesis impact on the electronic/catalytic properties.

In the context of sustainable development, there is an increasing interest in the utilization of the captured CO_2_ from a flue gas to form valuable chemicals and products. In this sense, two of the catalytic reactions of most interest are dry reforming of methane (DRM) and CO_2_ hydrogenation. The DRM is very relevant in this context as it simultaneously tackles the abatement of two greenhouse gases (CH_4_ and CO_2_) while leading to the production of hydrogen (energy carrier). The CO_2_ hydrogenation leads to the valorization of CO_2_ into different value-added products. However, it is well known that the inert property and stability of the CO_2_ molecule pose a challenge in both aspects: thermodynamics and kinetics.^10^ Upon CO_2_ adsorption on metal and metal oxide surfaces, the molecule’s stability is reduced. The adsorption configuration is highly dependent on the surface Lewis acidity/basicity. In an ideal scenario, a bent configuration is favored by charge transfer (CT) from the surface (Lewis base, electron reservoir) to the CO_2_ molecule. There are reports showing that both CO_2_ linear and bent configurations on the ceria surface are favored.^11,12^ It has also been reported that reduced ceria surfaces are favorable for carbon dioxide reduction reactions due to the involvement of polarons (Ce^3+^–O_v_ pairs) in the CO_2_ molecular adsorption at oxide surfaces, thus enhancing CT that is the initiator of such chemical reactions. It is therefore important to obtain insights into the surface chemical reactivity and its CO_2_-philicity, as well as how this can be steered. Acidic surfaces favor the formation of linear-type CO_2_ adsorption configuration, whereas basic surfaces favor the CO_2_^–^ formation (bent species and reactive ones).^13^

From the *aspect of catalyst design for CO*_*2*_*activation*, some criteria need to be considered, such as (a) the use of metal oxide supports with high basicity (e.g., MgO and La_2_O_3_) can enhance the dissociative adsorption of CO_2_, which inhibits carbon formation by creating a higher number of oxygen atoms around the catalyst-active metal surface.^14^ The improvement of CO_2_ dissociation on catalysts by increasing the surface basicity can similarly deactivate the catalyst.^14^ It has been verified lately that excessive surface basicity/acidity can cause deactivation due to carbon formation in reactions such as DRM,^15^ where the importance of moderate acidity and basicity along with the homogeneous dispersion that ideally defines catalytic conversion in the DRM reaction and the long-term stability of supported metal catalysts was demonstrated. (b) Oxygen vacancies (O_v_) can be classified as intrinsic, naturally existing in the material (e.g., due to the presence of Ce^4+^/Ce^3+^), or extrinsic, induced by the doping of CeO_2_ lattice with aliovalent metal cations for better ionic conductivity.^16,17^ Among the doped ceria catalysts, the Ce–Cu–O system exhibits the (i) high redox properties and the ability to switch between Ce^3+^/Ce^4+^ and Cu^2+^/Cu^1+^, (ii) increasing population of oxygen vacant sites (O_v_), and (iii) increase of labile surface and bulk oxygen species compared to the single-phase oxide.^18^ Therefore, it can be stated that O_v_ has a critical role in the CO_2_ dissociation on ceria surfaces and thus is expected to have a leading role in catalytic reactions where CO_2_ is a reactant or a co-reactant.^19^ It is also well established that strain can modify the defect (e.g., O_v_) structure and electronic properties.^20^ While the tensile strain leads to surface vacancies having polarons as the next neighbor (NN), the compressive strain is associated with subsurface vacancies having polarons as NN, whereas dimer vacancies are also favored over compressive strain.

Mechanical forces are used either to prepare or to actuate ceria-based catalysts and devices. For instance, in the preparation of ceria-based catalysts, the latter used for environmental and energy applications has been reported in many review articles using mechanochemical methods.^21^ Demonstration of the superiority of Pd/CeO_2_ ball-milled catalysts for the methane oxidation reaction at low temperature, above 95% conversion, over the traditional wet impregnation catalysts has been discussed by Trovarelli’s research group even at conditions which are challenging for the particular reaction.^22,23^ Furthermore, there is extensive literature on the impact of different types of external stimuli on thin films of ceria; for example, the electrochemomechanical effect has been reported by Lubomirsky and his colleagues^24,25^ to produce stress that can potentially deteriorate them.^22,23^ Furthermore, strain engineering has been reported to increase the CT and electronic conductivity.^26^ However, the effect of applying compressive or tensile stress on doped ternary ceria (111) surfaces has not being comprehensively investigated in the literature yet in the context of steering of its catalytic chemistry and adsorption behavior. The oxide surfaces (Ce–La–Cu–O) studied in the present work exhibit a versatile catalytic functionality that spans from reforming^27^ to oxidation chemistry,^18^ given their noble-metal-free nature is worthy of more attention. Finding a way to engineer the vacancy population and structure, and, hence, the CO_2_–surface interaction, can be an additional tool toward catalyst design for CO_2_ activation, thus contributing to a more rational design of catalysts for reactions such as DRM and CO_2_ hydrogenation.

In the present work, microwave-prepared Ce–La–Cu–O ternary metal oxides were subjected into mechanochemical activation under dry and wet ball milling targeting their intrinsic property modification, namely, structure, crystallite size, specific surface area, redox properties, CO_2_ activation energy, and pathway. The main emphasis was given on exploring how chemical strain, ε_C_ (doping effect), and mechanical strain, ε_M_ (compressive or tensile, originated by ball milling), can impact the oxygen vacancy formation and population [O_v_] and their chemical reactivity toward CO_2_ activation. Experimental and density functional theory (DFT) studies were performed in an effort to deconvolute the ε_C_ and ε_M_ impact. A versatile toolbox including X-ray powder diffraction, Raman spectroscopy, transmission electron microscopy (TEM), X-ray photoelectron spectroscopy (XPS), ^18^O_2_-transient isothermal isotopic exchange (TIIE), in situ CO_2_ diffuse reflectance infrared Fourier transform spectroscopy (DRIFT) spectroscopy, H_2_-temperature-programmed reduction (H_2_-TPR), and CO_2_-temperature-programmed desorption (CO_2_-TPD), along with intensive and systematic ab initio calculations was employed to fully characterize and analyze the performance of the solids. The effect of supports during ball milling on the performance of Ce–La–Cu–O supported Ni catalysts toward the DRM reaction was evaluated to provide evidence for the value that mechanochemistry can bring into catalysis.

## 2 Materials and Methods

### 2.1 Preparation of Ternary Metal Oxides

A microwave-accelerated reaction system (MARS-6) was used to synthesize the catalysts through microwave-assisted reflux synthesis. The microwave system had a power output of 0–1800 W ±5% (IEC 705 Method-1988).^18^ Materials were prepared using precursor salts Ce(NO_3_)_3_·6H_2_O (Aldrich 99.95%), La(NO_3_)_3_·6H_2_O (Aldrich 99.95%), Sm(NO_3_)_3_·6H_2_O (Aldrich 99.95%), and Cu(NO_3_)_2_·3H_2_O (Aldrich 99.95%) and dissolved in distilled water. The prepared mixed metal oxides had 10 at. % Cu content, while the Ce/M (M: La and Sm) ratio was maintained at unity. The total molar ratio was maintained at 0.03 mol in all cases. The complexing agent ethylene glycol (EG) was added to the solution with a ratio of EG to water retained at 2; that is, for each 100 mL of EG, 50 mL of distilled water was added.

The final solution was added to an open 1000 mL round-bottom flask in an open vessel mode, equipped with an oval magnetic stirring bar (length 20 mm and diameter 6 mm) made with polytetrafluoroethylene coating. A reflux system was attached to the open round flask of the MW system, allowing water to pass through the reflux for condensation. Parameters, such as stirring, reaction temperature, MW power, and heating/cooling ramp, were all adopted from a previous study.^1^ The temperature of the reaction was monitored by built-in fiber optic probes. The solution was heated in the microwave reactor at two stages: (i) 130 °C for 2 h and (ii) 170 °C for 1 h at 800 W of magnetron power. Following microwave heating, all synthesized materials were calcined at 500 °C for 6 h under ambient conditions to form the mixed metal oxide catalyst. In what follows, the wet ball-milled and dry ball-milled samples were coded as wet ball milling (WBM) and dry ball milling (DBM), respectively.

### 2.2 Ternary Oxides Post-Synthesis Mechanochemical Treatment (Ball Milling)

The synthesized materials were ball-milled using a planetary ball mill (Planetary Mill PULVERISETTE 5, Fritsch) under ambient conditions. A relevant amount of catalysts was mixed with distilled water, maintaining a weight ratio of 12:1. The same catalysts were ball-milled in a dry medium to track any structural changes. The catalysts were milled at 250 rpm at different milling times in a zirconia jar with a mass ratio of balls to solid powder equal to 100. The balls were made of zirconia and had a mass of around ∼3 g each. The ball-milled catalysts under wet condition were dried at 150 °C for 3 h. Wet milling took place for 4 and 10 h, whereas DBM took place from 0 to 10 h at an interval of 2 h.

### 2.3 Supported Ni Catalyst Preparation

Pristine Ce–La–Cu–O oxides along with the ones following DBM (Ce–La–Cu–O) and WBM (Ce–La–Cu–O) were used as supports for the deposition of Ni metal phase (supported Ni catalysts) following the wet impregnation method (5 wt % loading) as described in our previous work.^27^

### 2.4 Characterization of Ternary Metal Oxides

Powder X-ray diffraction (XRD), Raman spectroscopy, electron paramagnetic resonance (EPR), scanning electron microscopy, TEM, H_2_-TPR, CO_2_-TPD, and XPS were employed to study the structural, textural, redox, and CO_2_ adsorption properties in the pristine (following calcination) and ball-milled (wet and dry) ternary metal oxides. The instrumentation and the experimental protocol are provided in the Supporting Information (see Section S1.3).

### 2.5 ^18^O/^16^O TIIE

The surface and bulk oxygen mobility/diffusion in the mixed metal oxides, particularly Ce–La–10Cu–O, was investigated using ^18^O_2_-TIIE experiments. The step-gas isotopic switch, 2 mol % ^16^O_2_/2 mol % Kr/Ar/He (*T*, 30 min) → 2 mol % ^18^O_2_/Ar/He (*T*, *t*), was conducted over a 20 mg sample with a total volume flow rate of 50 N mL/min. The sample was first pre-calcined under 20 vol % ^16^O_2_/He gas flow at 800 °C for 2 h, then Ar was passed over the sample for 10 min, and the temperature was then decreased to 350 °C in Ar gas flow, followed by the TIIE step-gas switch. During the TIIE experiment, the dynamic evolution of the rates of oxygen exchange between the gas-phase oxygen, lattice oxygen, and oxygen vacant sites was recorded.

The transient response curves of the three oxygen isotopic gases and of Kr tracer (inert gas) were continuously recorded by an on-line mass spectrometer (Balzers, Omnistar, 1–300 amu) for the mass numbers (*m*/*z*) 32, 34, 36, and 84, corresponding to the ^16^O_2_, ^16^O^18^O, ^18^O_2_, and Kr signals, respectively. Following the surface ^16^O/^18^O exchange, the lattice oxygen (^16^O) in the solid diffuses from the bulk to the surface, and the ^18^O lattice oxygen diffuses from the surface to the bulk.

For the continuous stirred-tank reactor (CSTR) behavior considered in this study,^22^ the transient rates (μmol/g s) of ^16^O^18^O(g) (eq 1) and ^16^O_2_(g) (eq 2) formation were calculated
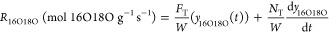
1
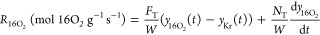
2

In eqs 1 and 2, *F*_T_ is the total molar flow rate (mol/s) of the feed gas stream, *y*_^16^O^18^O_, *y*_^16^O_2__, and *y*_Kr_ are the mole fractions of ^16^O^18^O, ^16^O_2_, and Kr at the outlet of the CSTR microreactor, respectively, *N*_T_ is the total number of moles in the CSTR reactor, and *W* is the sample’s mass (20 mg).^22^ The total rate of ^16^O exchange with ^18^O and *R*(^16^O) (mol ^16^O g^–1^ s^–1^) and the transient evolution of the amount of ^16^O and *N*(^16^O) (mol ^16^O g^–1^) were estimated based on ^16^O-material balance eqs 3 and 4
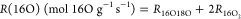
3
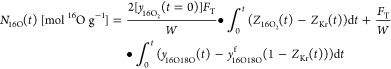
4where *Z*_*i*_ is the dimensionless response of gaseous species *i*, *y*_*i*_ is the mole fraction of gaseous species *i*, *y*(^16^O_2_) (*t* = 0) is the mole fraction before the step-gas switch (2 vol %), and *y*^f^(^16^O^18^O) is the mole fraction of ^16^O^18^O(g) present in the 2 mol % ^18^O_2_/Ar/He feed gas mixture used.^28^

The *a*_g_^18^(*t*) descriptor function was used to investigate both the surface ^16^O/^18^O exchange and the oxygen diffusion in the bulk via eqs 5 and 6
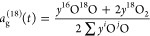
5
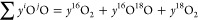
6

### 2.6 In Situ Diffuse Reflectance Infrared Fourier Transform Spectroscopy

Diffuse reflectance infrared Fourier transform spectroscopy (DRIFTS) studies were performed using a PerkinElmer Frontier Fourier transform infrared spectroscopy spectrometer (256 scans per spectrum, resolution 4 cm^–1^, and scan speed 2 cm/s) equipped with a high-temperature/high-pressure-controllable DRIFTS cell (Harrick, Praying Mantis) to investigate (i) the intermediate-adsorbed species formed under CO_2_/Ar gas treatment and (ii) the stability of the adsorbed intermediate species under Ar inert gas. DRIFTS spectra were recorded in 5 vol % CO_2_/Ar gas flow at 350 °C and after 30 min in Ar gas flow at 350 °C for various times in Ar flow. The catalyst sample (∼80 mg) in a very fine powder form was placed firmly into the ceramic cup of the DRIFTS cell and the temperature was increased to 500 °C in Ar gas flow and kept for 30 min. Then, the sample was cooled to 350 °C in Ar gas flow, and the spectrum of the solid was recorded at 350 °C. The latter spectrum was subtracted from the spectrum of the solid recorded in the CO_2_/Ar gas mixture or Ar gas flow at 350 °C. Deconvolution and curve fitting procedures of DRIFTS spectra were performed considering the Gaussian peaks. DRIFTS spectra when necessary were smoothed to remove the high-frequency noise and further analyzed using the software Spectrum10 for Windows.

### 2.7 Ab Initio DFT Calculations

#### 2.7.1 Energy of Oxygen Vacancy Formation (*E*_O_v__^f^)

The first-principles spin-polarized calculations, based on the DFT,^29,30^ were performed after employing the Vienna Ab Initio Simulation Package (VASP).^31−33^ The electronic exchange was used under the generalized gradient approximation (GGA) of Perdew–Burke–Ernzerhof.^34^ To include the strong correlation effects of 4f electrons, GGA with the Hubbard *U* correction method^35,36^ was used with a value of *U* = 5.0 eV.^37−39^ The DFT + *U* method accounts for the O 2p states and has been previously followed in DFT studies as a common practice to accurately describe the oxygen electronic behavior rising from the doping effect.^40,41^

The plane-wave cut-off energy was set to 400 eV^42^ in all the calculations, and the projector-augmented wave pseudopotentials^43^ were used to describe the core electrons. The (111) slab was built from the optimized CeO_2_ unit cell with a lattice parameter equal to 5.46 Å, in agreement with the literature.^44^ The slab consisted of nine (9) layers terminating with oxygen with a vacuum of 15 Å in the vertical direction to avoid interaction between neighboring images due to periodic boundary conditions. A γ-centered *k*-point of 3 × 3 × 1 in the full Brillouin zone, with an energy convergence criterion of 10^–6^ eV, was employed. A (2 × 2) CeO_2_(111) surface was constructed to create a vacancy in pristine CeO_2_ and the doped CeO_2_ systems (Ce–La–O and Ce–La–Cu–O). The oxygen vacancies were created at different locations by removing one or two of the denoted oxygen atoms at a time. The three bottom layers were fixed in their bulk positions to avoid the change in the crystal structure under compressive (−5 to −1%) or tensile (1 to 5%) strain.

#### 2.7.2 Two-Dimensional Planar Lattice Strain

Biaxial strain was isotropically applied by stretching or shrinking the simulation cell in the *x*, *y* directions while relaxing the cell along the *z* direction. The structure optimizations were performed until the energy-convergence criterion was satisfied, ca. 1 × 10^–6^ eV. The atoms in the top six layers were allowed to relax in all the three directions (*x*, *y*, and *z*). A biaxial strain of −5 to 5% was applied to investigate the effect of biaxial strain on the oxygen vacancy formation energy (**E**_**O**_**v**__^**f**^) in the case of doped/co-doped ceria surface layer of the slab. The **E**_**O**_v__^**f**^ value was calculated using eq 7
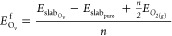
7where  is the total energy of the relaxed pure/doped surface with oxygen vacancies after applying the biaxial strain or without the biaxial strain,  is the total energy of the pure/doped slab with the same applied strain of  but without the oxygen vacancy,  is the energy of , and *n* is the total number of oxygen vacancies created in the system. Negative (<0) and positive (>0) values of  correspond, respectively, to spontaneous and nonspontaneous O_v_ formation.

#### 2.7.3 CO_2_ Adsorption Energy

In order to understand how the doping (*chemical strain*) and the imposed biaxial *mechanical strain* affect the CO_2_ adsorption, pure CeO_2_ and doped/co-doped ceria (Ce–La–O and Ce–La–Cu–O) surfaces were used to calculate the adsorption energy (*E*_ads_) of CO_2_. Two scenarios were considered: (a) in the presence of oxygen vacancies (O_v_) at zero applied biaxial mechanical strain and (b) in the absence of oxygen vacancies (O_v_) but subjecting the slabs to biaxial strain. A linear configuration of a single CO_2_ molecule was adopted on the constructed slabs, and eq 8 was implemented to extract the *E*_ads_ values.
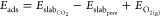
8 is the total energy of the slab with the adsorbed CO_2_ molecule,  is the total energy of the slab without the adsorbate (CO_2_), and  is the total energy of the isolated CO_2_ molecule. As linear CO_2_ is not activated, the hypothesis was to study how the chemical and mechanical strain enhances the CT, which will be evident by the bending and activation of CO_2_ after relaxing the full configuration. A negative value of the adsorption energy means that the molecule is exothermically adsorbed (*E*_ads_ < 0), while a positive value indicates that the molecule is endothermically adsorbed (*E*_ads_ > 0); the more stable adsorption state is implied by the more negative value. The coordinates of the unit cell were obtained from materials project,^45^ the slabs were viewed through VESTA,^46^ and the CeO_2_(111) surface was cleaved using VESTA. All the above calculations were run multiple times to estimate the possible errors introduced from the VASP calculations; the error was <1%.

## 3 Results and Discussion

### 3.1 Microstructural Studies

**Figure 1:**
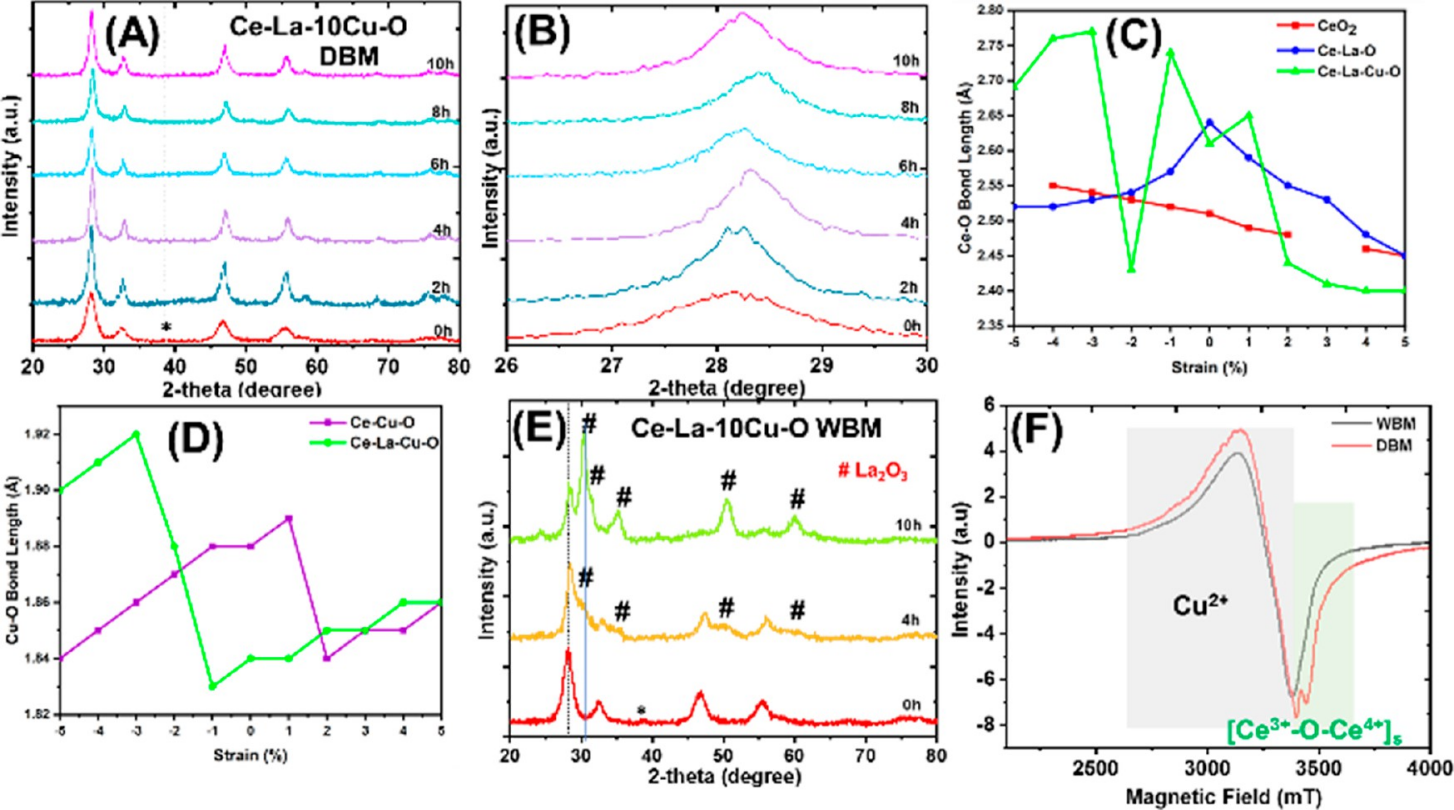
(A) XRD patterns of Ce–La–10Cu–O ternary oxides following DBM treatment for 0, 2, 4, 6, and 10 h; (*) denotes the CuO(111) phase impurity; (B) zoom in of the XRD patterns of (A) in the 26–30° 2θ region corresponding to the (111) diffraction plane; (C) Ce–O bond length (Å) in CeO_2_, Ce–La–O, and Ce–La–10Cu–O oxides calculated through the ab initio studies; (D) Cu–O bond length (Å) in Ce–Cu–O and Ce–La–10Cu–O oxides calculated through the ab initio studies; (E) XRD patterns of the Ce–La–10Cu–O ternary oxides following WBM treatment for 0, 4, and 10 h; (*) denotes the CuO(111) phase impurity, and (#) denotes the La_2_O_3_ phase impurity; and (F) EPR spectra of the Ce–La–10Cu–O ternary oxide following DBM and WBM treatment for 4 h.

Figure 1A compares the powder XRD patterns of the Ce–La–10Cu–O mixed metal oxide composition before (0 h) and after DBM for different milling times (ca. 2–10 h) with an interval of 2 h. A detailed presentation of the (111) diffraction peak position is presented in Figure 1B. For the reference oxides (CeO_2_ and Ce–La–O), the corresponding XRD patterns are provided in Figure S1. For the case of Ce–La–O due to the La content (50%) and anticipated heavy ceria doping, the La_2_Ce_2_O_7_ pyrochlore structure of fluorite type can be formed. Even though the present XRD pattern does not allow for its determination, more discussion is provided in the Raman section. The main ceria fluorite cubic lattice reflections of (111), (200), (220), (311), (222), and (400) at diffraction angles of 28.5, 33, 47.5, 56.3, 59.1, and 69.3° can be noticed in all the cases of Ce–La–Cu–O DBM oxide (Figure 1A,B). It is also important that for the pristine Ce–La–10Cu–O (absence of ball milling), a small peak corresponding to the presence of CuO can be observed at 38° 2θ (noted with *), which is vanishing with ball milling (Figure 1A). Figure 1C,D presents the Ce–O and Cu–O bond lengths, respectively, as those have been calculated through modeling approach; their trends are discussed shortly. Figure 1E presents the powder XRD patterns of the Ce–La–10Cu–O ternary oxide following 4 h of WBM. The ball milling process under wet atmosphere (water slurry) resulted in a successive appearance of a new crystalline phase, as indicated by the peaks at 26, 29, 31, 45, and 60 2θ, corresponding to the (100), (002), (101), (110), (112), and (201) diffraction planes of La_2_O_3_.

From the detailed analysis of the (111) reflection shown in Figure 1B, a peak shift to higher 2θ angles can be observed for milling times up to 4 h due to a compressive strain developed along the Ce–O bond axis in the lattice and possible move of atoms to interstitial sites under the mechanochemical forces.^47^ The compression of the Ce–O bonds was also supported through the ab initio calculations (see Figure 1C). It has to be mentioned that an opposite trend was noticed along the Cu–O bond (elongation, see Figure 1D). As the ball milling duration was increased beyond 4 h, either emerging of new peaks corresponding to impurity phases (La(OH)_3_ and La_2_O_3_) is noticed (WBM) or vanishing of the CuO heterophase peak (DBM) (Figure 1B,E). An increase of milling time is expected to lead to an increased misorientation between neighboring grains causing the formation of high-angle boundaries. As the high-energy milling continues, the crystallite size reaches a critical value. Further energy input to the critical size crystals leads to additional crystal deformation, energy accumulation at the surface, followed by some extent of amorphization.^2^

Figure 1F presents the EPR spectra obtained over the DBM and WBM samples. The sharp signal at ∼3400 mT is usually associated with [Ce^3+^–O^–^–Ce^4+^] (*S* = 1/2)^48,49^ species on the surface. Since the presence of Ce^3+^ is usually linked to the presence of Ov, it can be stated that Ce^3+^ can also be linked with high surface activity. The signal at 2600–3300 mT is linked to Cu^2+^ (*S* = 1/2 and *I* = 3/2) ions. The shape of the EPR line shows the Cu–Cu dipolar interactions, that is, in a distance of 10 ± 3 Å from each other.^50^

Based on the powder XRD studies, two types of crystallite size were calculated, and the obtained results are listed in Table 1. Using the Scherrer formula (broadening of the XRD peak originates from the crystallite size only), the crystallite size, *D*_S_ (nm), was calculated after neglecting the strain. In addition, accepting strain-induced broadening of the XRD peaks, due to crystal imperfections and distortion, the Williamson–Hall (W–H) method was used.^51^ In the W–H plot method, *D*_W–H_ (nm) does not depend on 1/cos(θ), but it does change with tan(θ). This difference is important when small crystalline size and strain co-exist. The equation used for the W–H calculations represents the uniform deformation model (UDM), where a uniform strain across crystallographic orientations is assumed. It is observed that the UDM crystallite size systematically decreases as the milling time increases up to 8 h, and then saturation occurs that leads to an increase of the size after 10 h of milling. This trend can also be related with the equilibrium state of ball milling, where the particle size reduction is escorted by particle size enlargement. In such a case, smaller particles are agglomerated. This is quite consistent with the high-resolution TEM (HRTEM) analysis and the observed refinement of the solid solution nanodomain observed following DBM, as it will be discussed later.

**Table 1:** Textural Properties of the CeO_2_, Ce–La–O, and Ce–La–10Cu–O Solids Studied^a^

solid	BET (m^2^/g)	*D*_S_ (nm)	*D*_W–H_ (nm)	pore size (nm)	lattice parameter (Å)	lattice strain, ε
CeO_2_	36.6	7.8		8.7–10.1	5.459	0.0012
Ce–La–O	5.8	4.6		43–47.4	5.500	0.0038
Ce–La–10Cu–O-pristine	4.9	4.5		18.8–19.3	5.488	–0.0030
Ce–La–10Cu–O-WBM-4 h	14.5	4.5		11.4–12.2	5.440	
Ce–La–10Cu–O-DBM-2 h		10.5	15.93		5.471	0.0047
Ce–La–10Cu–O-DBM-4 h	8.1	9.7	12.77	13.8–14.4	5.459	0.0025
Ce–La–10Cu–O-DBM-6 h		11.4	13.58		5.470	0.0026
Ce–La–10Cu–O-DBM-8 h		7.9	9.82		5.444	0.0019
Ce–La–10Cu–O-DBM-10 h	3.5	12.8	15.48	18.9–20.3	5.474	0.0044

a *D*_S_ was calculated based on the Scherrer formula. *D*_W–H_ was calculated based on the W–H plots. Lattice strain was calculated based on the W–H plots.

It is also noticed that the Ce–La–10Cu–O lattice parameter is generally decreased in the case of WBM (5.488–5.440 Å) and DBM (5.488–5.454 Å), more likely indicating a greater extent of substitution of Ce^4+^ by La^3+^ and Cu^2+^ guest cations under the severe milling conditions. The observed change in the lattice parameter can be justified with a preferred uptake in the crystal lattice of Cu (decrease) or La (increase) with a concomitant change in the lattice parameter, depending on the applied mechanical forces. The energy input introduced through ball milling enhances the solubility of the two dopant elements (La and Cu) in the host ceria matrix,^3^ but we need to keep in mind that the diffusion is highly dependent on the atomic size of the diffusing species, as well as of host matrix atoms.^2^

The W–H method was also used to study the lattice strain (ε) of the catalysts. The W–H plots are provided in Figure S2. It is observed that after doping ceria with La and La/Cu induces a different lattice strain, namely, from 0.0012 (pure ceria) to 0.0038 (tensile strain in Ce–La–O) and −0.0030 (compressive strain in Ce–La–Cu–O). Mechanical ball milling changed the overall lattice strain in the Ce–La–Cu–O oxide from compressive to tensile. The calculated W–H strain trends are in agreement with the sizes of the dopant elements (Cu^2+^, La^3+^ vs Ce^4+^, Ce^3+^). In addition, after increasing the time of DBM (0–10 h), this leads to a profile that includes an initial increase of the lattice strain (DBM 2 h), followed by decrease (4–8 h, DBM) and a final significant increase after 10 h of DBM. This trend/profile can be due to the high pressure and high temperature spots (“hot spots”) developed under ball milling, thus causing some relaxation in the lattice. The hot spots can also drive the process of phase transformations.^52^ It is also known that during the milling process, a drop of milling effectiveness can occur. According to the ball milling fundamentals, during the milling process, three stages can take place, namely, the *Rittinger stage* (a), where particle interaction is minimal; the *aggregation stage* (b), where particle interaction leads to aggregation, and thus, the surface area produced does not correspond to the energy input; and the *agglomeration stage* (c), where the dispersion is reduced and then vanished.^53^

**Figure 2:**
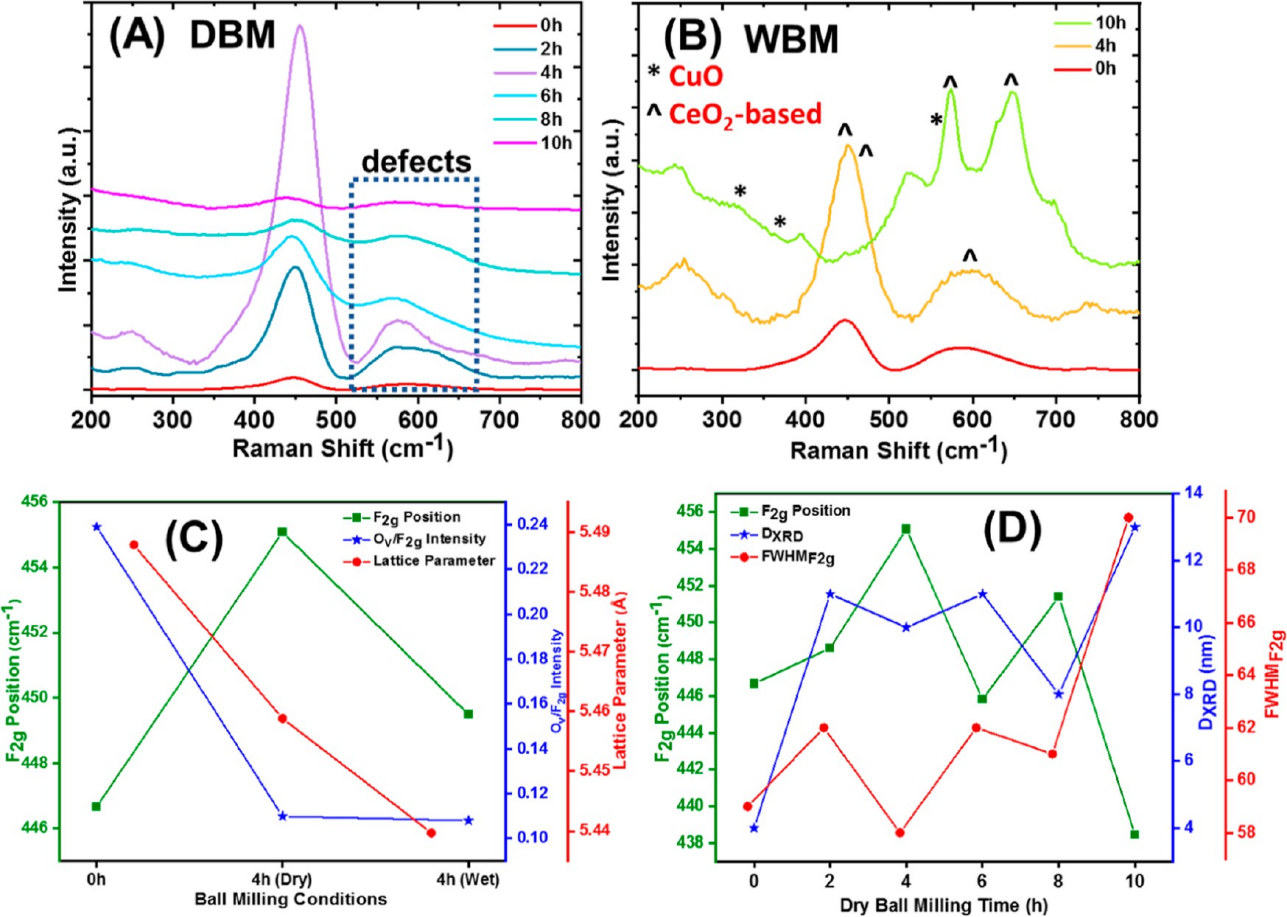
(A) Raman spectra obtained over the Ce–La–10Cu–O ternary oxides following DBM treatment for *t* = 0, 2, 4, 6, and 10 h; (B) Raman spectra of the Ce–La–10Cu–O ternary oxides following WBM treatment for *t* = 0, 4, and 10 h (*, ^ denote the CuO- and CeO_2_-based phases); (C) effect of ball milling conditions/atmosphere on the F_2g_ position (Raman band), O_v_/F_2g_ ratio (Raman bands ratio), and lattice parameter, Å (based on the XRD); and (D) effect of dry milling time on the F_2g_ position, O_v_/F_2g_ ratio, and lattice parameter (Å).

Raman spectroscopy studies were conducted to investigate the strain, phenomena such as phonon confinement and stoichiometry defects, based on the shifting and broadening of the spectral lines. In addition, they were conducted to probe the O sublattice (CeO_8_) distortions due to doping and mechanochemical treatment. The Raman spectra for Ce–La–10Cu–O, before (*t* = 0 h) and after DBM for different milling durations (*t* = 0–10 h), are shown in Figure 2A. The Raman spectra for the reference oxides (CeO_2_ and Ce–La–O) are provided in Figure S3. The F_2g_ peak centered at 469 cm^–1^ (Figure 2A) is the characteristic band for the cubic fluorite lattice of ceria and reflects the vibrational mode of oxygen surrounding Ce^4+^ ions in the CeO_8_ coordination environment.^54^ It is well documented that doping ceria, in the present study with La^3+^ and Cu^2+^ cations, results in an intensity attenuation and red shift of the F_2g_ peak to lower values, namely, from 469 to 452 and 447 cm^–1^ for the case of Ce–La–O and Ce–La–10Cu–O, respectively (Figure S3A). This shift can be justified by the increased lattice strain due to the incorporation of the dopants, for example, due to La^3+^ addition (tensile), 2M_2_O_3_ + 3Ce_Ce_^×^ + O_O_^×^ → 4M_Ce_^′^ + 2V_O_ + 3CeO_2_, and/or Cu^2+^ addition (compressive), , that generated defects, such as oxygen vacancies and lattice distortion/expansion.^55^ It has also been reported that in La-doped systems, the F_2g_ shift is about 6 cm^–1^, whereas in heavily doped systems, the shift can reach up to 15 cm^–1^; such a case is found in the La_2_Ce_2_O_7_ pyrochlore type of structure.^56,57^

Doped ceria with metal cations can improve the oxygen mobility of the catalyst by lowering the barrier for oxygen migration and minimizing the activation energy for ceria reduction (Ce^4+^ → Ce^3+^),^58^ as it will be discussed later (Section 3.7). Furthermore, recent density of states (DoS) studies from our group^59^ showed that doping ceria leads to the formation of energy states, which host the electrons left behind in the oxygen vacant site, thus facilitating the formation of O_v_. For each Cu^2+^ ion introduced into the lattice, one oxygen vacancy is expected to be formed,^55^ which is anticipated to cause tensile strain that leads to the elongation of M–O bond and therefore an increase in the number/density of mobile oxygen vacancies.^60,61^ This has been demonstrated to apply in the case of Ce–La–Cu–O solid by elongated Cu–O and compressed Ce–O bonds in the tensile strain region (Figure 1C,D) according to the present DFT calculations (Table S1).

A closer look into Figure 2A (DBM) and Figure 2B (WBM) shows intense changes in the F_2g_ band shape and size following the mechanochemical treatment. For both DBM and WBM, there is an optimum in the duration of the mechanochemical treatment, ca. 4 h, where either the F_2g_ peak intensity is maximum (DBM) or there is no formation of phase impurity (WBM), in agreement with the XRD studies. Further increase of the mechanochemical treatment time either induces changes to the structure (WBM) or decreases the F_2g_ band intensity (DBM). It can be noticed that 10 h treatment leads to almost vanishing of the F_2g_ peak under DBM conditions, whereas under WBM, the peaks that correspond to the appearance of the hexagonal La(OH)_3_ phase are noticed; the latter is most likely due to the dehydroxylation of the oxide surface and a subsequent reaction of the OH entities with La^3+^ (La^3+^ + 3 OH^–^ → La(OH)_3_) under milling forces.^53^

The bands in the region below <400 cm^–1^ (e.g., 250 cm^–1^) are usually assigned to oxygen defects in ceria due to Brillouin zone scattering.^61^ The increase of the intensity of those bands up to a milling time of 4 h agrees with the above observations in agreement with the XRD interpretations. These bands are more evident under DBM, suggesting a larger amount of oxygen defects (O_v_) present in DBM oxides compared to the WBM ones. A detailed analysis of the F_2g_ band is presented in Figure 2C, showing the effect of lattice strain and phonon confinement on the band shape and oxygen stoichiometry.^62^ It is worth noting that Raman band broadening implies the reduction in the size of the nanocrystallites, whereas a shift in Raman frequency (Δ cm^–1^) is associated with tensile or compressive strain when a red/blue shift, respectively, occurs. The combination of the phonon confinement and strain on the Ce–La–Cu–O nanocrystallites can make the interpretation hard. In this study, Ce–La–Cu–O oxides in the 4–12 nm size range were employed, making the phonon confinement effect significant in all the cases presented herein. However, the broadening and shift of Raman peaks are predominantly due to the strain and O_v_ defects originated from the milling treatment.

Figure 2C presents a drop in the lattice parameter in the presence of mechanical strain (ball milling, DBM/WBM), and this is in agreement with the Ce–O bond shrinkage estimated under tensile strain conditions (DBM was found to induce tensile strain, Table 1). Only the cases of 4 h milling are presented as this was found to preserve to the highest degree of the fluorite structure. In addition, in Figure 2D, a general increase of FWHM_F_2g__ was observed with the milling time ranging from 0 to 10 h. This broadening of Raman peak full width at half-maximum (FWHM) coincides with the size reduction and strain development in the crystallites. At the same time, a shift in the peak position is observed with the milling duration.

Important from the catalysis perspective is the defect region (550–600 cm^–1^), which is associated with the LO mode (F_1u_ symmetry). Even though this symmetry is Raman inactive, a relaxation of selection rules^63,64^ makes the LO mode visible. The presence of defects causes a significant lowering in symmetry and thus relaxation in selection rules. It is anticipated that ball milling tunes the oxygen defects as it will be demonstrated through the ab initio calculations (Section 3.7). Deconvolution of the Raman spectral defect region led to quantitative estimation of the O_v_/F_2g_ ratio for the pristine, DBM, and WBM Ce–La–10Cu–O oxides (Figure S4). This ratio is considered as a good descriptor of the O_v_ population in the oxide. In particular, the O_v_/F_2g_ intensity ratio was found to be 0.24 (0 h), 0.15 (2 h), 0.11 (4 h), 0.04 (6 h), 0.03 (8 h), and 0.38 (10 h) for different increasing DBM durations. In addition, Raman spectra deconvolution allowed us to spot the phase impurity (LaO_8_) as shown in Figures S3 and S4.

**Figure 3:**
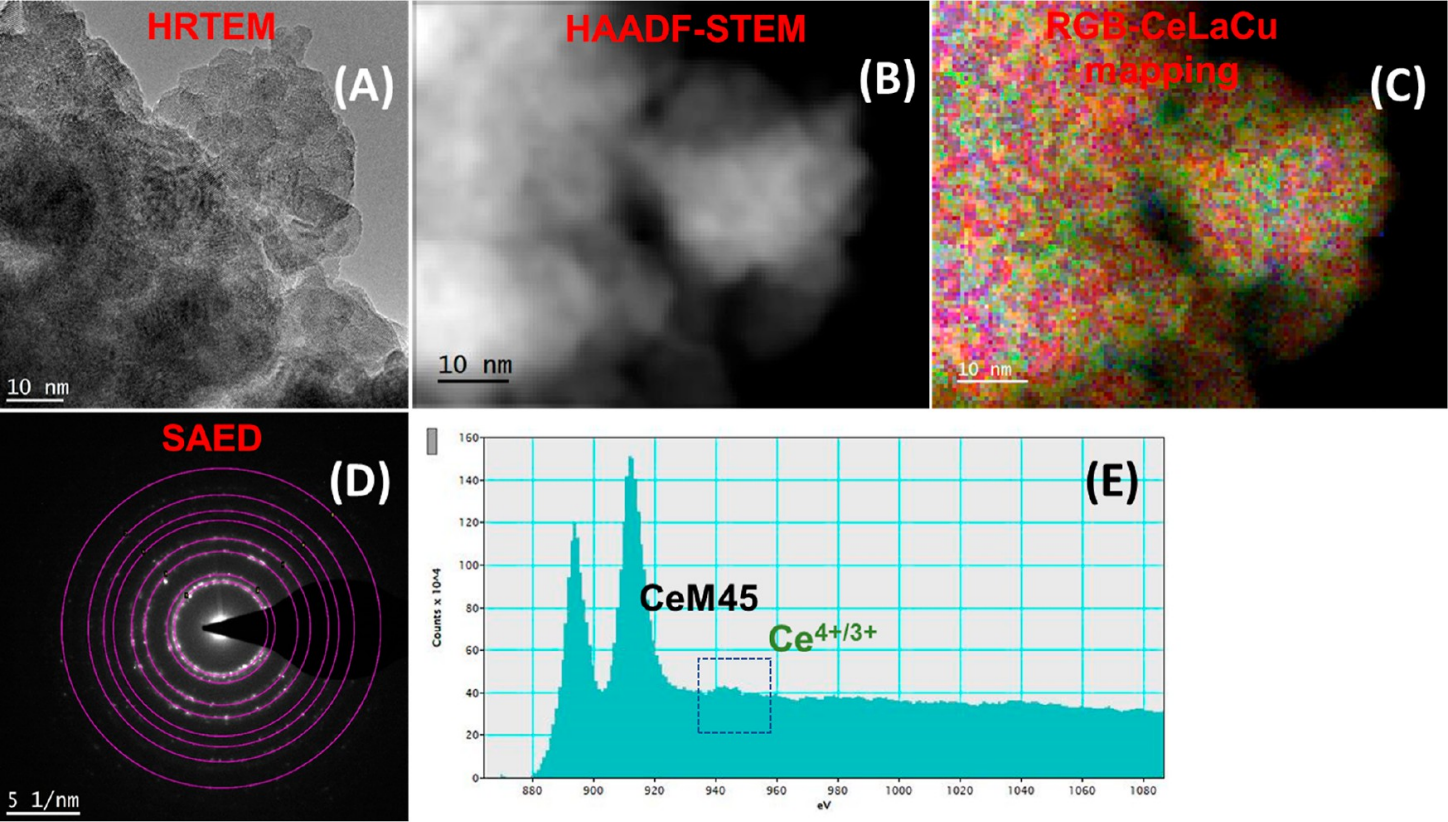
(A) HRTEM, (B) HAADF-STEM, (C) RGB mapping, (D) SAED, and (E) EELS obtained over the DBM, 4 h Ce–La–10Cu–O metal oxide.

Figures S5 and 3 show the HRTEM (A), scanning TEM high-angle annular dark-field (STEM-HAADF) (B), red-green-blue (RGB) analysis (C), selected area (electron) diffraction (SAED) (D), and electron energy loss spectroscopy (EELS) (E) micrographs of the Ce–La–10Cu–O oxide before (Figure S5) and after DBM (*t* = 4 h, Figure 3). The DBM oxide (*t* = 4 h) was chosen for this analysis due to its structural features, which make it most promising from the catalysis perspective (preservation of the fluorite ceria lattice and hence higher oxygen storage capacity). A spongy morphology with some extent of agglomeration was noticed. In the pristine ternary oxide (Figure S5), La seems to prefer to be segregated (RGB analysis, Figure S5C). Additionally, as shown in Figure S5C, La can be found as a dopant (green inside the bulk of the shown particle, area 3), as a layer surrounding the surface of the particle (decorator, area indicated by red arrows), and as a segregate (pure green upper left, area 1). The much lower Cu content (10 at. %) secures that the role of copper is mostly as a dopant into the ceria lattice. This is reflected through the purple color in the RGB image (purple as a result of the R (red) + B (blue) combination, area 2 in Figure S5C). Dopant distribution is most likely nonuniform. The above inhomogeneity in the nanoscale is in agreement with the results obtained from powder XRD and Raman; the latter showed the formation of the LaO_8_ phase impurity peak. Additionally, line-scan EDX data (two lines, (i) and (ii)) are presented in Figure S5C(i,ii) aligned with the above La and Cu role.

Following ball milling, La and Cu dopants are fairly dispersed over the ceria catalyst. As demonstrated, all materials are polycrystalline with high crystallinity, based on the SAED studies (Figure 3D and Tables S2 and S3) and in agreement with the XRD studies. Crystallinity is also maintained after ball milling. Compared to the pristine one, after DBM, a more homogeneous mixing of Ce, La, and Cu is achieved based on the RGB analysis (compare Figures 3C and S5C). Ce M4,5 EELS edges (Figure 3E) also show a chemical shift to a lower energy and change of the fine structure corroborating the presence of Ce^3+^, while Ce^4+^ is predominant in the pristine oxide (Figure S5E). This is an important finding as it confirms the contribution of milling process to the M–O bond breaking and formation of oxygen vacancies (O_v_), which always accompany the presence of Ce^3+^ oxidation state. This result is in agreement with the EPR results presented earlier, and with the DFT estimated Ce–O bond lengths under 0, −5, and +5% strain levels (Table S1). Analysis of the SAED ring structure (Tables S2 and S3) is in good agreement with the fluorite lattice.

**Figure 4:**
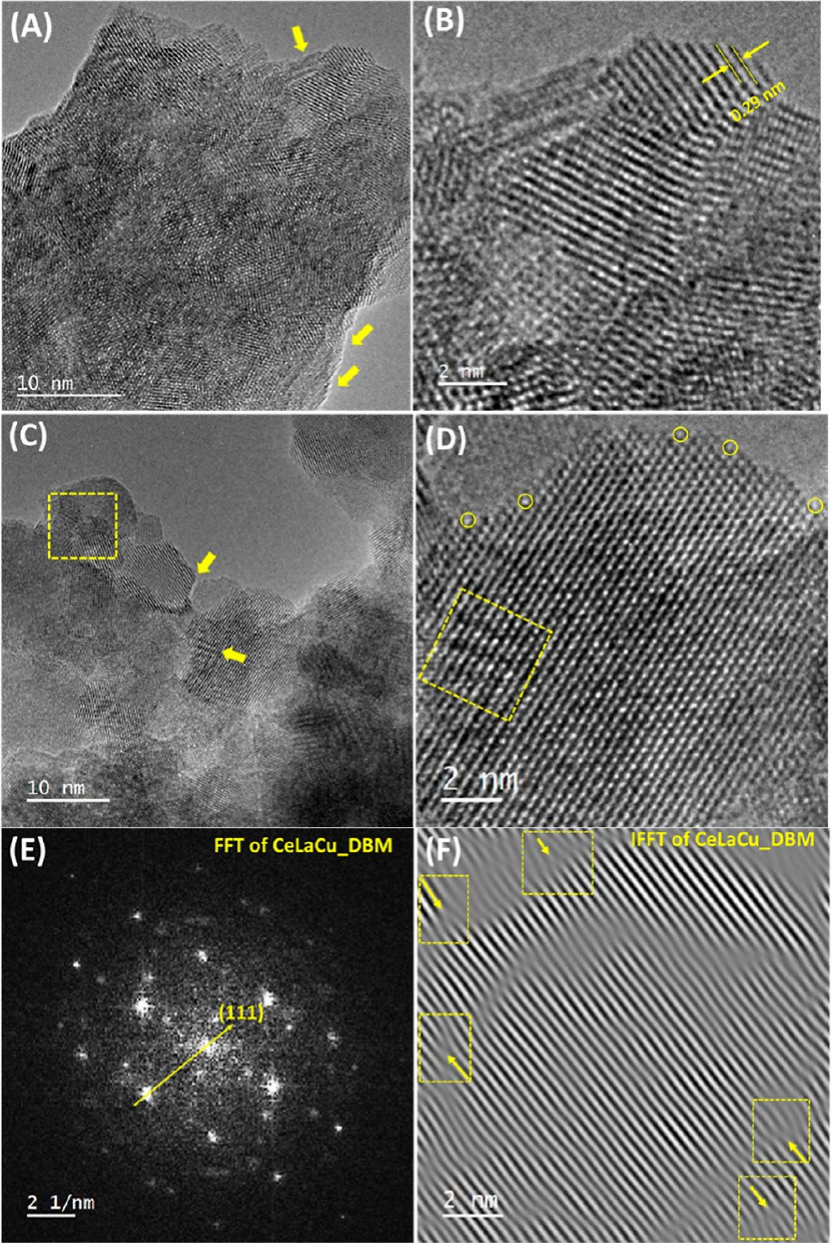
(A,B) HRTEM of the pristine CeLaCuO; (C,D) HRTEM of the DBM CeLaCuO; (E) fast Fourier transformed (FFT) analysis of (B); and (F) inverse FFT analysis of (B) across the planes (111).

An insightful look and analysis of the pristine and DBM oxides is presented in Figure 4, where the dislocations (Figure 4F areas enclosed in boxes), atomic mobility (Figure 4D, areas enclosed in circles), and local refaceting (compare Figure 4A vs Figure 4C) are demonstrated. Such features are more intense in the case of DBM oxide (Figure 4C,D). The surface steps shown in the image demonstrate the surface/atomic mobility. In the ball-milled sample (DBM), faceting appears as the particle size is small (<10 nm, i.e., 5–8 nm) with sharp edges (shown with yellow arrows), whereas the pristine sample is smoother (Figure 4A,B). Additionally, based on the SAED data, in the case of pristine oxide (Figure S5D), the complete rings showcase the presence of more crystalline material exhibiting larger in size crystals.

### 3.2 Textural Studies

It is noticed that the mechanochemical treatment of Ce–La–10Cu–O oxide yields to at least 100% increase in the specific surface area (m^2^ g^–1^) following both DBM and WBM conditions (Table 1 and Figure S6). The improvement of such textural property is consistent with previous studies, suggesting that the ball milling process facilitates the formation of more micropores and particle size reduction, particularly under dry conditions (harsh ones) due to the mechanical forces exerted on the grains.^65,66^ The porosity trends for the reference oxides are provided in the Supporting Information (Figure S6).

### 3.3 Redox Properties

**Figure 5:**
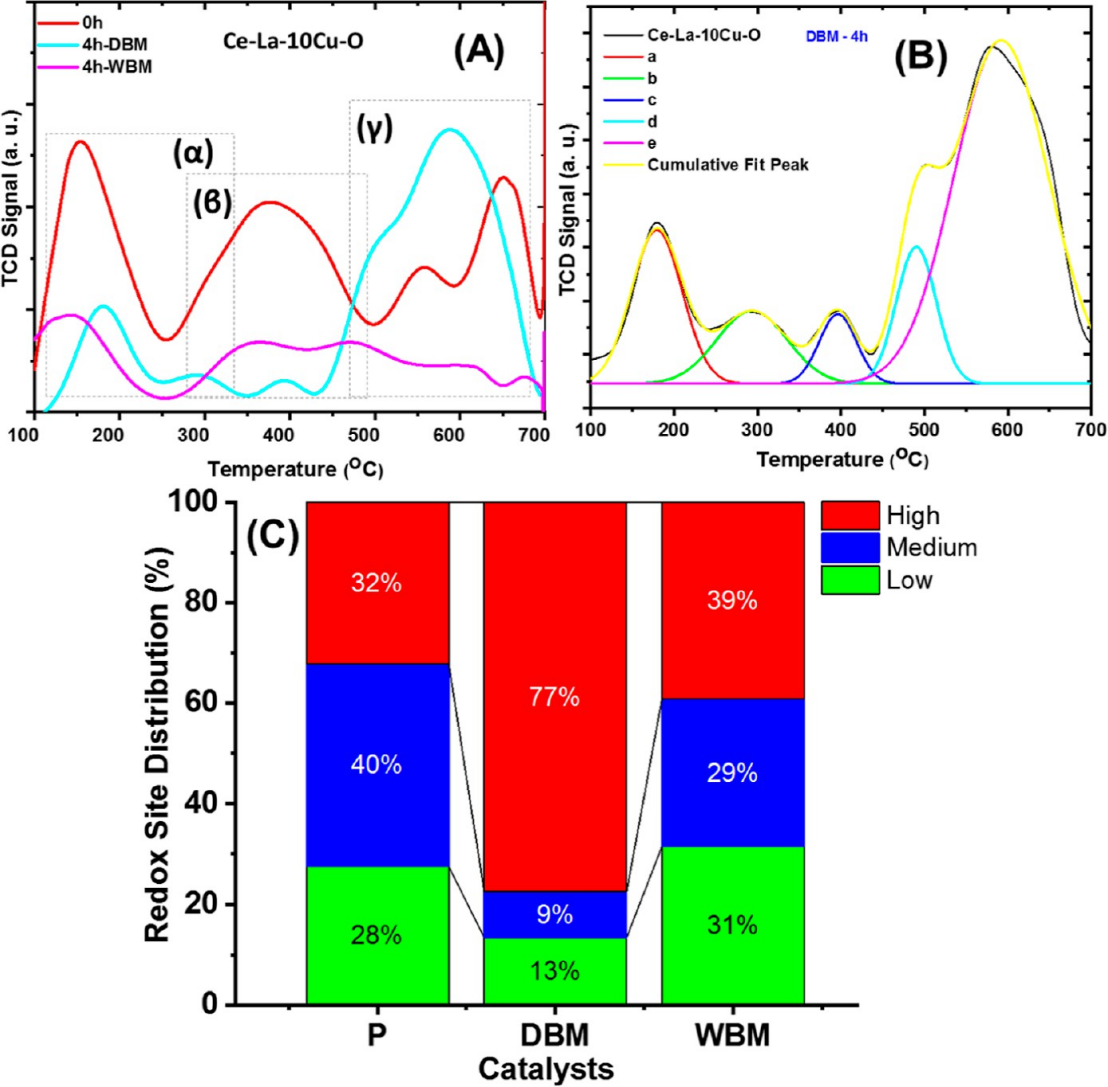
(A) H_2_-TPR profiles of pristine-, DBM-, and WBM-treated Ce–La–10Cu–O oxides; (B) deconvoluted TPR profile of DBM-treated Ce–La–10Cu–O oxide; and (C) redox site distribution over the pristine, DBM, and WBM oxides in the low-, medium-, and high-temperature regime.

Figure 5A presents the H_2_-TPR profiles of Ce–La–10Cu–O before (*t* = 0 h) and after the mechanochemical treatment in dry (DBM) and wet (WBM) atmospheres (*t* = 4 h). Ball milling after 4 h was only investigated since these conditions showed the preferred structural characteristics (based on XRD and Raman studies) among the wet ball and dry ball conditions. A profile with multiple peaks in three reduction regimes is observed, namely, *T* < 250 °C (region α), 250 °C < *T* < 500 °C (region β), and 500 °C < *T* < 700 °C (region γ). Interestingly, the pristine sample (ε_c_ only) presents larger concentration of easily reduced oxygen by hydrogen (increased mobility) in the regions α and β, whereas DBM (ε_C_ + ε_M_) increases the solid’s reducibility at higher temperatures (region γ), as reflected by the relative amounts of H_2_ consumed (mmol/g) (Figure 5C). This demonstrates that the chemical strain activates mostly the easily reducible oxygen species, whereas the presence of mechanical strain contributes to the activation of the hardly reducible ones. The latter is important as a criterion of catalyst design for reactions taking place in the *T* > 500 °C region (e.g., DRM). The enhancement of the redox properties following mechanochemical treatment is in agreement with the study of Yang et al.^67^ who compared the as-received commercial MnO_2_ with a modified one in a top-down ball milling approach. Nonetheless, an increase in the concentration of oxygen vacancies was observed, which ultimately boosted the reactivity and mobility of surface lattice oxygen. The above results can be understood on the theoretical basis of the ball milling process, where it was reported that high temperatures (>1000 °C) can be developed for periods of time in the scale of 10^–3^ to 10^–4^ s due to the friction between the balls and the materials. This leads to the formation of local hot spots which can drive the displacement of O^lattice^ species leading to the formation of O_v_^3^.

Deconvolution of the H_2_-TPR profile for the Ce–La–10Cu–O oxide following DBM for 4 h is presented in Figure 5B. The H_2_-TPR profiles for the reference samples are given in Figure S7. After calibrating the TCD signal in the H_2_-TPR experiments for the Ce–La–10Cu–O-pristine, Ce–La–10Cu–O (DBM, 4 h), and Ce–La–10Cu–O (WBM, 4 h) solids, the amount of H_2_ consumed was found to be 4.5, 11.1, and 3.2 mmol/g, respectively, demonstrating the increase of the redox sites when both chemical and mechanical strains are present (DBM solid). Considering the SSA (m^2^/g) of each solid, the density of the redox sites was estimated to be 0.92, 1.4, and 0.22 mmol/m^2^ for Ce–La–10Cu–O (pristine), Ce–La–10Cu–O (DBM, 4 h), and Ce–La–10Cu–O (WBM, 4 h), respectively.

### 3.4 Surface Analysis (XPS)

**Figure 6:**
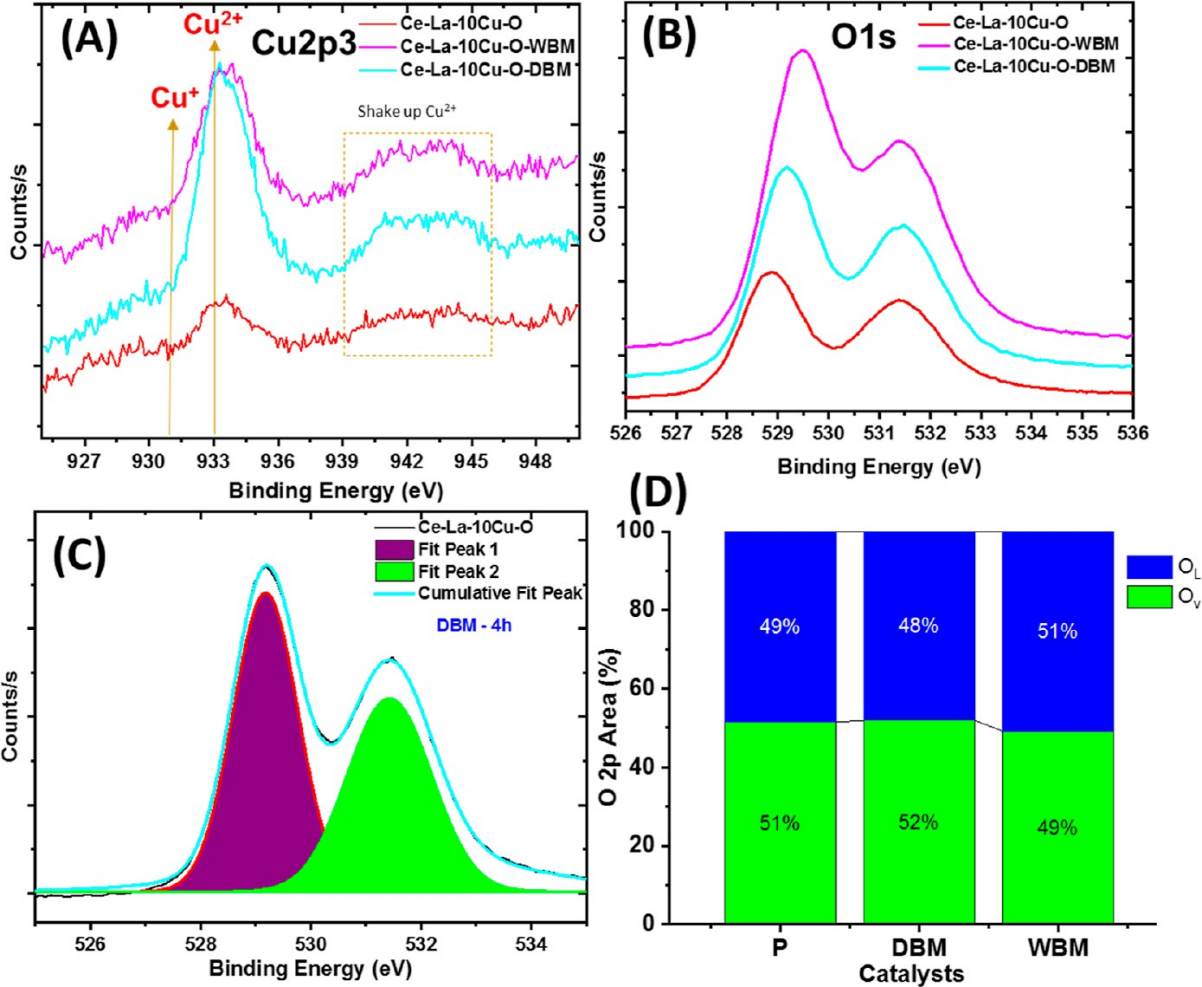
(A) Cu 2p core-level spectra for pristine and ball-milled Ce–La–10Cu–O oxides; (B) O 1s core-level spectra for the pristine and ball-milled Ce–La–10Cu–O oxides; (C) deconvoluted O 1s spectrum of the Ce–La–10Cu–O following DBM; and (D) calculated O 2p areas as per the deconvoluted spectra in (C).

Figure 6 presents the XPS Cu 2p (Figure 6A) and O 1s (Figure 6B) core-level spectra of the oxides before and after WBM and DBM treatment. The Ce 3d along with the O 1s deconvoluted spectra for pristine, DBM, and WBM oxides are presented in Figures S8 and S9. According to the literature,^68^ Cu (Figure 6A) exists in the 2+ oxidation state, which is evident by the broad satellite peak of Cu 2p_3/2_ arising at the high binding energy side (940–945 eV), clearly noticed after DBM and WBM modification. In the case of Ce 3d, all the intense peaks, namely, v0, v′, and v″ of Ce 3d_5/2_ and u0, u′, and u″ of Ce 3d_3/2_ spectra (Figures S8 and S9), are attributed to Ce^4+^, indicating that the dominant species is Ce^4+^ in all solids. As ceria exhibits high redox properties, it is useful to understand the effect of doping and mechanochemical treatment on the formation of Ce^3+^ species. According to Ardelean et al.,^69^ the shoulder peaks appearing at ∼ 885 and 898 eV, labeled as v and u, respectively, are associated with Ce^3+^ species, and their intensities relatively decrease upon doping. Interestingly, the intensities of Ce^3+^ peaks increase after the mechanochemical treatment, which is consistent with the fact that the mechanical forces induce defects, such as oxygen vacancies. For the O 1s spectra (Figure 6B,C), two components can be traced; the one at ∼529 eV corresponds to lattice oxygen,^68^ whereas the one at ∼531.5 eV can be associated with surface hydroxyl species as well as carbonate or polarized O^2–^ ions surrounding the O vacant sites.^59^ There are several reports associating this peak with neighbor to vacancies, as vacancies themselves cannot be traced using XPS.^70,71^ It is observed that after mechanochemical treatment, there is a slight shift in the 529 eV peak maximum due to the change of the environment of the lattice oxygen and the introduced lattice distortion (in agreement with XRD and Raman studies). The calculated area proportion at 531.5 eV for these materials follows the order: WBM > DBM > P (Figure 6D). Given the structural studies performed (XRD and Raman), it can be stated that in the case of WBM oxide, the predominance of oxygen species at 531.5 eV is originated from OH species rather than neighbor to vacancies species.

### 3.5 Oxygen Mobility Studies

As the lattice *oxygen mobility* is a descriptor for the surface reactivity of an oxide and the potential of oxygen vacancy formation, it is important to get an insight into the lattice oxygen (O_L_) mobility (diffusivity). The oxides of this study were tested for their ^16^O/^18^O exchange through TIIE (^18^O_2_-TIIE) experiments conducted at 350 °C. The temperature was chosen based on the catalytic reactions’ operational window. The process is governed by the descriptor functions described in Section 2.2. Differences in the *transient kinetic rate profiles* associated with these descriptors are mainly due to the differences in their oxygen sublattice structure [118], as also traced using Raman and H_2_-TPR techniques. It should be noted, however, that ^18^O_2_-TIIE was recently illustrated to be more sensitive to subtle structural differences compared to Raman spectroscopy.^28,72^

**Figure 7:**
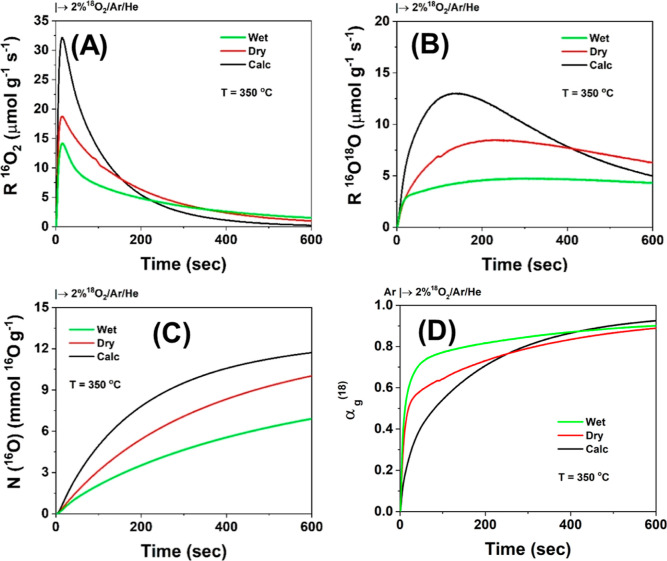
(A) Transient rates (μmol g^–1^ s^–1^) of ^16^O_2_ formation as a function of time estimated from the TIIE experiment: 2 mol % ^16^O_2_/2 mol % Kr/Ar/He (350 °C, 30 min) → 2 mol % ^18^O_2_/Ar/He (350 °C, *t*) for the Ce–La–10Cu–O oxides. *W* = 0.02 g; *F* = 50 N mL/min; (B) transient rates (μmol g^–1^ s^–1^) of ^16^O^18^O formation as a function of time estimated from the TIIE experiment: 2 mol % ^16^O_2_/2 mol % Kr/Ar/He (350 °C, 30 min) → 2 mol % ^18^O_2_/Ar/He (350 °C, 15 min) for Ce–La–10Cu–O solids. *W* = 0.02 g; *F* = 50 N mL/min; (C) evolution of the total amount of ^16^O exchange (*N*(^16^O), mmol g^–1^) as a function of time estimated from the TIIE experiment: 2 mol % ^16^O_2_/2 mol % Kr/Ar/He (350 °C, 30 min) → 2 mol % ^18^O_2_/Ar/He (350 °C, 15 min) for the Ce–La–10Cu–O oxides. *W* = 0.02 g; *F* = 50 N mL/min; (D) α_g_^(18)^ descriptor parameter as a function of time (*t*) estimated from the TIIE experiment: 2 mol % ^16^O_2_/2 mol % Kr/Ar/He (350 °C, 30 min) → 2 mol % ^18^O_2_/Ar/He (350 °C, 15 min) for the Ce–La–Cu–O oxides. *W* = 0.02 g; *F* = 50 N mL/min.

Figure 7A presents the R^16^O_2_ (μmol g^–1^ s^–1^) transient response curves for which *R*_max_ values of 14, 17, and 31 μmol g^–1^ s^–1^ were estimated for the WBM, DBM, and pristine samples, respectively. It can be seen clearly that the exchange starts immediately after the step-gas switch to 2% ^18^O_2_/Ar/He and reaches a maximum rate at a relatively short time; the *t*_max_ values for ^16^O_2_ dynamic evolution are 14.3, 16.1, and 15.7 s for pristine, DBM, and WBM, respectively. All these times are much shorter than the *t*_max_ of ^16^O^18^O (s) dynamic rate as discussed in what follows. These initial rates of ^16^O/^18^O exchange reflect the surface exchange, while bulk oxygen ^16^O/^18^O exchange via diffusion occurring at longer times is largely related to the *t*_max_ value recorded.

Figure 7B presents the transient ^16^O/^18^O exchange rate toward the ^16^O^18^O formation (*R*^16^O^18^O) as a function of time at 350 °C. It is illustrated that the exchange rate starts immediately after the switch to the isotopic gas mixture and passes through a maximum at larger times (*t*_max_ > 100 s) compared to the ^16^O_2_ formation rate for all the oxides studied herein. This result indicates that *bulk diffusion* of ^16^O toward the surface to combine with ^18^O on the surface is slow, and this diffusion-limited process is clearly different among the three oxides studied (pristine, DBM, and WBM). For the pristine oxide, the *R*_max_(^16^O^18^O) value is 13.1 μmol g^–1^ s^–1^, while following DBM and WBM treatment, it takes the values of 8.5 and 4.8 μmol g^–1^ s^–1^, respectively. The 35% reduction in the *R*_max_(^16^O^18^O) value after DBM can be understood on the basis of defect formation that slow down the oxygen diffusion, acting themselves as mechanical boundaries/barriers. The *t*_max_^16^O^18^O value is 147.2 s for pristine, 229.1 s for DBM, and 303.2 s for WBM. These results demonstrate that the oxygen binding strength of the Ce–O–La, Ce–O–Cu, and Cu–O–La local environments is modified after ball milling (co-presence of chemical and mechanical strain) most likely due to the structural disturbances introduced through the mechanical forces applied. The extent of this modification depends on the closest neighbor (Figure 1C,D).

Figure 7C displays the transient evolution with time of the total amount of ^16^O (*N*, mmol g^–1^) able to exchange with gaseous ^18^O_2_ at 350 °C. The observed differences in the *N*^16^O(*t*) descriptor function for the samples under study reflect the differences in the intrinsic transient kinetic rate of subsurface lattice ^16^O diffusion. The latter depends on the structure of the oxygen sublattice and the local energy barriers encountered for oxygen mobility, which are determined by the chemical composition of the solid. It has been reported^28,73^ that oxygen diffusion via grain boundaries on ceria-based catalysts is much faster than the bulk oxygen diffusion. It is also noted that the total amount of ^16^O species, *N*(^16^O), exchanged varied in the 8.2–12.4 mmol O g^–1^ range (see Table 2) for the pristine, DBM, and WBM oxides).

**Table 2:** Single-Value Parameters Derived from the Transient Response Curves Recorded during the TIIE Experiment: 2 mol % ^16^O_2_/2 mol % Kr/Ar/He (350 °C, 30 min) → 2 mol % ^18^O_2_/Ar/He (350 °C, *t*) over the Ce–La–10Cu–O Samples^a^

	oxide		
parameter	pristine	DBM	WBM
*R*_max_^16^O_2_ (μmol g^–1^ s^–1^)	32.2	18.7	14.2
*R*_max_^16^O^18^O (μmol g^–1^ s^–1^)	13.1	8.5	4.8
*t*_max_^16^O_2_ (s)	14.3	16.1	15.7
*t*_max_^16^O^18^O (s)	147.2	229.1	303.2
*N*(^16^O) (mmol^16^O g^–1^)	12.4	11.4	8.2

a *W* = 0.02 g; *F* = 50 N mL/min.

Figure 7D presents the dynamic evolution of the α_g_^(18)^(*t*) descriptor parameter (see eq 5) with time upon exposure of the solids in the 2 mol % ^18^O_2_ isotopic gas mixture. The higher the value of this parameter, the slower the exchange of lattice oxygen with ^18^O_2_, thus the smaller oxygen mobility (effective diffusivity) in the oxygen sublattice of the solid. It is shown that ball milling deteriorates oxygen mobility compared to the pristine Ce–La–10Cu–O metal oxide according to the α_g_^(18)^(*t*) transient response curves (see the first 300 s of the transient). These results are consistent with those of Figure 7B, where larger *t*_max_ is recorded for WBM compared to the other solids.^28^ Furthermore, the pristine sample (chemically strained) appears to possess more labile surface and bulk lattice oxygen species (Figure 7D) in harmony with the H_2_-TPR results (Figure 5), where the same solid showed the lowest concentration of high-temperature reducible lattice oxygen species.

### 3.6 CO_2_ Metal Oxide–Surface Interaction

#### 3.6.1 Probing CO_2_-Philicity Using Thermal Desorption

**Figure 8:**
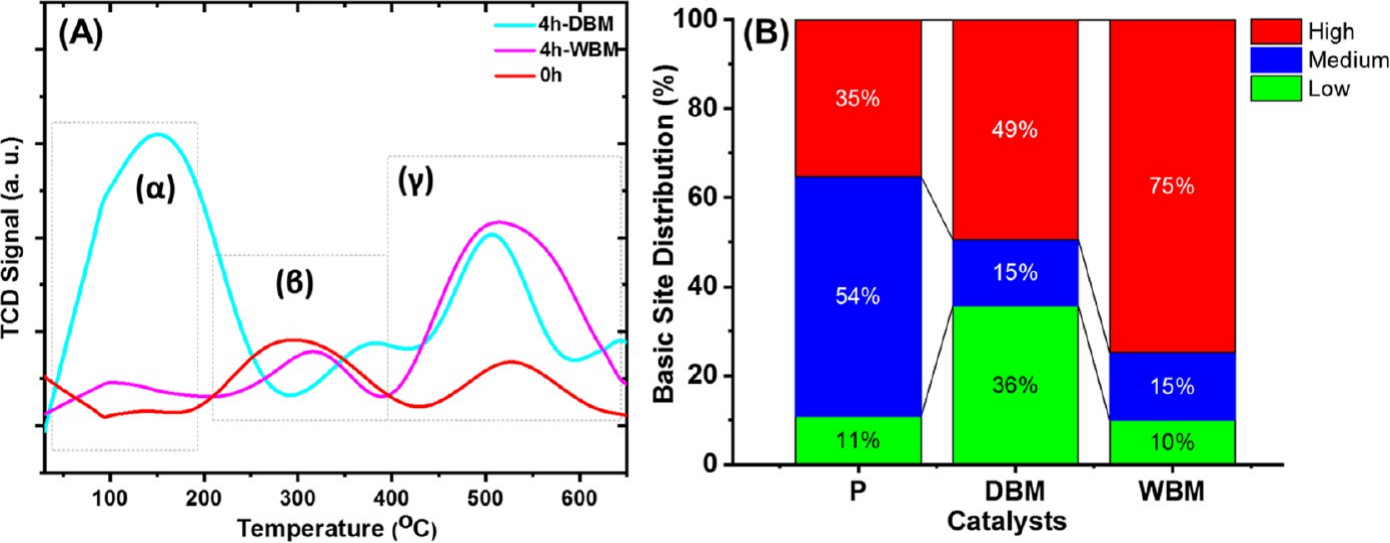
(A) CO_2_-TPD profiles over pristine, DBM, and WBM Ce–La–10Cu–O oxides and (B) distribution of basic sites (%) in the low, medium, and high strength basicity regions over the pristine (P), DBM, and WBM metal oxides.

Figure 8A presents the CO_2_-TPD profiles obtained over Ce–La–Cu–O pristine, DBM, and WBM, while those for the reference oxides are given in Figure S10. Generally, it is well known that CO_2_ adsorption and desorption probe the strength of the basic sites (M^δ+^–O^δ−^ entities) on the surface. The peaks observed at temperatures below 200 °C correspond to weak basic sites, the peaks at 200–450 °C represent moderate basic sites, while peaks above 450 °C are linked to strong basic sites.^74^Figure 8 depicts the CO_2_-TPD profiles over the pristine, DBM, and WBM Ce–La–10Cu–O oxides, aiming to probe the modifications induced by ball milling (chemical and mechanical strain) on the CO_2_-philic sites on the surface. Calibrating the CO_2_-TPD signal for the Ce–La–10Cu–O-pristine, Ce–La–10Cu–O (DBM, 4 h), and Ce–La–10Cu–O (WBM, 4 h) solids, the total amount of CO_2_ adsorbed on the surface was found to be 1.48 (0.30), 6.03 (0.74), and 3.78 (0.26) mmol/g (mmol/m^2^), respectively. The numbers in parentheses correspond to basic sites concentration per unit surface area. As can be seen, the DBM enriches the surface basicity overall, while the WBM induces milder modification at temperatures below 250 °C and a redistribution of the CO_2_-philic sites. In particular, the DBM increases the concentration of low- and high-strength basic sites, while it reduces that of the medium-strength basic sites (Figure 8B). It is anticipated that ball milling through the imposed mechanochemical forces causes reduction of the particle size, as well as structural disordering, thus creating more adsorption sites per gram basis, as proved in what follows.

It has been reported that Lewis acidity (e^–^ acceptor characteristic) and basicity (e^–^ donor characteristic) have been correlated with the differences in powder processing method, as they can affect the triboelectric phenomenon,^75^ powder flow,^76^ and other surface and powder properties.^77^ The milling process has been correlated with the increase of electron-donor characteristic of the surface and the increase of basic sites due to introduced defects, leading to higher oxidation activity.^78^

#### 3.6.2 Vibrational Spectroscopy Tools

**Figure 9:**
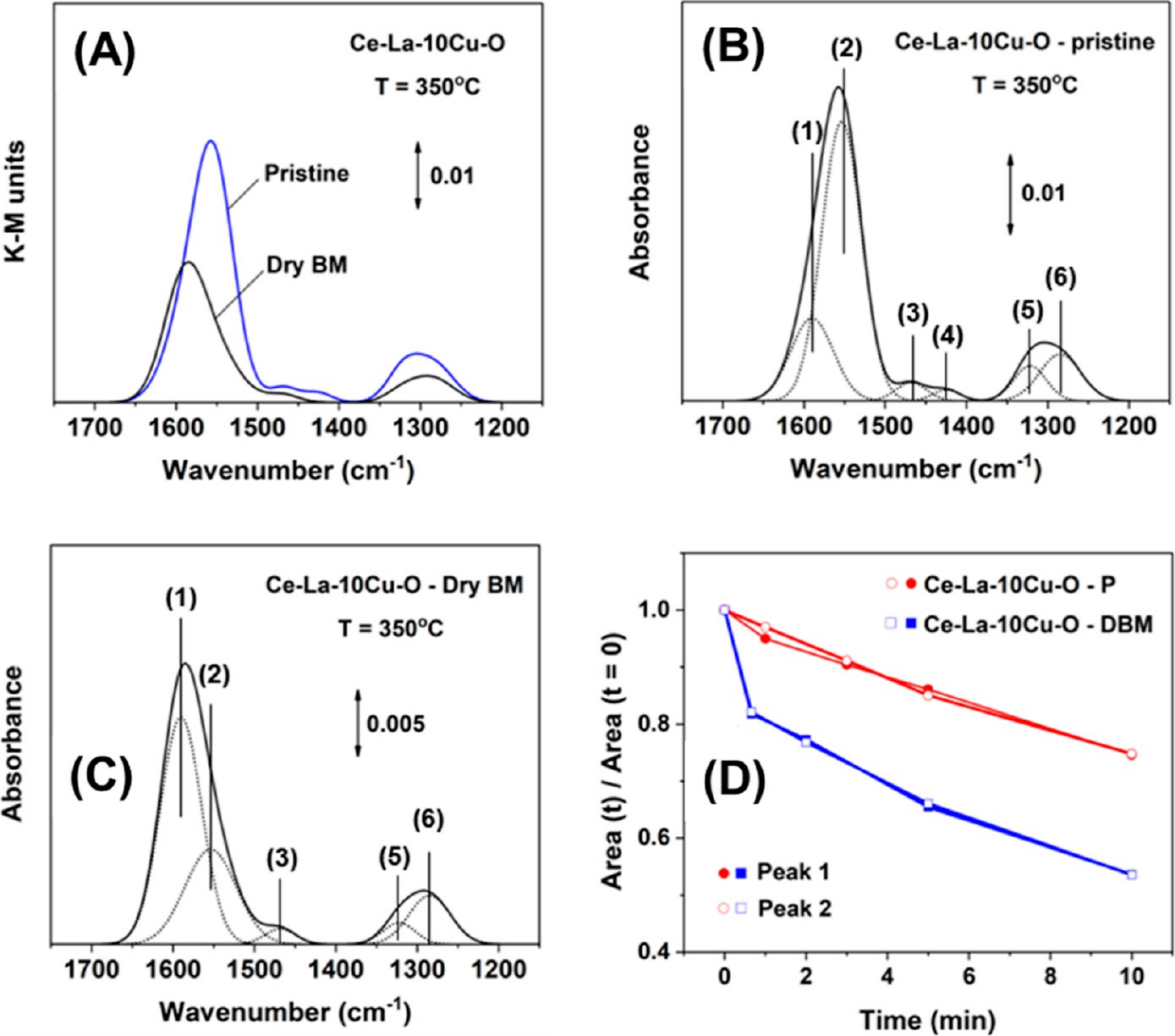
(A) DRIFTS spectra recorded under 5 vol%CO_2_/He (30 min) gas mixture in the 1750–1150 cm^–1^ range over Ce–La–10Cu–O—DBM and Ce–La–10Cu–O—pristine solids. Deconvoluted IR spectra of Ce–La–10Cu–O—pristine (B) and Ce–La–10Cu–O—DBM (C) solids. (D) Ratio of area(*t*)/area(*t* = 0) related to peak 1 and peak 2 for both the pristine and DBM materials.

To monitor the CO_2_ activation pathway over the two surfaces of the most interest (pristine and DBM), in situ CO_2_-DRIFT spectra were recorded at 350 °C on the unreduced catalysts as shown in Figures 9 and S11. The assignment of the adsorbed CO_2_ IR bands was based on the open literature. Figure 9A presents the DRIFTS spectra in K–M units (1750–1150 cm^–1^ range) recorded under the 5 vol % CO_2_/He gas mixture and after 30 min on stream over the Ce–La–10Cu–O-DBM and Ce–La–10Cu–O-pristine metal oxides. The K–M units allow for a quantitative interpretation of the bands’ intensities recorded.

After spectra deconvolution, both samples show IR bands at 1590 cm^–1^ (peak 1) and 1554 cm^–1^ (peak 2), which correspond to bidentate carbonate species (Figure 9B,C). A closer look at the CO_2_ adsorption IR bands for the pristine oxide (chemically strained) (Figure 9B) gives the IR band recorded at 1554 cm^–1^ as the predominant one (area_Peak1_/area_Peak2_ = 0.32). Following the DBM process, where both chemical and mechanical strain co-exist, the surface chemistry apparently changes, and the predominant IR band becomes the one at 1590 cm^–1^ (Figure 9C; area_Peak1_/area_Peak2_ = 1.96). According to the literature, the IR band recorded at 1554 cm^–1^ over unreduced ceria shifts to higher wavenumbers (1590 cm^–1^) over a reduced CeO_2_, and after reoxidation, the IR band shifts back to 1554 cm^–1^.^78^ The areas ratio of peak 1 to peak 2 for the pristine sample was found to be 0.32, and for DBM 1.96, indicating that the DBM process (addition of mechanical strain) leads to an increase in the surface concentration of defects/CO_2_ adsorption sites by ∼6 times compared to pristine (chemical strain only). The IR band at 1468 cm^–1^ (peak 3) and 1425 cm^–1^ (peak 4) can be assigned to polydentate carbonates and bicarbonate species, respectively.^79^ The IR bands at 1322 cm^–1^ (peak 5) and 1285 cm^–1^ (peak 6) correspond to carbonates and bidentate carbonates, respectively.^80^ These CO_2_ adsorption-DRIFTS experiments were performed at 350 °C, where medium-strength basic sites were present for both the pristine and milled solids. The concentration of medium-strength basic sites is higher for pristine compared to DBM (Figure 8B), in agreement with the DRIFTS results (Figure 9A).

The thermal stability of the adsorbed species formed after CO_2_ interaction at 350 °C can be studied based on the data reported in Figures 9D and S11. The IR bands for both materials decrease with time in Ar gas flow following a 30 min CO_2_ gas treatment (Figure S11). The ratio in the integral band area of peak 1 and peak 2 recorded after 30 min in CO_2_/Ar to that after 10 min in Ar gas flow remains the same for both samples, indicating that the IR band at 1590 cm^–1^ was not shifted to lower wavenumbers (1554 cm^–1^) and that both materials were not reoxidized. Figure 9D shows the dynamic evolution at 350 °C of the ratio of areas of peak 1 or peak 2 under Ar gas flow to that at time zero (under CO_2_/He gas mixture). As illustrated in Figure 9D, more stable carbonates are formed over the pristine (chemically strained) compared to the DBM (chemically and mechanically strained) solids. This thermal stability should be related to the CO_2_–surface interaction as described using the theoretical approach. Additionally, upon the CO_2_ adsorption on the surface, the O–C–O angle for 0, 5, and −5% was found to be 120.04, 119.88, and 124.75°, respectively. The value for the tensile strain (119.88°) is more realistic as discussed earlier, that upon DBM, tensile strain was developed in the solids (Table 1). However, the difference between the two angles (120.04 vs 119.88°) is insignificant and hence not conclusive. Furthermore, the C_CO_2__–O_surface_ distance (Å) (Tables S19 and S21) was found to be shorter under 5% tensile strain compared to zero and compressive strain, corroborating for a higher interaction/CT in the tensile strain conditions. This difference is more pronounced in the case of CeO_2_ and Ce–La–O but not in the case of Ce–La–Cu–O solid. However, it is noteworthy that the two angles O_surf_–C–O_1_ and O_surf_–C–O_2_ (O_1_ and O_2_: the two CO_2_-derived oxygen atoms) exhibit the highest difference for Ce–La–Cu–O under 5% tensile strain compared to zero strain for the same solid (pristine). This inequality in the two angles supports the different contribution of the O_1_ and O_2_ atoms of the CO_2_ molecule in the adsorbed state in pristine and DBM solids, leading to different thermal stabilities of the formed carbonates. Furthermore, the energy of CO_2_ adsorption was found to be −1.33 and −1.15 eV for the zero and +5% (tensile) strain cases. This is a strong indication for the higher stability of carbonates over the chemically strained surface (Ce–La–Cu–O, pristine) and for the role of mechanical strain (tensile) to engineer the CO_2_ adsorption sites might be through structural reconstruction. In addition, the higher population of medium-strength basic sites on the pristine surface, over which the bidentate carbonates are formed, gives rise to a higher population of carbonates on the surface, thus prolonging their presence under thermal treatment.

### 3.7 DFT Studies

#### 3.7.1 Energy of Oxygen Vacancy Formation (*E*_O_v__^f^)

##### 3.7.1.1 O_v_ Chemical Environment

**Figure 10:**
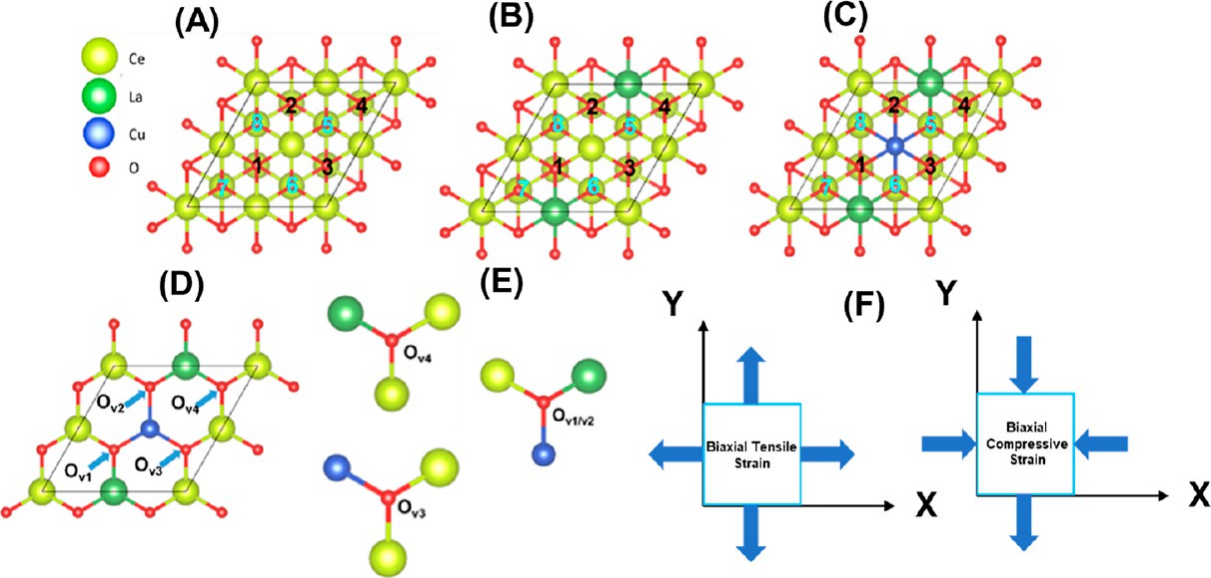
Schematic representation of oxygen vacancy (O_v_) created in the uppermost surface (black) and subsurface (cyan): (A) pure CeO_2_(111), (B) La-doped CeO_2_ (Ce–La–O(111)), (C) La and Cu co-doped CeO_2_ (Ce–La–Cu–O(111)), (D,E) first neighbor configurations of the possible oxygen vacancies in Ce–La–Cu–O(111), and (F) demonstration of the compressive and tensile biaxial strain.

Oxygen vacancies at different chemical environments (locations) in pure ceria and doped system (Ce–La–O and Ce–La–Cu–O) slab were created as explained in Section 2.5 (see Figure 10 and Table 3) to have a qualitative comparison and deeper insights regarding lattice strain (doping effect) and biaxial strain (external stimulus) effects on the defective systems’ stability. The pure CeO_2_(111) depicted in Figure 10A was doped with La in the first layer and labeled Ce–La–O, which is shown in Figure 10B. Figure 10C illustrates the addition of copper (Cu) and lanthanum (La) to the ceria slab to resemble the oxides discussed in the experimental part (section 2.1). In this work, the same annotations as in our previous publication^59^ was followed; the single vacancies on the surface layer are denoted as black, and the vacancies generated in the first subsurface layer are represented by cyan. The following labels were used for various vacancies: single surface (1,2,3,4), single subsurface (5,6,7,8), double of surface and subsurface (combination of the corresponding locations of the vacancies, e.g., 1,7), double of surface vacancies (combination of the corresponding locations of the vacancies, e.g., 1,2), and double vacancies both occurring at the subsurface (combination of the corresponding locations of the vacancies, e.g., 6,8). These distinct scenarios of surface defects were considered (see Table 3), and their effect on the slabs’ stability was systematically investigated. All the obtained values are provided in Tables S4–S18.

**Table 3:** Configurations of Oxygen Vacancies (O_v_) Considered for the DFT Calculations Performed in This Study

		vacancy site	
type of O_v_	notation	Ce–La–O system	Ce–La–Cu–O system
single surface (SSV)	1	1: La–□–Ce	1: La–□–Cu
	2	2: La–□–Ce	2: Cu–□–La
	3	3: Ce–□–Ce	3: Ce–□–Cu
	4	4: La–□–Ce	4: La–□–Ce
single subsurface (SSSV)	5	5: La–□–Ce	5: La–□–Cu
	6	6: La–□–Ce	6: Cu–□–La
	7	7: La–□–Ce	7: La–□–Ce
	8	8: Ce–□–Ce	8: Ce–□–Cu
double surface (DSV)	1,2	Ce–□–Ce and La–□–Ce	La–□–Ce and La–□–Ce
	3,4	Ce–□–Cu and La–□–Ce	La–□–Cu and Cu–□–La
double subsurface (DSSV)	6,8	La–□–Ce and Ce–□–Ce	Cu–□–La and Ce–□–Cu
	5,6	La–□–Ce and La–□–Ce	La–□–Cu and Cu–□–La
	7,8	La–□–Ce and Ce–□–Ce	La–□–Ce and Ce–□–Cu
	5,8	La–□–Ce and Ce–□–Ce	La–□–Cu and Ce–□–Cu
double surface and subsurface (DSSSV)	1,7	La–□–Ce and La–□–Ce	La–□–Cu and La–□–Ce
	4,7	La–□–Ce and La–□–Ce	La–□–Ce and La–□–Ce
	2,5	La–□–Ce and La–□–Ce	Cu–□–La and La–□–Cu

##### 3.7.1.2 Single Surface Vacancies

**Figure 11:**
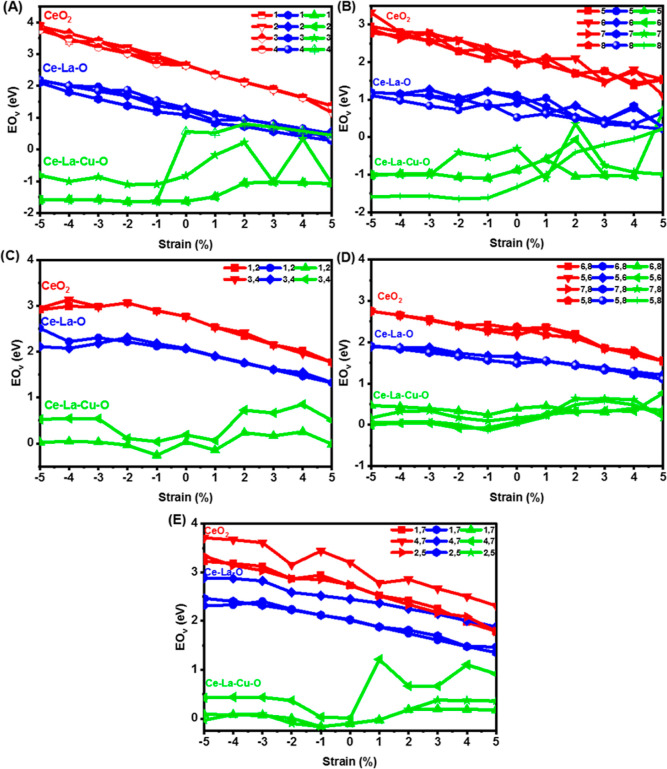
(2 × 2) CeO_2_(111) (red), Ce–La–O (blue), and Ce–La–Cu–O (green) oxygen vacancy energy of formation (*E*_O_v__^f^) under the compressive (−5 to 0%) and tensile (0–5%) biaxial strain for (A) single surface vacancy (SSV), (B) single subsurface (SSSV), (C) double surface (DSV), (D) double subsurface (DSSV), and (E) double surface and subsurface configurations (DSSSV).

Figure 11A shows that the oxygen vacancy formation energy (*E*_O_v__^f^) in the pure CeO_2_ was in the range of 2.64–2.66 eV for different O_v_ configurations at zero mechanical strain, which is in good agreement with the previous report for the zero, tensile, and compressive strain if the *p*(2 × 2) case is considered.^42^ Doping with La (Ce–La–O surface) drops the *E*_O_v__^f^ to the 1.09–1.30 eV range at zero mechanical strain, where the lowest *E*_O_v__^f^ value was calculated at the single surface vacancy (SSV) 3 (Ce–O_v_–Ce), near Ce atom, and the highest one at the La nearest neighbor (La–O_v_–Ce, configuration 2). In the case of La and Cu co-doping (SSV1), the *E*_O_v__^f^ value was further reduced to −1.62–0.56 eV. The oxygen vacancy that is located between Cu and La had the lowest energy (most stable), whereas the first neighbor vacancy to La (single surface vacancy 4 = La–O_v_–Ce) exhibited the highest energy.

##### 3.7.1.3 Mechanical (Biaxial) Strain Effect on *E*_O_v__^f^

Under the biaxial strain of ±5% in pure and doped CeO_2_, the oxygen vacancies showed a similar effect and stability. With some exceptions, the *E*_O_v__^f^ value decreases with increasing tensile strain, indicating more stability as we move from compressive (−5%) to tensile (+5%) strain. In the case of single oxygen vacancies in pure CeO_2_ at +5% strain, the *E*_O_v__^f^ value ranged between 1.15 and 1.37 eV and between 3.75 and 3.89 eV for −5% strain. A similar monotonical trend of decrease was observed upon doping CeO_2_ with La, but with lower *E*_O_v__^f^ values (more stable with increasing tensile strain, similar to pure CeO_2_). Contrary to the trend observed in pure ceria and La-doped systems, on the ternary system (Ce–La–Cu–O), the slab stability’s behavior presented an opposite trend; the *E*_O_v__^f^ values were found to be favorable at compressive strain and zero strain conditions. Despite the fluctuating nature of *E*_O_v__^f^, the values are generally increasing with tensile strain.

##### 3.7.1.4 Single Subsurface Vacancies

Figure 11B demonstrates the effect of chemical strain (doping) and biaxial strain (mechanical forces) on the single vacancies created in the first subsurface (SSSVs) of the three studied oxides. The profile of the SSSV is similar to that of the SSV, but the slope of the energy–strain curves for the SSSV is smaller (less steep) than for the SSV. For CeO_2_, under zero strain, the subsurface oxygen vacancies (SSSVs) are more favorable than the single surface vacancies (SSVs). The *E*_O_v__^f^ values are in the range of 1.95–2.2 eV and decrease with increasing tension by 1% (1.11–1.53 eV). The stability of the different configurations of the single subsurface vacancies (SSSVs) at 0, +5, and −5% strain values can be arranged ascendingly as 5 < 7 < 6 < 8, 8 < 5 < 7 < 6, and 6 < 5 < 7 < 8, respectively. A similar behavior was also observed in the Ce–La–O slab’s case under zero strain but with lower formation energy values (0.53–1.11 eV). The highest *E*_O_v__^f^ values were observed in the case of SSSV 7 (La–O_v_–Ce), while the lowest ones were for SSSV 8 (Ce–O_v_–Ce). The profile of *E*_O_v__^f^ remains the same for the doped Ce–La–O and undoped CeO_2_ cases under compression and tension.

The Ce–La–Cu–O system exhibited the lowest *E*_O_v__^f^ values at the subsurface sites, which indicates that oxygen vacancies are more likely to occur spontaneously after adding copper. It is well established that the *E*_O_v__^f^ values can be a qualitative descriptor of the chemical activity of an oxide surface.^81^ Among the SSSV sites 5, 6, 7, and 8, site 7 (La–O_v_–Ce) showed better stability at +5% strain. The system’s behavior fluctuated in the strain span, ca. −2–0%, showed low stability, followed by a drop in *E*_O_v__^f^ at 1% but deviating again at 2% strain. Generally, in the Ce–La–Cu–O oxide, the SSSVs 5, 6, and 8 exhibited higher stability in the compression range compared to the SSSV 7; this designates the important role of Cu being placed at an adjacent site to the O_v_.

##### 3.7.1.5 Double Surface Vacancies

Figure 11C depicts the double vacancy formation energy at the three oxides’ surface layers. Ce–La–Cu–O reached the highest stability at all the strains by achieving the lowest values of *E*_O_v__^f^ compared to pure CeO_2_ and the binary system (Ce–La–O). As an example, the double surface vacancy (DSV) 1,2 and DSV 3,4 configurations in Ce–La–Cu–O under zero mechanical strain (ε_M_ = 0) showed energies of 0.03 and 0.18 eV, respectively. The same configurations in CeO_2_ and Ce–La–O had much higher energies, ca. 2.765, 2.764, and 2.05, 2.07 eV, respectively. As previously mentioned, the higher the % tension, the higher the O_v_ stability, and this is also supported by the Ce–O bond lengths (see Table S1 and Figure 1C,D). The double vacancies (DSV) in the surface and subsurface (DSSV) slabs suggest lower stability of CeO_2_ in all ranges of tensile and compression strains applied than the single surface vacancy. Moreover, by comparing between the double vacancy configurations in CeO_2_ (Figure 11C–E), the stability appears to be the highest when both vacancies are created in the subsurface (0%: 2.17–2.37 eV) and to be the lowest when a combination of surface and subsurface vacancies are suggested (zero strain: 2.72–3.03 eV). This indicates the difficulty and the low possibility to have two O_v_ released from two different oxygen layers; however, the energies drop by 0.7–1 eV at +5% strain.

Similarly, for Ce–La–O, the double subsurface vacancy (DSSV) formation energy under zero mechanical strain (ε_M_ = 0) lies between 1.49 and 1.65 eV, while the vacancy in surface and subsurface vacancies have higher formation energies (0%: 2.01–2.65 eV) but tends to stabilize as the tensile strain increases (5%: 1.35–1.89 eV). For Ce–La–Cu–O DSSVs, the *E*_O_v__^f^ value is lower by approximately 2 eV at zero mechanical strain (ε_M_ = 0) compared to CeO_2_ and Ce–La–O (−0.10, −0.10, and 0.01 eV). It was also noticed that the behavior of the O_v_ formation energies at 5% tension was not consistent throughout the suggested configurations in the double surface (see Table S7). For instance, 1,7, 7,4, and 2,5 cases of vacancies exhibited low stability at +5% strain (0.17 eV), while 3,8, 1,5, and 2,6 showed the opposite behavior at −5% strain.

#### 3.7.2 CO_2_–Surface Interaction and Chemistry of Activation

##### 3.7.2.1 CO_2_ Adsorption Energy under Chemical Strain (ε_c_) and Zero Mechanical Strain (ε_M_ = 0)

**Figure 12:**
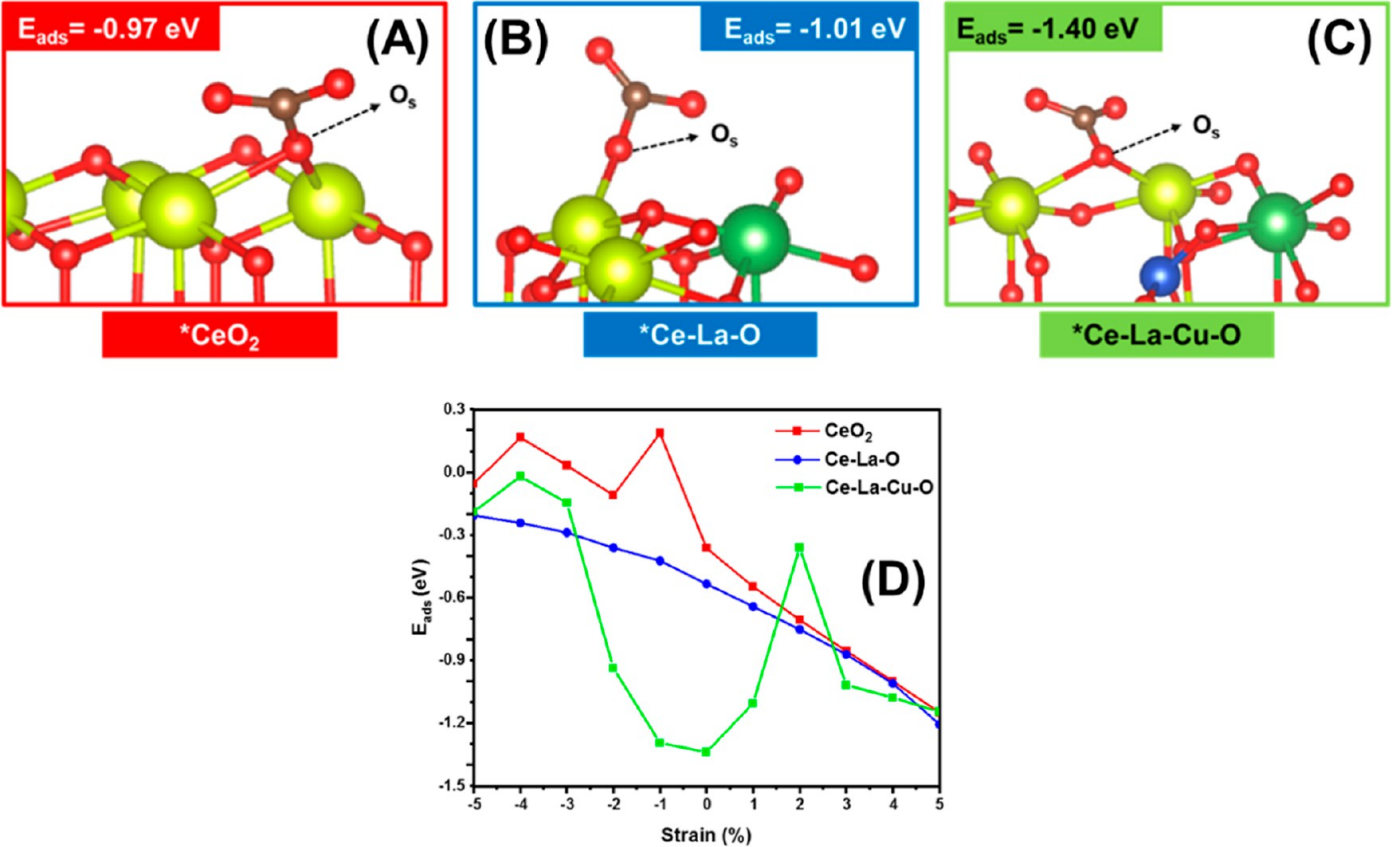
(A) CO_2_ adsorption on defective CeO_2_(111); (B,C) CO_2_ adsorption on the doped-CeO_2_(111) surfaces at site 2 at zero strain (all the surfaces are considered with surface O_v_); and (D) CO_2_ adsorption on CeO_2_(111) and doped-CeO_2_(111) surfaces under the strain effect in the absence of oxygen vacancies (O_v_).

CO_2_ adsorption calculations were performed on the pure and doped CeO_2_ surfaces (Ce–La–O and Ce–La–Cu–O) since carbon dioxide has a preference to be adsorbed on the ceria/doped ceria support during catalytic reactions.^82^ In this scenario, only the chemical strain was considered; the latter originates from the doping and the subsequent O_v_ presence. For these calculations, the oxygen vacancy on site 2, single surface vacancy (SSV 2: La–O_v_–Ce or Cu–O_v_–La) configuration was chosen based on the reported literature.^83^ This part of the study aims to enlighten the chemical strain effect on the CO_2_ adsorption, which is reflected on the doping. As clearly shown in Figure 12A,B and Table 4, La doping drops the *E*_ads_ from −0.97 eV (CeO_2_ defective structure) to −1.01 eV; La and Cu ceria co-doping further enhances the spontaneity of the CO_2_ adsorption process (*E*_ads_ = −1.40 eV) (see Figure 12C). It should be noted that the adsorption energy in the presence of the oxygen vacancy in the CeO_2_ was approximately as that reported in the literature^83^ (−0.91 eV), where CO_2_ is adsorbed to a surface oxygen (O_s_, shown in Figure 12A–C) near to the vacancy site and which ultimately forms carbonate species (CO_3_^2–^).^83^ Further calculations have been conducted on different sites which showed that CO_2_ adsorption on vacancy sites takes place with a low adsorption energy. Therefore, the rest of the calculations were established assuming the above chemical environment (adsorption site) since it was reported to be the most stable compared to the one where the CO_2_ is directly adsorbed onto an oxygen vacancy.^83^ It should be mentioned at this point that the present study did not focus on optimizing all the possible CO_2_ adsorption sites on the surface, and thus, only the linear configuration of CO_2_ was studied.

**Table 4:** Adsorption Energies and Geometric Parameters for the Activated CO_2_ Molecule on CeO_2_(111), Ce–La–O(111), and Ce–La–Cu–O(111); Bond Length (Å), C–O_surf_ (Å) Distance from the Surface, and O–C–O Angle (°) with the Presence of a Surface Oxygen Vacancy (O_v_) at Zero Strain^a^

oxide system	α_O–C–O_ (deg)	*d*_O=C_ (Å)	*d*_C–O_surf__ (Å)	*E*_ads_ (eV)
*CeO_2_(111)	128.77	1.28/1.26	1.37	–0.97
*Ce–La–O(111)	127.62	1.22/1.32	1.40	–1.01
*Ce–La–Cu–O(111)	124.71	1.25/1.31	1.34	–1.40

a CO_2_ linear configuration (L) was considered.

Based on the *E*_O_v__^f^ results presented above (Section 3.7.1), it is expected that the CO_2_ adsorption is enhanced with the presence of La, Cu dopants due to the facility of the O_v_ formation (Figure 12A), which means activation of CO_2_ will be more efficient. Figure 12 shows the activation of the linear CO_2_ molecule, which is accompanied by the decrease in the O–C–O angle and the elongation of the O=C bonds (Table 4). In particular, the intramolecular C–O bonds of the adsorbed species appeared to be elongated to 1.22–1.32 Å compared to 1.13 Å in the free CO_2_ molecule; this corresponds to a strong deformation and CT to be discussed later.

##### 3.7.2.2 CO_2_ Adsorption Energy without Oxygen Vacancies (O_v_) under the Mechanical Strain Effect (ε_M_≠ 0)

**Figure 13:**
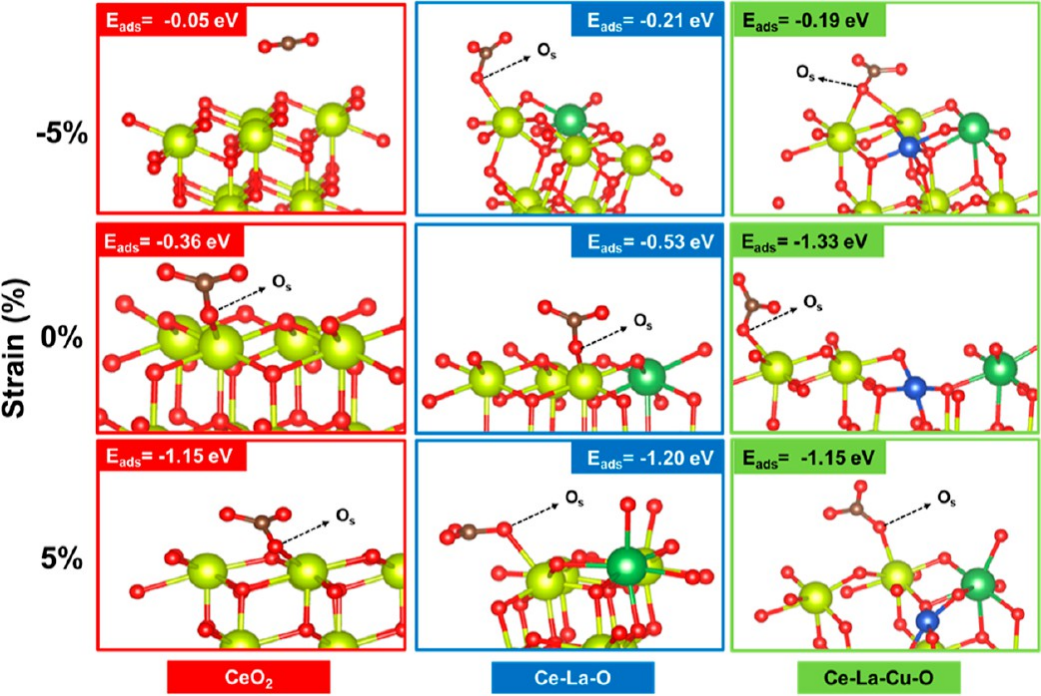
CO_2_ adsorption on CeO_2_(111) and doped CeO_2_(111) surfaces at the 0, 5, and −5% strain level in the absence of oxygen vacancies (O_v_).

CO_2_ adsorption energy (*E*_ads_) calculations were also performed on the three surfaces (CeO_2_, Ce–La–O, and Ce–La–Cu–O) in the absence of oxygen vacancies. The CO_2_ adsorption was considered under one effect at a time (e.g., doping effect vs external strain effect) in order to decouple the combined effects of each of these two factors and their role on the CO_2_ adsorption. In the herein calculations, the chosen adsorption site was also on top of a surface oxygen atom (O_surf_) as in the previous case. The calculations were carried out regarding this specific site, starting from adsorbing the CO_2_ on the clean CeO_2_ for which a good agreement with the literature (−0.36 eV) was found (formation of carbonate species). Figure 12D shows that the adsorption of the CO_2_ molecule on CeO_2_ (111) and Ce–La–O (111) surfaces follows the same behavior, whereas as the biaxial tensile strain (external stimulus) increases, the *E*_ads_ value drops, indicating stronger adsorption. Introduction of the dopant in the ceria lattice facilitated the adsorption significantly. A fluctuating behavior of the adsorption energy was observed, where the *E*_ads_ value varied between a decrease (more negative value) as the compressive strain decreased (−3 to −1%), to reach its strongest binding at 0% strain, and then an increase (less negative value) as the tensile strain increased. It is also important to point out that the *E*_ads_ values at zero strain were more negative with O_v_ present on the surface compared to the cases where O_v_ were absent (as can be observed in Figure 13). This is due to the enhanced CT from the surface to the CO_2_ molecule.^84^ In other words, the high basicity of the surface (CO_2_-TPD earlier studies) is indicative of the CT from the surface to the CO_2_ molecule in order for the latter to be activated; the CT occurs due to the electrons residing from the O 2p shell upon creating the O_v_.^85^ The presence of dopants weakened the M–O bond (see Table S1) and facilitated the generation of oxygen vacancies that strengthened the *E*_ads_ of CO_2_.^86^

##### 3.7.2.3 Charge Transfer

**Figure 14:**
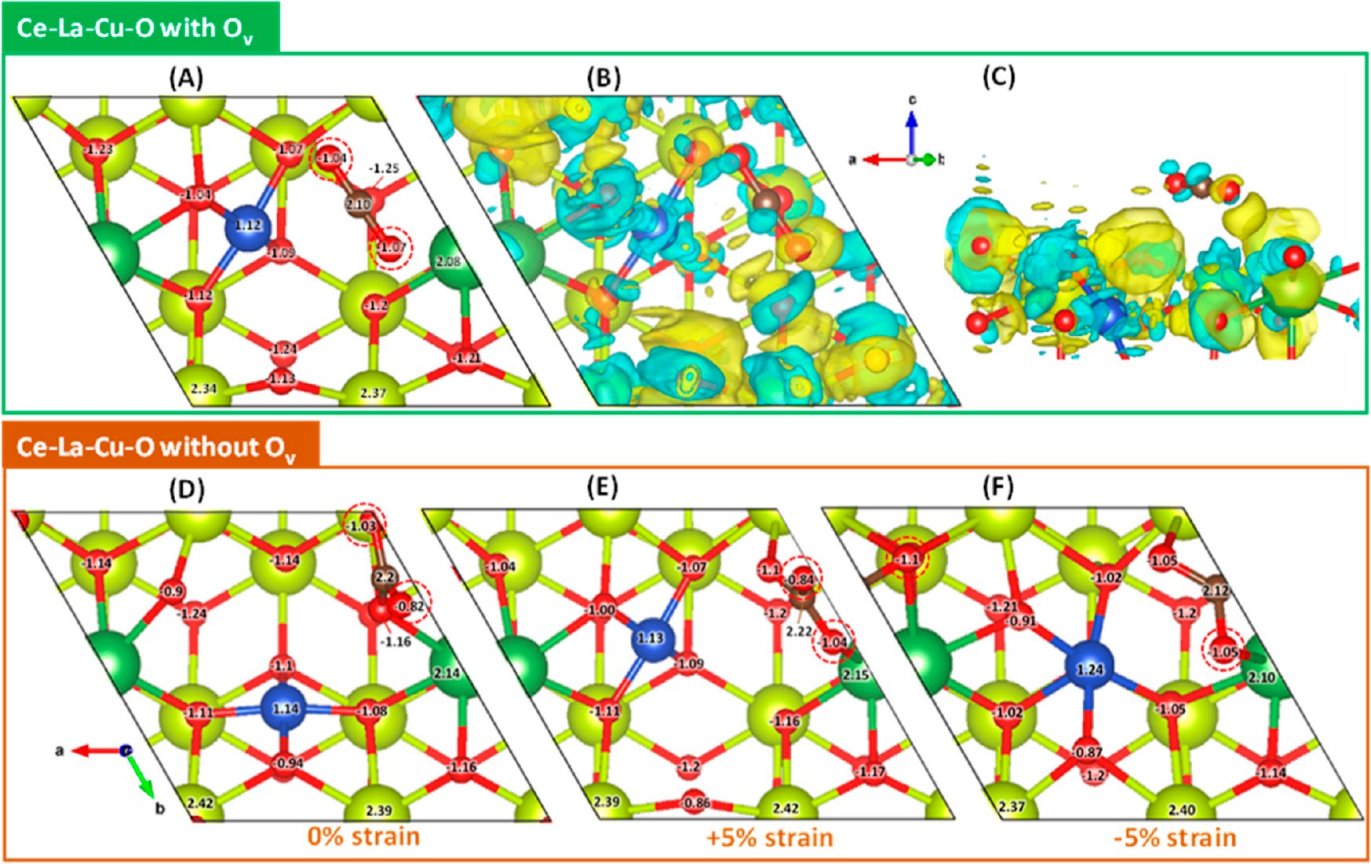
(A) Bader charge analysis, charge density difference (B) top view, and (C) side view of CO_2_ adsorbed on Ce–La–Cu–O with oxygen vacancy at site 2 at zero strain. Bader charge analysis with the strain effect on CO_2_ adsorbed on Ce–La–Cu–O at (D) 0, (E) +5, and (F) −5% strain in the absence of the oxygen vacancy (O_v_). Yellow and blue regions in charge density difference plots denote charge accumulation and depletion, respectively. An isosurface value of 0.0025 eÅ^–3^ is used.

To gain understanding on the adsorbate/surface charge distribution upon CO_2_ adsorption on the best performing surface of doped CeO_2_(111) with lanthanum and copper, Bader charge analysis along with charge density difference has been performed. Results of the Ce–La–Cu–O surface considering first the effect of oxygen vacancies at zero strain followed by the Ce–La–Cu–O surface at different levels of strain in the absence of oxygen vacancies are shown in Figure 14.

*Effect of oxygen vacancy:* The effect of oxygen vacancy (O_v_) on the CO_2_-derived carbon atom (C^δ+^) and the CO_2_-derived oxygen atoms (O^δ−^) and charge changes on the surface are discussed next. Upon CO_2_ adsorption on the Ce–La–Cu–O surface and in the presence of O_v_, the charge associated with the CO_2_-derived carbon atom was found to be +2.10 |e^−^| (Figure 14A), whereas in the absence of O_v_ was calculated to be +2.20 |e| (Figure 14D), both being compared to the 0% strain condition. Thus, the presence of O_v_ enhances the electron accumulation on the carbon atom (C^δ+^) as its value is found to be less positive. In Figure 14B,C, the charge accumulation region on the C atom is designated with the blue region.

In the case of CO_2_-derived oxygen atoms, one of the oxygen atoms (O_1_) shows similar values (−1.04 and −1.03 |e|) in both cases, whereas the second oxygen atom (O_2_) had distinct charges of −1.07 and −0.82 |e| in the case of the Ce–La–Cu–O surface with and without oxygen vacancy, respectively. Therefore, the O_2_ atom in the adsorbed CO_2_ had higher charge accumulation in the presence of O_v_; hence, in the presence of O_v_, a stronger electric field is formed due to the excess electrons surrounding the oxygen atoms belonging to CO_2_. Additionally, the charge density difference plot reveals the region of electron accumulation around the O_2_ in the CO_2_ molecule (Figure 14B,C), anticipating the participation of this oxygen atom in charge-exchange events with the surface (surface acting as the electron donor).

*Effect of applied strain:* Comparing the tensile (+5%) and compressive (−5%) strain to the zero strain condition in the case of Ce–La–Cu–O, it can be seen that +5% strain has a negligible effect on the CO_2_ electron transfer toward/from the surface based on similar values of charges on the adsorbed carbon and oxygen atoms (2.22 for carbon, −1.04 and −0.84 |e| for both oxygens) compared to the 0% strain case (2.22 for carbon and −1.03 and −0.87 |e| for both oxygens). In the above reported values, the O atoms with the low charge value are the ones pulled upward along the *z* direction. However, upon applying compressive strain (−5%), both oxygens are pulled toward the surface having more negative charge than in the 0 and +5% strain cases.

The CO_2_-derived C atom (C^δ+^) is also more enriched with electrons having a charge of 2.12 |e| (Figure 14F) compared to that of 2.22 |e| (Figure 14D,E). Thus, these charges under −5% applied strain suggest the formation of covalent bonds within the CO_2_/La–Ce–Cu–O system, thus indicating strong CO_2_ adsorption.

#### 3.7.3 Electronic DOS*:* Influence of Chemical Strain (ε_C_) and Mechanical Strain (ε_M_) on the Electronic Structure of Doped CeO_2_(111) Surfaces

**Figure 15:**
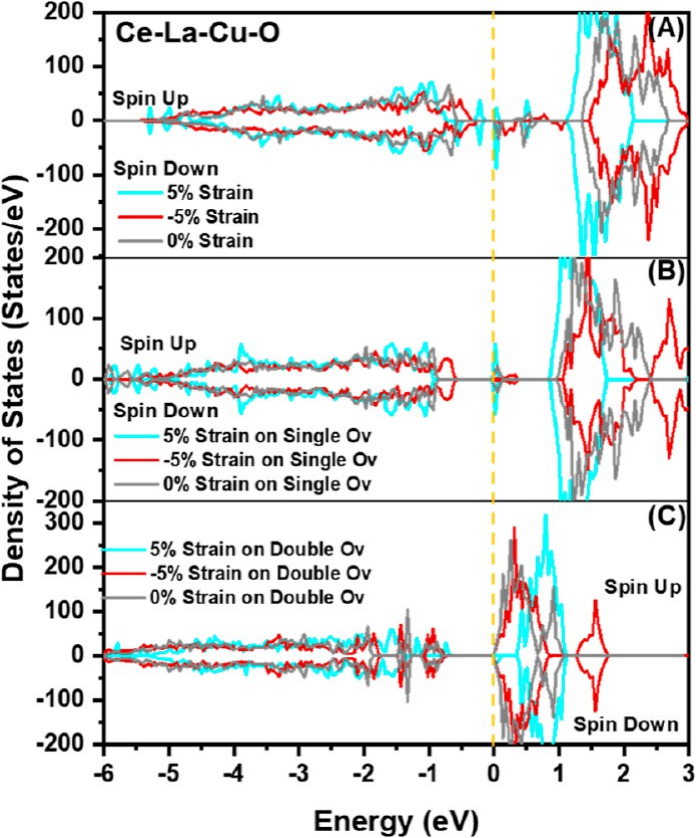
DOS of Ce–La–Cu–O(111) under −5, 0, and +5% strain level with different configurations of (A) clean surface, (B) reduced surface, single oxygen vacancies, and (C) reduced surface, double oxygen vacancies. The dashed vertical line represents the Fermi level.

In this section, the role of O_v_ as well as that of tensile and compressive strain on the DOS of the pristine and doped (La)/co-doped (La, Cu) ceria is discussed. The relative position of the CB and VB is important when adsorbent–adsorbate interactions have a covalent characteristic. In such a case, strain can alter the energetic positions of the bands and thereby the adsorption properties. Furthermore, covalent interaction as that dictated by the VB–CB splitting means higher dependence of basicity from the strain.^87^ The calculated DOS for clean and defected—single and double oxygen vacancies—CeO_2_(111), Ce–La–O(111), and Ce–La–Cu–O(111) are given in Figures 15S13, and S14.

##### 3.7.3.1 CeO_2_(111) Surface

The main contribution to the CeO_2_(111) valence band (VB) and conduction band (CB) configurations is expected to originate from the O 2p and Ce 5d orbitals allocated below and above the Fermi level, respectively. The Fermi level is represented by a dashed vertical line in all DOS plots. It is important to note that in most cases, the value of the band gap (*E*_g_) calculated using DFT is underestimated, mainly due to the exchange–correlation derivative discontinuity.^88^

In the case of clean CeO_2_(111) (absence of oxygen vacancies), no gap states were observed in the wide band gap region between the VB and CB (O 2p and Ce 5d).^89,90^ Such a DOS profile is characteristic of an insulator with empty f-orbitals, the case of no surface defect formation.

However, in the reduced CeO_2_ case, it is noted that the presence of both single O_v_ (Figure S13B) and double O_v_ (Figure S13C) results in an observable reduction of the O_2p_–Ce_5d_ band gap. This may be due to the fact that oxygen vacancies increase the number of readily available excess electrons in both surface and subsurface layers. This in turns induces charge compensation by Ce atoms to minimize the bonds between neighboring oxygen atoms that ultimately result in the smaller O_2p_–Ce_5d_ gap. Prominently, in the case of reduced ceria surfaces with single and double oxygen vacancies, the −5% applied strain reveals the greatest reduction in band gap energy. Besides, a high peak of spin-up defected state at around 0.4 eV is witnessed with −5% applied compression for surfaces with single oxygen vacancy, while in the cases of double oxygen vacancies, the highest intensity of 0% strain is observed in the band gap region.

##### 3.7.3.2 Doped CeO_2_(111) Surfaces

To provide an in-depth understanding of the effect of lanthanum and copper doping (chemical strain) on the electronic structure of ceria coupled with the mechanical strain (induced by the imposed biaxial strain), the DOS of doped CeO_2_(111) along with surface defects and imposed biaxial mechanical strain applied are presented for La-doped-CeO_2_ (Figure S14) and for La, Cu co-doped CeO_2_ (Figure 15). First, it can be seen that the presence of La and Cu dopants causes more complex splitting of the Cu 3d states and La 4f states formed from the effect of the dopant compared to that of pure CeO_2_ surface (Figure S14).

##### 3.7.3.3 Ce–La–O(111) Surface

In this case, coupling of both lattice strain (O_v_) and mechanical biaxial strain effects leads to the following noteworthy results: (a) −5% compression strain has been observed to result in the lowest band gap for both cases of reduced Ce–La–O(111) surface; single and double oxygen vacancies (Figure S14B,C green curve). Moreover, the single and double oxygen vacancies in the Ce–La–O-doped surface incurred splitting in the suspected Ce 4f- and La 4f-related states, with greatest splitting intensities obtained for the case of −5% for single O_v_ and 0% for double O_v_ (Figure S14B,C). These mixed-occupied Ce 4f and La 4f states within the band gap are interpreted as an increased CT efficiency of the surface, a factor usually promoting surface activity in catalytic reactions.^91^

##### 3.7.3.4 Ce–La–Cu–O(111) Doped Surfaces

In this case, it is observed that the CB shifts toward the VB (or closer to the Fermi level) as we move from the clean surface to the reduced surface with one O_v_ to the reduced surface with 2 O_v_. The shift is significant in the case of the reduced Ce–La–CuO(111) surface with 2 O_v_. Therefore, among the Ce–La–Cu–O configurations studied, the incorporation of double O_v_ results in the smallest band gap (Figure 15C). This smallest band gap could be the result of Ce 4f with Cu 3d- and La 4f-related states formed via the Ce–La–Cu–O doping along with the promoted double vacancies. Such impurity states are expanded in order to connect with the O 2p band, which narrows the band gap width in the DOS of ceria, promoting enhanced oxygen mobility and leading to a higher catalytic activity.^91^ Upon applying compressive strain in the Ce–La–Cu–O reduced surface (Figure 15C, red curve), the CB band shift to the Fermi level is the most pronounced compared to the zero strain and tensile strain conditions, causing the highest reduction in band gap. These results are consistent with the above energy of oxygen vacancy formation (*E*_O_v__^f^) in which the reduction in the band gap found after ceria surface doping is consistent with the decrease of *E*_O_v__^f^ for Ce–La–O and Ce–La–Cu–O CeO_2_(111) surfaces. From the projected DOS calculations (Figure S15) performed at 0% strain, only for the case of Ce–La–Cu–O oxide of interest, it was found that Cu 3d-induced impurity states with new peaks extended from the O 2p band toward the Fermi level, which narrows the band gap in total DOS (Figure 15).

### 3.8 On the Strain Tuning of the CO_2_ Activation over the Ce–La–Cu–O Surface through the DFT and Experimental Tools

#### 3.8.1 Metal Oxide Role As Acid/Base

A metal oxide can behave as a Lewis acid (electron acceptor); in such a case, the M^*n*+^ sites interact with the CO_2_-derived oxygen forming M^*n*+^–O_CO_2__ entities. In the case where the metal oxide acts as a Lewis base (electron donor), the O^2–^_surface_ site interacts with the CO_2_-derived carbon atom forming O^2–^_surface_–C_CO_2__ entities.^13^

CO_2_ can react with a metal oxide acid/base surface toward the formation of carbonate or bicarbonate species through the participation of O_surface_ or OH_surface_, respectively, as well as linear adsorbed species, the latter being perpendicular or parallel to the surface. It has been reported that the surface with acidic characteristic promotes the linear-type adsorbed CO_2_ species, whereas basic characteristic promotes the bent configurations (e.g., CO_2_^–^ or HCO_3_^–^ species). During the CO_2_^–^ species (bent configuration) formation, electron transfer to the π antibonding orbitals of CO_2_ takes place.^13^

#### 3.8.2 CO_2_–Ce–La–Cu–O(111) Interaction and Surface-Bound Species Geometry

According to the literature,^92^ the CO_2_ activation depends on the O_v_ and the CT; the latter can be probed by the O–C–O angle. The most stable configurations of CO_2_ into contact with the relaxed structures of CeO_2_(111), Ce–La–O(111), and Ce–La–Cu–O(111) were studied, and the results are presented in Figures 12 and 13. Adsorption of CO_2_ onto the surface leads to the formation of a CO_2_ complex, identified as carbonates; in the latter complex entity, two of the C–O bonds are elongated and the third one corresponds to the C (coming from CO_2_) and the O from the surface (O_surf_) (see Tables 4, 5, and S19–S21).

**Table 5:** Activated CO_2_ Molecule on CeO_2_(111), Ce–La–O(111), and Ce–La–Cu–O(111); Bond Length (Å) and O–C–O Angle (°) in the Absence of Oxygen Vacancies (O_v_) and at the 0, 5, and −5% Strain Level

strain (%)	5%	0%		–5%		
oxide system	O–C–O (deg)	O=C (Å)	O–C–O (deg)	O=C (Å)	O–C–O (deg)	O=C (Å)
CeO_2_(111)	126.78	1.27/1.27	130.32	1.26/1.26	178.78	1.17/1.17
Ce–La–O(111)	119.96	1.28/1.28	125.93	1.26/1.27	123.64	1.26/1.25
Ce–La–Cu–O(111)	119.88	1.28/1.27	120.04	1.27/1.27	124.75	1.27/1.27

The O–C–O angle of the adsorbed CO_2_ molecule is in the range of 125–128°, while the two O_surf_–C–O angles involving the surface oxygen are in the range of 114–121°. The deviation of the O–C–O angle from the linear geometry of the CO_2_ gas phase, 180°, demonstrates CO_2_ activation.^92^ Moreover, the difference of the O_surf_–C–O_1_ and O_surf_–C–O_2_ angles denotes the different extent of bonding of the O_1_ and O_2_ of CO_2_ with the surface. This is in agreement with the Bader CT as discussed later. In the adsorbed state of CO_2_, the C–O bonds present distances between 1.26 and 1.28 Å with the C–O_surf_ bond length being in the 1.35–1.39 Å range. Compared to the gas phase (C–O ≈ 1.16 Å), it can be concluded that C–O bonds in the adsorbed CO_2_ state are elongated to a great extent with a significant degree of O–C–O bond bending, as discussed earlier. These findings corroborate for activated chemisorbed CO_2_ species. The significant reduction of the O–C–O angle upon adsorption is mirrored to the reduction of the adsorption energy (*E*_ads_).^93^

#### 3.8.3 *E*_ads_ and O–C–O Angle as Descriptors of Activation

Doping ceria and accounting for the presence of O_v_ (Figure 12) (more realistic scenario) leads to a significant *E*_ads_*value reduction* as we move from CeO_2_, to Ce–La–O and Ce–La–Cu–O surface (−1.40 eV). This is accompanied by a significant reduction of the O–C–O angle value (124.71°) for the Ce–La–Cu–O surface and a C–O_surf_ (Å) distance of 1.34 Å. This implies a stronger CO_2_-surface CT in this case. Doping effect in the *absence of* O_v_ (Figure 13) causes similar impact on the *E*_ads_ at zero, +5, and −5% level of the imposed strain. However, the impact is more significant (higher drop of *E*_ads_ as we move from CeO_2_ to Ce–La–O oxide). The addition of the second dopant, Cu, has minor impact in this parameter.

##### 3.8.3.1 Strain Effects

For the Ce–La–Cu–O case, high level of compressive strain does not favor the CO_2_–surface interaction, as it is supported by the O_surf_–C_CO_2__ distances reported in Tables S19–S21 (not bound species formed). On the other hand, the tensile strain leads to favorable CO_2_ interaction with the surface of interest. In the case of CeO_2_ and Ce–La–O surfaces, as we move from the compressive field to a tensile one, *E*_ads_ drops corroborating for a stronger interaction as well.

#### 3.8.4 CO_2_–Surface Electronic Interaction

Bader charge analysis was performed only in the case of Ce–La–Cu–O(111) surface, at site 2 (Cu–O_v_–La) following two scenarios, namely, the presence or absence of O_v_. The *presence of* O_v_ was found to favor the electron accumulation on the CO_2_-derived carbon (**C**_CO_2__) as well as the CO_2_-derived oxygen atoms (O_CO_2__), corroborating for the dual role of the Ce–La–Cu–O surface participating with both basic (**O**^**2–**^_**surface**_**–C**_**CO**_**2**__) and acidic (**M**^***n*+**^**–O**_**CO**_**2**__) sites in its interaction with the CO_2_ molecule. Having a closer look on the effect of strain on the CO_2_–surface interaction, it can be seen that tensile strain leads to the same charge distribution for the **C**_CO_2__ and the **O**_CO_2__ atoms. However, it seems that compressive strain enhances the basic characteristic of the surface, as **C**_CO_2__ is more electron enriched, allowing us to speculate for more participation of the surface basic sites in the interaction with the CO_2_ and thus for a possible surface acidity–basicity tuning upon strain imposed. The above are also supported from the data listed in Table 5, where both under compressive (−5%) and tensile (5%) strain level, the O–C–O angle and the C=O bond are distorted. The lower value of the O–C–O angle under tensile (119.88°) compared to the same angle value under compressive (124.75°) allows us to discuss the strength of the interaction (strength of acid/basic sites). This is supported by the DOS (Figure 15B) of Ce–La–Cu–O(111) reduced surface, where in both tensile and compressive fields, the formation of new gap states can be noticed, leading to an apparent narrowing of the band gap (VB–CB splitting). The latter has been reported to have an instrumental role in the strain tuning of surface basicity, when covalent bonding is involved.^87^ The new gap states have different shapes and width implying different electron distributions on the surface upon different types of strain imposed on the surface. More focused studies using near-ambient pressure XPS and extended X-ray absorption fine structure can enlighten more the role of strain on the acid/base characteristic of the surface.^94,95^

Based on the present experimental findings of in situ DRIFTS studies, the bidentate carbonates were found as predominant species on the pristine (chemically strained) and DBM (chemically and mechanically strained) surfaces, justifying the dual-site interaction of CO_2_ with the surface.^80^ Addition of mechanical strain led to an increase in the population of bidentate carbonate species. It has been reported that the free carbonate ion (*D*_3*h*_ symmetry) has an IR band at 1415 cm^–1^. Upon adsorption, lowering of the symmetry gives rise to two ν(CO) bands on both sides of the band at 1415 cm^–1^. The separation between the two bands is known as Δν_3_ splitting,^96^ which is used as a descriptor of the surface basic strength. Usually, a smaller splitting accounts for stronger basic sites; for the different carbonate species, different values are reported; unidentate (Δν_3_ = 100 cm^–1^), bidentate (Δν_3_ = 300 cm^–1^), and bridged species (Δν_3_ = 400 cm^–1^). The Δν_3_ splitting in Figure 9 is 268 cm^–1^, characteristic of bidentate species formed in moderate-strength basic sites. Bidentate carbonates are favored over the medium-strength basic sites (see Figure 8); the latter are present in the Ce–La–Cu–O surface and tuned upon mechanochemical processing (ball milling) (see Figure 8B). The pristine and the DBM solids is another example of isostructural oxides with a different behavior upon strain. The latter is demonstrated not only by the different population of the carbonates formed but also from their different thermal stabilities. The different thermal stability reflects the strength of interaction and is justified by the different O_surf_–C–O_1_ and O_surf_–C–O_2_ angles (deformation) in zero and 5% strain levels.

### 3.9 Case Study: DRM Reaction

In order to validate the effect of mechanochemistry concepts in catalysis, the DRM reaction was selected as a probe one. The supported Ni catalysts were prepared based on the procedure mentioned earlier (Section 2.3), where the pristine, dry ball-milled, and wet ball-milled Ce–La–Cu–O oxides were used as supports.

**Figure 16:**
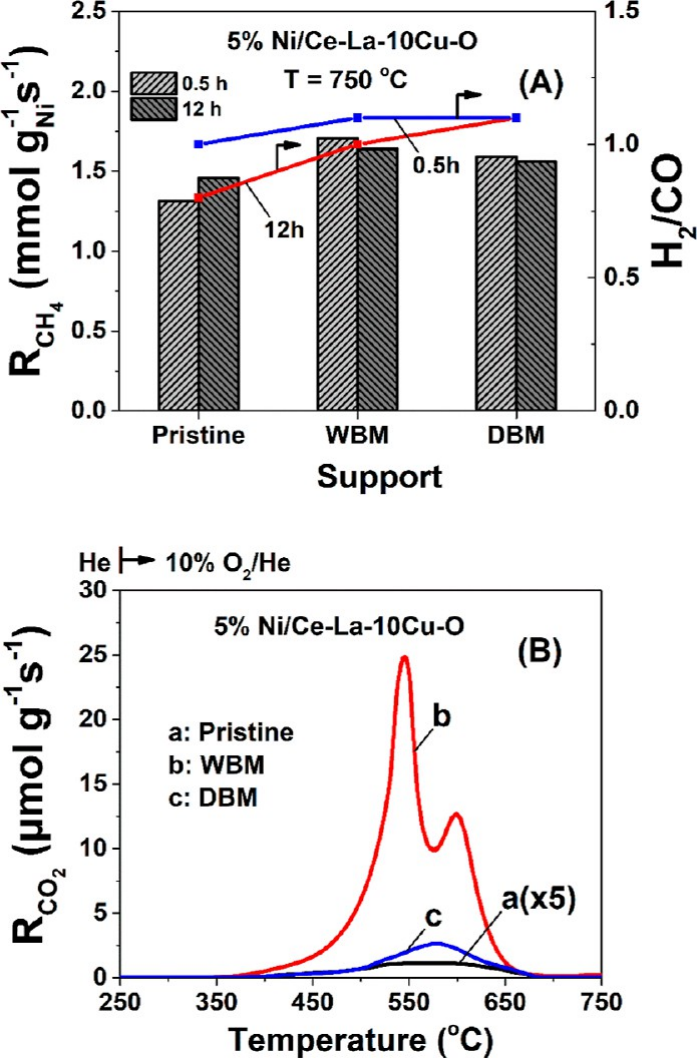
(A) Integral rate of CH_4_ conversion (mmol g^–1^_Ni_ s^–1^) and H_2_/CO gas product ratio obtained after 0.5 and 12 h of DRM (20 vol % CH_4_/ 20% CO_2_/He) at 750 °C over 5 wt % Ni/Ce–La–10Cu–O catalysts as a function of support pre-treatment (pristine, WBM, and DBM). (B) Transient response curves of CO_2_ formation rate (μmol g^–1^ s^–1^) obtained during TPO of carbon formed after 12 h of DRM (20 vol % CH_4_/20 vol % CO_2_/He) at 750 °C over 5 wt % Ni/Ce–La–10Cu–O catalysts: (a) pristine, (b) WBM, and (c) DBM.

Figure 16A and Table 6 present the experimental CH_4_ and CO_2_ integral rate values after 0.5 and 12 h of DRM reaction, as well as the amount of carbon accumulated on the catalyst surface after 12 h of DRM. In the case of the pristine-supported Ni catalyst, the conversions were lower than the equilibrium values when DRM is only considered but also for the DRM/reverse water gas shift (RWGS) reaction network (see Table 6). In our previous work,^27^ it was found (use of transient isotopic experiments) that the decrease of carbon deposition in the pristine-supported Ni catalyst was due to carbon oxidation (or gasification) to CO by labile lattice oxygen, where CO_2_ reoxidizes the reduced ceria-based support. In the case of all the catalysts listed in Table 6, for short time-on-stream (TOS) (ca. 0.5 h), the H_2_/CO ratio adopts a value slightly higher than unity, signifying the simultaneous presence of some side reactions, such as the RWGS, CH_4_ decomposition, carbon oxidation to CO and CO_2_, and steam methane reforming. The RWGS explains the drop in the H_2_/CO gas ratio, the increase of *X*_CO_2__, and the drop in the H_2_-yield after 12 h TOS, as shown in Table 6. Overall, DBM and WBM have increased the CH_4_ and CO_2_ conversions for TOS of 0.5 and 12 h to a different extent. This is linked to the modification of the Ni–support interface upon the milling process, as demonstrated through H_2_-TPD experiments performed over the three supported Ni catalysts (pristine, dry ball milled, wet ball milled; Figure S16). In the H_2_-TPD profiles of the milled catalysts (both DBM and WBM), low-temperature desorption peaks appeared, which is not the case for the pristine-supported Ni catalyst (Figure S16 and Table S22), linked with the presence of smaller size Ni crystallites grown on the milled supports. Ball milling was also found to alter the redox properties of the catalysts along with the distribution of labile oxygen species present on the surface and in the subsurface region; the latter can be inferred by the shape of the peaks (Figure S17). Additionally, DBM seems to have an opposite effect on the catalyst reducibility compared to the DBM (Table S23).

**Table 6:** Conversions of CH_4_ and CO_2_, H_2_-Yield (%), and H_2_/CO Gas Product Ratio after DRM at 750 °C (20% CO_2_/20% CH_4_/He) for 0.5 and 12 h over the 5 wt % Ni Supported on Ce–La–10Cu–O Carriers (Pristine, WBM, and DBM)^a^

catalyst 5 wt % Ni/Ce–La–10Cu–O	time (h)	*X*_CH_4__ (%)	*X*_CO_2__ (%)	H_2_-yield (%)	H_2_/CO	mg C g_cat_^–1^
pristine	0.5	80.3 (86.6)^b^ (83.6)^c^	76.4 (86.6)^b^ (90.3)^c^	55.3	1.05	
	12	80.8	81.4	42.7	0.8	1.5
WBM	0.5	91.5	93.5	71.7	1.1	
	12	88.7	91.7	57.9	1	70.7
DBM	0.5	86.8	86.1	53.33	1.1	
	12	85.1	85.2	53.9	1.1	7.7

a The amount of carbon accumulated (mg C g^–1^_cat_) after 12 h of DRM is also presented.

b Number in parentheses gives the equilibrium conversions of CH_4_ and CO_2_ and the H_2_/CO product gas ratio for the DRM reaction alone; feed gas composition (20% CO_2_/20% CH_4_/He); 750 °C.

c Number in parentheses gives the equilibrium conversions of CH_4_ and CO_2_ and the H_2_/CO product gas ratio when both the DRM and RWGS reactions participate in the reaction network; feed gas composition (20% CO_2_/20% CH_4_/He); *T* = 750 °C.

The carbon accumulated on the catalyst surfaces was measured by temperature-programmed oxidation (TPO) (see Figure 16B). It is also noteworthy to mention the two prominent peaks that appear in the TPO-CO_2_ trace of Ni/Ce–La–10Cu–O catalyst, ca. ∼550 and 600 °C (Figure 16B), indicating the formation of two types of carbon. This is in agreement with our previous findings on Ni/Ce–La–Cu–O-supported catalysts (pristine),^27^ where the La-doped catalyst resulted in an asymmetric (i.e., two peaks) TPO-CO_2_ trace at ∼550 °C, indicating the formation of two different types of carbon. These peaks became more prominent and well defined after WBM (curve b).

Based on the above results, it can be suggested that ball milling could benefit several catalytic reactions involving ceria, such as chemical looping water splitting (CLWS) coupled with the decomposition of glycerol,^97^ aqueous-phase reforming (APR) of glycerol,^98^ and chemical looping steam reforming of glycerol,^99^ where ball milling could induce the tuning of CO_2_ (as product this time) interaction with the CeO_2_-related surface toward the preferred direction. In the study reported by Dou et al.,^97^ CeO_2_ acts as a promoter, attributing to stronger metal–support interactions and enhancing the thermal stability of the Ni–CeO_2_/MCM-41 or SBA-15 catalysts. In the work of Wu et al.,^98^ the Ni–Cu bimetallic supported on mesoporous CeO_2_ was used for APR of glycerol (biodiesel byproduct). The kinetic analysis conducted proved that after adding CaO onto the 1Ni2Cu/CeO_2_ catalyst, the apparent activation energy dropped, ca. 29.86 kJ mol^–1^. Here, CaO is integrated as an absorbent to facilitate the WGS reaction and reduce methanation reactions via in situ CO_2_ removal and capture. Last but not least, Lou et al. reported on Fe–Ce–Ni–O-based oxygen carriers (OCs) for CLWS coupled with glycerol decomposition in an attempt to simultaneously produce hydrogen and syngas.^99^ A remarkable redox behavior was achieved by the prepared Fe–Ce–Ni–O-based OCs, leading to a significant catalytic function of partial oxidation and decomposition of glycerol at 750 °C. The best oxygen-transfer capability and highest hydrogen and syngas production were attained by OC with a 100:10:3 molar ratio of Fe/Ce/Ni.

## 4 Conclusions

In this work, we presented the first example of how the chemical (ε_C_) and mechanical (ε_M_) strain can be used to tailor the CO_2_ activation and adsorption onto a metal oxide surface by a combined experimental modeling approach. To demonstrate this, the Ce–La–Cu–O ternary oxide surface was prepared using a microwave coupled with sol–gel and then subjected into mechanochemical treatment (ball milling). Chemical strain is originated from the CeO_2_ lattice doping (La^3+^ or La^3+^/Cu^2+^), whereas mechanical strain is originated from post-synthetic ball milling treatment. This study reveals the impact of the mechanical strain (ball milling) on the *mobility of lattice oxygen* (O_L_) through state-of-the-art ^16^O/^18^O transient isotopic exchange experiments. Strained (tensile) Ce–La–Cu–O surfaces were found to facilitate the O_v_ formation and the CO_2_ adsorption. The interplay between the O_v_ entities and the CO_2_ activation and the role of mechanical strain were illustrated through in situ DRIFTS studies and Bader charge analysis. In particular, the DBM process (mechanical strain) was found to enhance by 6 times the population of the bidentate carbonates formed, though lowering their thermal stability. Geometric characteristics of the adsorbed CO_2_ species were used to evaluate the molecule deformation and the strength of adsorption, such as C–O and O_surf_–C bond lengths (Å), as well as O–C–O and O_surf_–C–O_1_ and O_surf_–C–O_2_ angles, demonstrating how the mechanical strain can assist an already chemically strained surface in its interaction with the CO_2_. Based on the in situ DRIFTS studies along with the O_surf_–C–O_1_ and O_surf_–C–O_2_ angles and the Bader charge analysis, a dual role of the Ce–La–Cu–O surface is proposed, through which the surface utilizes both its acidic and basic sites; the relative contribution of the sites in the interaction with CO_2_ can be tuned by the mechanical strain applied. Having the possibility to tune the participation of the acidic or basic sites in the interaction with CO_2_, this leads to carbonates with different stabilities; the latter is of high importance in catalytic reactions, such as DRM and CO_2_ hydrogenation, where the CO_2_ activation and carbonate formation as active intermediates are important mechanistic steps. A proof of concept is herein demonstrated using the DRM reaction, where Ni-based catalysts supported on a ball-milled carrier exhibited different (smaller) Ni crystallite sizes and enhanced labile oxygen species features that led to higher CO_2_ and CH_4_ conversions and significantly lower carbon deposition.

## Supporting Information Available

Characterization details such as XRD and Raman of the reference materials, HRTEM over the fresh Ce–La–10Cu–O oxide, textural studies, H_2_-TPR of the fresh and reference oxides, CO_2_-TPD of the reference oxides, XPS over the Ce–La–10Cu–O oxide before and after the ball milling, and IR spectra of Ce–La–10Cu–O—pristine oxide; some of the computational (DFT) data; DOS, analytical data of the energies of formation of oxygen vacancies at different positions, and different arrangements and different levels of strain; geometric characteristics of the adsorbed CO_2_ species over the reference oxides; and dispersion of the Ni-supported catalysts as well as their H_2_-TPR and H_2_-TPD (PDF)

## References

[ref1] Gsell M.; Jakob P.; Menzel D. Effect of substrate strain on adsorption. Science 1998, 280, 717 10.1126/science.280.5364.717. 9563943

[ref2] Mavrikakis M.; Hammer B.; Nørskov J. K. Effect of strain on the reactivity of metal surfaces. Phys. Rev. Lett. 1998, 81, 2819 10.1103/physrevlett.81.2819.

[ref3] Šepelák V.; Düvel A.; Wilkening M.; Becker K. D.; Heitjans P. Mechanochemical reactions and syntheses of oxides. Chem. Soc. Rev. 2013, 42, 7507 10.1039/c2cs35462d. 23364473

[ref4] Sasikumar C.; Srikanth S.; Kumar R.; Alex T. C.; Mehrotra S. P. Where Does the Energy Go in High Energy Milling. Frontiers in Mechanochemistry and Mechanical Alloying; CSIR-National Metallurgical Laboratory: Jamshedpur-831007, India, 2011.

[ref5] Suryanarayana C. Mechanical alloying and milling. Prog. Mater. Sci. 2001, 46, 1–184. 10.1016/s0079-6425(99)00010-9.

[ref6] Laguna O. H.; Pérez A.; Centeno M. A.; Odriozola J. A. Synergy between gold and oxygen vacancies in gold supported on Zr-doped ceria catalysts for the CO oxidation. Appl. Catal., B 2015, 176–177, 385 10.1016/j.apcatb.2015.04.019.

[ref7] Han W.-Q.; Wu L.; Zhu Y. Formation and Oxidation State of CeO2-x Nanotubes. J. Am. Chem. Soc. 2005, 127, 12814 10.1021/ja054533p. 16159271

[ref8] Liu X.; Zhou K.; Wang L.; Wang B.; Li Y. Oxygen Vacancy Clusters Promoting Reducibility and Activity of Ceria Nanorods. J. Am. Chem. Soc. 2009, 131, 3140 10.1021/ja808433d. 19215075

[ref9] Pastor-Pérez L.; Ramos-Fernández E. V.; Sepúlveda-Escribano A. Effect of the CeO_2_ synthesis method on the behaviour of Pt/CeO_2_ catalysis for the water-gas shift reaction. Int. J. Hydrogen Energy 2019, 44, 21837–21846. 10.1016/j.ijhydene.2019.06.206.

[ref10] Baumann N.; Lan J.; Iannuzzi M. CO_2_ adsorption on the pristine and reduced CeO_2_ (111) surface: Geometries and vibrational spectra by first principles simulations. J. Chem. Phys. 2021, 154, 094702 10.1063/5.0042435. 33685147

[ref11] Hahn K. R.; Iannuzzi M.; Seitsonen A. P.; Hutter J. Coverage effect of the CO_2_ adsorption mechanisms on CeO_2_(111) by first principles analysis. J. Phys. Chem. C 2013, 117, 1701 10.1021/jp309565u.

[ref12] Mutch G. A.; Shulda S.; McCue A. J.; Menart M. J.; Ciobanu C. V.; Ngo C.; Anderson J. A.; Richards R. M.; Vega-Maza D. Carbon Capture by Metal Oxides: Unleashing the Potential of the (111) Facet. J. Am. Chem. Soc. 2018, 140, 4736 10.1021/jacs.8b01845. 29553264

[ref13] Álvarez A.; Borges M.; Corral-Pérez J. J.; Olcina J. G.; Hu L.; Cornu D.; Huang R.; Stoian D.; Urakawa A. CO_2_ Activation over Catalytic Surfaces. ChemPhysChem 2017, 18, 3135 10.1002/cphc.201700782. 28851111

[ref14] Tsipouriari V. A.; Verykios X. E. Carbon and Oxygen Reaction Pathways of CO_2_ Reforming of Methane over Ni/La_2_O_3_ and Ni/Al_2_O_3_ Catalysts Studied by Isotopic Tracing Techniques. J. Catal. 1999, 187, 85–94. 10.1006/jcat.1999.2565.

[ref15] Das S.; Sengupta M.; Patel J.; Bordoloi A. A study of the synergy between support surface properties and catalyst deactivation for CO_2_ reforming over supported Ni nanoparticles. Appl. Catal., A 2017, 545, 113 10.1016/j.apcata.2017.07.044.

[ref16] Mogensen M.; Sammes N. M.; Tompsett G. A. Physical, chemical and electrochemical properties of pure and doped ceria. Solid State Ionics 2000, 129, 63 10.1016/S0167-2738(99)00318-5.

[ref17] Schwarz K. Materials design of solid electrolytes. Proc. Natl. Acad. Sci. U.S.A. 2006, 103, 3497 10.1073/pnas.0600327103. 16505351 PMC1533771

[ref18] Alketbi M.; Polychronopoulou K.; Abi Jaoude M.; Vasiliades M. A.; Sebastian V.; Hinder S. J.; Baker M. A.; Zedan A. F.; Efstathiou A. M. Cu-Ce-La-Ox as efficient CO oxidation catalysts: Effect of Cu content. Appl. Surf. Sci. 2019, 505, 144474 10.1016/j.apsusc.2019.144474.

[ref19] Huygh S.; Bogaerts A.; Neyts E. C. How Oxygen Vacancies Activate CO_2_ Dissociation on TiO_2_ Anatase (001). J. Phys. Chem. C 2016, 120, 21659 10.1021/acs.jpcc.6b07459.

[ref20] Kasatkin I.; Kurr P.; Kniep B.; Trunschke A.; Schlögl R. Role of Lattice Strain and Defects in Copper Particles on the Activity of Cu/ZnO/Al_2_O_3_ Catalysts for Methanol Synthesis. Angew. Chem., Int. Ed. 2007, 46, 7324 10.1002/anie.200702600. 17868182

[ref21] Amrute A. P.; De Bellis J.; Felderhoff M.; Schüth F. Mechanochemical Synthesis of Catalytic Materials. Chem.—Eur. J. 2021, 27, 6819–6847. 10.1002/chem.202004583. 33427335 PMC8248068

[ref22] Danielis M.; Colussi S.; Llorca J.; Dolan R. H.; Cavataio G.; Trovarelli A. Pd/CeO2 Catalysts Prepared by Solvent-free Mechanochemical Route for Methane Abatement in Natural Gas Fueled Vehicles. Ind. Eng. Chem. Res. 2021, 60, 6435–6445. 10.1021/acs.iecr.0c05207.

[ref23] Danielis M.; Betancourt L. E.; Orozco I.; Divins N. J.; Llorca J.; Rodríguez J. A.; Senanayake S. D.; Colussi S.; Trovarelli A. Methane Oxidation Activity and Nanoscale Characterization of Pd/CeO2 Catalysts Prepared by Dry Milling Pd Acetate and Ceria. Appl. Catal., B 2021, 282, 119567 10.1016/j.apcatb.2020.119567.

[ref24] Makagon E.; Wachtel E.; Houben L.; Cohen S. R.; Li Y.; Li J.; Frenkel A. I.; Lubomirsky I. All-Solid-State Electro-Chemo-Mechanical Actuator Operating at Room Temperature. Adv. Funct. Mater. 2020, 31, 2006712 10.1002/adfm.202006712.

[ref25] Mishuk E.; Ushakov A.; Makagon E.; Cohen S. R.; Wachtel E.; Paul T.; Tsur Y.; Shur V. Y.; Kholkin A.; Lubomirsky I. Electro-chemomechanical Contribution to Mechanical Actuation in Gd-Doped Ceria Membranes. Adv. Mater. Interfaces 2019, 6, 1801592 10.1002/admi.201801592.

[ref26] Du M.; Miao Z.; Li H.; Zhang F.; Sang Y.; Wei L.; Liu H.; Wang S. Oxygen-vacancy and phosphate coordination triggered strain engineering of vanadium oxide for high-performance aqueous zinc ion storage. Nano Energy 2021, 89, 106477 10.1016/j.nanoen.2021.106477.

[ref27] Hussien A. G. S.; Damaskinos C. M.; Dabbawala A. A.; Anjum D. H.; Vasiliades M. A.; Khaleel M. T. A.; Wehbe N.; Efstathiou A. M.; Polychronopoulou K. Elucidating the role of La3+/Sm3+ in the carbon paths of dry reforming of methane over Ni/Ce-La(Sm)-Cu-O using transient kinetics and isotopic techniques. Appl. Catal., B 2022, 304, 121015 10.1016/j.apcatb.2021.121015.

[ref28] Vasiliades M. A.; Harris D.; Stephenson H.; Boghosian S.; Efstathiou A. M. A novel analysis of transient isothermal ^18^O isotopic exchange on commercial Ce_x_Zr_1-x_O_2-δ_ – based OSC materials,. Top. Catal. 2019, 62, 219 10.1007/s11244-018-1116-x.

[ref29] Kohn W.; Sham L. J. Self-Consistent Equations Including Exchange and Correlation Effects. Phys. Rev. 1965, 140, A1133 10.1103/physrev.140.a1133.

[ref30] Hohenberg P.; Kohn W. Inhomogeneous Electron Gas. Phys. Rev. 1964, 136, B864 10.1103/physrev.136.b864.

[ref31] Kresse G.; Hafner J. Ab initiomolecular dynamics for liquid metals. Phys. Rev. B: Condens. Matter Mater. Phys. 1993, 47, 558–561. 10.1103/physrevb.47.558. 10004490

[ref32] Kresse G.; Hafner J. Ab initiomolecular dynamics for open-shell transition metals. Phys. Rev. B: Condens. Matter Mater. Phys. 1993, 48, 13115 10.1103/physrevb.48.13115. 10007687

[ref33] Kresse G.; Furthmüller J. Efficient iterative schemes forab initiototal-energy calculations using a plane-wave basis set. Phys. Rev. B: Condens. Matter Mater. Phys. 1996, 54, 11169 10.1103/physrevb.54.11169. 9984901

[ref34] Perdew J. P.; Burke K.; Ernzerhof M. Generalized Gradient Approximation Made Simple [Phys. Rev. Lett. 77, 3865 (1996)]. Phys. Rev. Lett. 1997, 78, 1396 10.1103/physrevlett.78.1396. 10062328

[ref35] Dudarev S. L.; Botton G. A.; Savrasov S. Y.; Humphreys C. J.; Sutton A. P. Electron-energy-loss spectra and the structural stability of nickel oxide: An LSDA+U study. Phys. Rev. B: Condens. Matter Mater. Phys. 1998, 57, 1505 10.1103/physrevb.57.1505.

[ref36] Dudarev S. L.; Manh D. N.; Sutton A. P. Effect of Mott-Hubbard correlations on the electronic structure and structural stability of uranium dioxide. Philos. Mag. B 1997, 75, 613 10.1080/13642819708202343.

[ref37] Nolan M.; Parker S. C.; Watson G. W. The electronic structure of oxygen vacancy defects at the low index surfaces of ceria. Surf. Sci. 2005, 595, 223 10.1016/j.susc.2005.08.015.

[ref38] Nolan M.; Grigoleit S.; Sayle D. C.; Parker S. C.; Watson G. W. Density functional theory studies of the structure and electronic structure of pure and defective low index surfaces of ceria. Surf. Sci. 2005, 576, 217 10.1016/j.susc.2004.12.016.

[ref39] Fabris S.; Vicario G.; Balducci G.; de Gironcoli S.; Baroni S. Electronic and Atomistic Structures of Clean and Reduced Ceria Surfaces. J. Phys. Chem. B 2005, 109, 22860 10.1021/jp0511698. 16853978

[ref40] Yeriskin I.; Nolan M. Effect of La doping on CO adsorption at ceria surfaces. J. Chem. Phys. 2009, 131, 244702 10.1063/1.3271910. 20059094

[ref41] Nolan M.; Watson G. W. Hole localization in Al doped silica: A DFT+U description. J. Chem. Phys. 2006, 125, 144701 10.1063/1.2354468. 17042625

[ref42] Han Z.-K.; Zhang L.; Liu M.; Ganduglia-Pirovano M. V.; Gao Y. The Structure of Oxygen Vacancies in the Near-Surface of Reduced CeO_2_ (111) Under Strain. Front. Chem. 2019, 7, 436 10.3389/fchem.2019.00436. 31275923 PMC6592146

[ref43] Kresse G.; Joubert D. From ultrasoft pseudopotentials to the projector augmented-wave method. Phys. Rev. B: Condens. Matter Mater. Phys. 1999, 59, 1758 10.1103/physrevb.59.1758.

[ref44] Loschen C.; Carrasco J.; Neyman K. M.; Illas F. First-principlesLDA+UandGGA+Ustudy of cerium oxides: Dependence on the effective U parameter. Phys. Rev. B: Condens. Matter Mater. Phys. 2007, 75, 35115 10.1103/physrevb.75.035115.

[ref45] The Materials Project. Materials Data on CeO_2_ by Materials Project, 2020.

[ref46] Momma K.; Izumi F. VESTA 3for three-dimensional visualization of crystal, volumetric and morphology data. J. Appl. Crystallogr. 2011, 44, 1272 10.1107/s0021889811038970.

[ref47] Qi W. H.; Wang M. P. Size and shape dependent lattice parameters of metallic nanoparticles. J. Nanopart. Res. 2005, 7, 51 10.1007/s11051-004-7771-9.

[ref48] Mendelovich L.; Tzehoval H.; Steinberg M. The adsorption of oxygen and nitrous oxide on platinum ceria catalyst. Appl. Surf. Sci. 1983, 17, 175 10.1016/0378-5963(83)90032-6.

[ref49] Fierro J. L. G.; Soria J.; Sanz J.; Rojo J. M. Induced changes in ceria by thermal treatments under vacuum or hydrogen. J. Solid State Chem. 1987, 66, 154 10.1016/0022-4596(87)90230-1.

[ref50] Grigoropoulou G.; Christoforidis K. C.; Louloudi M.; Deligiannakis Y. Structure-Catalytic Function Relationship of SiO_2_-Immobilized Mononuclear Cu Complexes: An EPR Study. Langmuir 2007, 23, 10407 10.1021/la700815d. 17764200

[ref51] Suranarayana C.; Norton M. G. X-ray Diffraction: A Practical Approach; Springer: New York, 1998.

[ref52] Delogu F.; Monagheddu M.; Mulas G.; Schiffini L.; Cocco G. Impact characteristics and mechanical alloying processes by ball milling: experimental evaluation and modelling outcomes. Int. J. Non-Equilib. Process. 2000, 11, 235.

[ref53] Baláž P.; Achimovičová M.; Balaz M.; Billik P.; Cherkezova-Zheleva Z.; Manuel Criado J.; Delogu F.; Dutkova E.; Gaffet E.; Gotor F.-J.; Kumar R.; Mitov I.; Rojac T.; Senna M.; Streletskii A.; Wieczorek-Ciurow K. Hallmarks of mechanochemistry: from nanoparticles to technology. Chem. Soc. Rev. 2013, 42, 7571 10.1039/c3cs35468g. 23558752

[ref54] Keramidas V. G.; White W. B. Raman spectra of oxides with the fluorite structure. J. Chem. Phys. 1973, 59, 1561 10.1063/1.1680227.

[ref55] Schilling C.; Hofmann A.; Hess C.; Ganduglia-Pirovano M. V. Raman Spectra of Polycrystalline CeO2: A Density Functional Theory Study. Phys. Chem. C 2017, 121, 20834 10.1021/acs.jpcc.7b06643.

[ref56] McBride J. R.; Hass K. C.; Poindexter B. D.; Weber W. H. Raman and x-ray studies of Ce1–xRExO2–y, where RE=La, Pr, Nd, Eu, Gd, and Tb. J. Appl. Phys. 1994, 76, 2435 10.1063/1.357593.

[ref57] Weber W. H.; Hass K. C.; McBride J. R. Raman study ofCeO2: Second-order scattering, lattice dynamics, and particle-size effects. Phys. Rev. B: Condens. Matter Mater. Phys. 1993, 48, 178 10.1103/physrevb.48.178. 10006765

[ref58] Trovarelli A. Structural and oxygen storage/release properties of CeO_2_-based solid solutions. Comments Inorg. Chem. 2006, 20, 263 10.1080/02603599908021446.

[ref59] AlKhoori A. A.; Polychronopoulou K.; Belabbes A.; Jaoude M. A.; Vega L. F.; Sebastian V.; Hinder S.; Baker M. A.; Zedan A. F. Cu, Sm co-doping effect on the CO oxidation activity of CeO2. A combined experimental and density functional study. Appl. Surf. Sci. 2020, 521, 146305 10.1016/j.apsusc.2020.146305.

[ref60] Polychronopoulou K.; Zedan A. F.; Katsiotis M. S.; Baker M. A.; AlKhoori A. A.; AlQaradawi S. Y.; Hinder S. J.; AlHassan S. Rapid microwave assisted sol-gel synthesis of CeO2 and CexSm1-xO2 nanoparticle catalysts for CO oxidation. J. Mol. Catal. A: Chem. 2017, 428, 41 10.1016/j.molcata.2016.11.039.

[ref61] Polychronopoulou K.; AlKhoori A. A.; Efstathiou A. M.; Jaoude M. A.; Damaskinos C. M.; Baker M. A.; Almutawa A.; Anjum D. H.; Vasiliades M. A.; Belabbes A.; Vega L. F.; Zedan A. F.; Hinder S. J. Design Aspects of Doped CeO2 for Low-Temperature Catalytic CO Oxidation: Transient Kinetics and DFT Approach. ACS Appl. Mater. Interfaces 2021, 13, 22391 10.1021/acsami.1c02934. 33834768 PMC8153538

[ref62] Abrashev M. V.; Todorov N. D.; Geshev J. Raman spectra of R_2_O_3_ (R-rare-earth) sesquioxides with C-type bixbyite crystal structure: a comparative study. J. Appl. Phys. 2014, 116, 103508 10.1063/1.4894775.

[ref63] Fernández-García M.; Martínez-Arias A.; Iglesias-Juez A.; Belver C.; Hungría A. B.; Conesa J. C.; Soria J. Structural Characteristics and Redox Behavior of CeO_2_-ZrO_2_/Al_2_O_3_ Supports. J. Catal. 2000, 194, 385 10.1006/jcat.2000.2931.

[ref64] Todorov N. D.; Abrashev M. V.; Marinova V.; Kadiyski M.; Dimowa L.; Faulques E. Raman spectroscopy and lattice dynamical calculations of Sc_2_O_3_ single crystals. Phys. Rev. B: Condens. Matter Mater. Phys. 2013, 87, 104301 10.1103/physrevb.87.104301.

[ref65] Zhang C.; Li S.; Wu G.; Gong J. Synthesis of stable Ni-CeO_2_ catalysts via ball-milling for ethanol steam reforming. Catal. Today 2014, 233, 53 10.1016/j.cattod.2013.08.013.

[ref66] James S. L.; Adams C. J.; Bolm C.; Braga D.; Collier P.; Friščić T.; Grepioni F.; Harris K. D. M.; Hyett G.; Jones W.; Krebs A.; Mack J.; Maini L.; Orpen A. G.; Parkin I. P.; Shearouse W. C.; Steed J. W.; Waddell D. C. Mechanochemistry: opportunities for new and cleaner synthesis. Chem. Soc. Rev. 2012, 41, 413 10.1039/c1cs15171a. 21892512

[ref67] Yang Y.; Zhang S.; Wang S.; Zhang K.; Wang H.; Huang J.; Deng S.; Wang B.; Wang Y.; Yu G. Ball milling synthesized MnO_x_ as highly active catalyst for gaseous POPs removal: significance of mechanochemically induced oxygen vacancies. Environ. Sci. Technol. 2015, 49, 4473 10.1021/es505232f. 25760959

[ref68] Alketbi M.; Polychronopoulou K.; Zedan A. F.; Sebastián V.; Baker M. A.; Alkhoori A.; Jaoude M. A.; Alnuaimi O.; Hinder S. S.; Tharalekshmy A.; Aljaber A. S. Tuning the activity of Cu-containing rare earth oxide catalysts for CO oxidation reaction: Cooling while heating paradigm in microwave-assisted synthesis. Mater. Res. Bull. 2018, 108, 142 10.1016/j.materresbull.2018.08.045.

[ref69] Ardelean H.; Frateur I.; Marcus P. Corrosion protection of magnesium alloys by cerium, zirconium and niobium-based conversion coatings. Corros. Sci. 2008, 50, 1907 10.1016/j.corsci.2008.03.015.

[ref70] Chen S.; Wang H.; Kang Z.; Jin S.; Zhang X.; Zheng X.; Qi Z.; Zhu J.; Pan B.; Xie Y. Oxygen vacancy associated single-electron transfer for photo- fixation of CO_2_ to long-chain chemicals. Nat. Commun. 2019, 10, 788 10.1038/s41467-019-08697-x. 30770824 PMC6377667

[ref71] Wang H.; Yong D.; Chen S.; Jiang S.; Zhang X.; Shao W.; Zhang Q.; Yan W.; Pan B.; Xie Y. Oxygen-vacancy-mediated exciton dissociation in BiOBr for boosting charge-carrier-involved molecular oxygen activation. J. Am. Chem. Soc. 2018, 140, 1760 10.1021/jacs.7b10997. 29319310

[ref72] Andriopoulou C.; Harris D.; Stephenson H.; Efstathiou A. M.; Boghosian S. In situ Raman Spectroscopy as a Tool for Discerning Subtle Structural Differences between Commercial (Ce,Zr)O2-Based OSC Materials of Identical Composition. Catalysts 2020, 10, 462 10.3390/catal10040462.

[ref73] Sing K. S. W.; Everett D. H.; Hall R. A. W.; Moscou L.; Pierotti R. A.; Rouquerol J. Reporting physisorption data for gas/solid systems with special reference to the determination of surface area and porosity. Pure Appl. Chem. 1985, 57, 603 10.1351/pac198557040603.

[ref74] Kumar P.; Srivastava V. C.; Mishra I. M. Synthesis and characterization of Ce-La oxides for the formation of dimethyl carbonate by transesterification of propylene carbonate. Catal. Commun. 2015, 60, 27 10.1016/j.catcom.2014.11.006.

[ref75] Ahfat N. M.; Buckton G.; Burrows R.; Ticehurst M. D. An exploration of inter-relationships between contact angle, inverse phase gas chromatography and triboelectric charging data. Eur. J. Pharm. Sci. 2000, 9, 271 10.1016/s0928-0987(99)00063-9. 10594384

[ref76] Feeley J. C.; York P.; Sumby B. S.; Dicks H. Determination of surface properties and flow characteristics of salbutamol sulphate, before and after micronisation. Int. J. Pharm. 1998, 172, 89 10.1016/s0378-5173(98)00179-3.

[ref77] aMukhopadhyay P.; Schreiber H. P. Aspects of acid-base interactions and use of inverse gas chromatography. Colloids Surf., A 1995, 100, 47 10.1016/0927-7757(95)03137-3.

[ref78] Binet C.; Daturi M.; Lavalley J.-C. IR study of polycrystalline ceria properties in oxidised and reduced states. Catal. Today 1999, 50, 207 10.1016/s0920-5861(98)00504-5.

[ref79] Vrijburg W. L.; Moioli E.; Chen W.; Zhang M.; Terlingen B. J. P.; Zijlstra B.; Filot I. A. W.; Züttel A.; Pidko E. A.; Hensen E. J. M. Efficient Base-Metal NiMn/TiO_2_ Catalyst for CO_2_ Methanation. ACS Catal. 2019, 9, 7823 10.1021/acscatal.9b01968.

[ref80] Köck E.-M.; Kogler M.; Bielz T.; Klötzer B.; Penner S. In Situ FT-IR Spectroscopic Study of CO_2_ and CO Adsorption on Y_2_O_3_, ZrO_2_, and Yttria-Stabilized ZrO_2_. J. Phys. Chem. C 2013, 117, 17666 10.1021/jp405625x. PMC3759166 24009780

[ref81] Ma D.; Lu Z.; Tang Y.; Li T.; Tang Z.; Yang Z. Effect of lattice strain on the oxygen vacancy formation and hydrogen adsorption at CeO2(111) surface. Phys. Lett. A 2014, 378, 2570 10.1016/j.physleta.2014.07.006.

[ref82] Yoshikawa K.; Sato H.; Kaneeda M.; Kondo J. N. Synthesis and analysis of CO_2_ adsorbents based on cerium oxide. J. CO_2_ Util. 2014, 8, 34 10.1016/j.jcou.2014.10.001.

[ref83] Reimers W. G.; Baltanás M. A.; Branda M. M. CO_2_ and H_2_ adsorption on ZnO, CeO_2_, and ZnO/CeO_2_ surfaces : DFT simulations. J. Mol. Model. 2014, 20, 2270 10.1007/s00894-014-2270-0. 24903980

[ref84] Huang J.; Li X.; Wang X.; Fang X.; Wang H.; Xu X. New insights into CO_2_ methanation mechanisms on Ni/MgO catalysts by DFT calculations: Elucidating Ni and MgO roles and support effects. J. CO_2_ Util. 2019, 33, 55 10.1016/j.jcou.2019.04.022.

[ref85] Ganduglia-Pirovano M. V.; Hofmann A.; Sauer J. Oxygen vacancies in transition metal and rare earth oxides: Current state of understanding and remaining challenges. Surf. Sci. Rep. 2007, 62, 219 10.1016/j.surfrep.2007.03.002.

[ref86] Zhu M.; Tian P.; Cao X.; Chen J.; Pu T.; Shi B.; Xu J.; Moon J.; Wu Z.; Han Y.-F. Vacancy engineering of the nickel-based catalysts for enhanced CO_2_ methanation. Appl. Catal., B 2021, 282, 119561 10.1016/j.apcatb.2020.119561.

[ref87] Roth J. P.; Pacchioni G. Influence of Strain on Acid-Basic Properties of Oxide Surfaces. J. Phys. Chem. C 2020, 124, 19126 10.1021/acs.jpcc.0c05913.

[ref88] Krcha M. D.; Janik M. J. Challenges in the Use of Density Functional Theory to Examine Catalysis by M-Doped Ceria Surfaces. Int. J. Quantum Chem. 2014, 114, 8 10.1002/qua.24548.

[ref89] Sun C.; Li H.; Chen L. Nanostructured ceria-based materials: synthesis, properties, and applications. Energy Environ. Sci. 2012, 5, 8475 10.1039/c2ee22310d.

[ref90] Wang B.; Xi X.; Cormack A. N. Chemical Strain and Point Defect Configurations in Reduced Ceria. Chem. Mater. 2014, 26, 3687 10.1021/cm500946s.

[ref91] Wu T. S.; Chen Y. W.; Weng S. C.; Lin C. N.; Lai C. H.; Huang Y. J.; Jeng H. T.; Chang S. L.; Soo Y. L. Dramatic band gap reduction incurred by dopant coordination rearrangement in Co-doped nanocrystals of CeO_2_. Sci. Rep. 2017, 7, 4715 10.1038/s41598-017-05046-0. 28680089 PMC5498595

[ref92] Heenemann M.; Millet M.-M.; Girgsdies F.; Eichelbaum M.; Risse T.; Schlögl R.; Jones T.; Frei E. The Mechanism of Interfacial CO2 Activation on Al Doped Cu/ZnO. ACS Catal. 2020, 10, 5672 10.1021/acscatal.0c00574.

[ref93] Cheng Z.; Sherman B. J.; Lo C. S. Carbon dioxide activation and dissociation on ceria (110): A density functional theory study. J. Chem. Phys. 2013, 138, 014702 10.1063/1.4773248. 23298052

[ref94] Yu Y.; Mao B.; Geller A.; Chang R.; Gaskell K.; Liu Z.; Eichhorn B. W. CO2activation and carbonate intermediates: an operando AP-XPS study of CO2electrolysis reactions on solid oxide electrochemical cells. Phys. Chem. Chem. Phys. 2014, 16, 11633 10.1039/c4cp01054j. 24806971

[ref95] Alayoglu S.; Krier J. M.; Michalak W. D.; Zhu Z.; Gross E.; Somorjai G. A. In Situ Surface and Reaction Probe Studies with Model Nanoparticle Catalysts. ACS Catal. 2012, 2, 2250 10.1021/cs3004903.

[ref96] Coenen K.; Gallucci F.; Mezari B.; Hensen E.; van Sint Annaland M. An in-situ IR study on the adsorption of CO_2_ and H_2_O on hydrotalcites. J. CO_2_ Util. 2018, 24, 228 10.1016/j.jcou.2018.01.008.

[ref97] Dou B.; Zhao L.; Zhang H.; Wu K.; Zhang H. Renewable hydrogen production from chemical looping steam reforming of biodiesel byproduct glycerol by mesoporous oxygen carriers. Chem. Eng. J. 2021, 416, 127612 10.1016/j.cej.2020.127612.

[ref98] Wu K.; Dou B.; Zhang H.; Liu D.; Chen H.; Xu Y. Aqueous phase reforming of biodiesel byproduct glycerol over mesoporous Ni-Cu/CeO2 for renewable hydrogen production. Fuel 2022, 308, 122014 10.1016/j.fuel.2021.122014.

[ref99] Luo C.; Dou B.; Zhang H.; Liu D.; Zhao L.; Chen H.; Xu Y. Co-production of hydrogen and syngas from chemical looping water splitting coupled with decomposition of glycerol using Fe-Ce-Ni based oxygen carriers. Energy Convers. Manage. 2021, 238, 114166 10.1016/j.enconman.2021.114166.

