# Supplementary material for: Decoupling the Chemical and Mechanical Strain Effect on Steering the CO2 Activation over CeO2-Based Oxides: An Experimental and DFT Approach
Source: ACS Appl Mater Interfaces. 2022 Jul 12;14(29):33094–119. doi: 10.1021/acsami.2c05714 (PMC9335529; doi:10.1021/acsami.2c05714)
Supplement: Supplementary file 1 — am2c05714_si_001.pdf [file am2c05714_si_001.pdf]

Supporting Information

Decoupling the chemical and mechanical strain effect on steering the CO_2 activation over CeO_2-based oxides: An experimental and DFT approach

Kyriaki Polychronopoulou[1,2,*], Sara AlKhoori[1,2,$], Shaima AlBedwawi[1,2,$], Seba Alareeqi[2,3], Aseel G.S. Hussien[1,2], Michalis A. Vasiliades[4], Angelos M. Efstathiou[4], Klito C. Petallidou[4], Nirpendra Singh[2,5], Dalaver H. Anjum[2,5], Lourdes F. Vega[2,3], Mark A. Baker[6]

[1]Department of Mechanical Engineering, Khalifa University of Science and Technology, Abu Dhabi, P.O. Box 127788, United Arab Emirates

[2]Center for Catalysis and Separations (CeCaS Center), Khalifa University of Science and Technology, Abu Dhabi, P.O. Box 127788, United Arab Emirates

[3]Department of Chemical Engineering and Research and Innovation Center on CO2 and Hydrogen (RICH Center), Khalifa University of Science and Technology, Abu Dhabi, P.O. Box 127788, United Arab Emirates

[4]Department of Chemistry, Heterogeneous Catalysis Laboratory, University of Cyprus, 1 University Ave., University Campus, 2109 Nicosia, Cyprus

[5]Department of Physics, Khalifa University of Science and Technology, Abu Dhabi, P.O. Box 127788, United Arab Emirates

[6]The Surface Analysis Laboratory, Faculty of Engineering and Physical Sciences, University of Surrey, Guildford, GU2 4DL, UK

$: equal contribution

***corresponding author:** Kyriaki Polychronopoulou (kyriaki.polychrono@ku.ac.ae)

1. Materials Characterization

Porosimetry measurements were conducted using high-resolution 3Flex Micromeritics (Atlanta, USA) adsorption instrument that has high-vacuum system and three 0.1 Torr pressure transducers. Prior to the analysis, the samples were degassed at 150°C overnight to remove any residual moisture. The Brunauer-Emmett-Teller (BET) specific surface area was calculated using the adsorption data obtained within the relative pressure range of P/P_o= 0.04 - 0.25, where the Barrett-Joyner-Halenda (BJH) method was implemented to evaluate the pore size distribution and pore volume.

The crystal structure of the calcined catalysts was studied by D2-Phaser® XRD instrument (Bruker, MA, USA) using Cu Kα source with excitation wavelength of 1.5418 Å. The X-ray generator was working at 30 kV and 20 mA. A 2θ scan range was set to be between 10-100° with a 0.05° step size. The average crystallite size was calculated based on the predominant (111) peak using Scherrer formula (see Eq. 15)[2]. The lattice parameter was evaluated using standard cubic indexation method based on the (111) peak. The Williamson-Hall equation (see Eq. 16) was implemented to calculate the lattice strain, using the intense peaks (111), (200), (220) and (311)[2].

$$D = \frac{0.94\lambda}{\beta\cos\theta} \qquad \text{Scherrer Formula} \qquad (1)$$

$$\beta\cos\theta = \frac{0.94\lambda}{D} + 4\epsilon\sin\theta \qquad \text{Williamson} - \text{Hall Equation} \qquad (2)$$

Complementary structural information such as oxygen sublattice were received using Witec Alpha 300 Raman spectroscopy (Germany) that is equipped with 532 nm laser and research grade optical microscope with various lenses. The instrument features a manual sample positioning with both planar (x,y-direction) and depth scans (z-direction). All the catalysts spectra were acquired using single-point Raman spectrum acquisition.

Transmission electron microscopy (TEM) analysis was carried out for determining the size, crystal structure, and elemental distribution in samples. It has been accomplished by employing a TEM of model Titan G^2 80-300 ST from Thermo-Fisher Scientific (Waltham, MA) that was also equipped with a spherical aberration corrector for the image, scanning TEM (STEM) mode, an

energy-filter of model GIF-Quantum 963. The microscope was operated the accelerating voltage of 300 kV during the analysis. First, several low-to-medium range magnifications bright field TEM (BF-TEM) images were acquired to investigate the size and morphology of $CeLaCuO_x$ crystallites present in the samples. Selected area electron diffraction (SAED) was performed next to determine the phase and nano structure of samples. These both quantities have also been investigated by using aberration corrected high-resolution TEM (HR-TEM). This analysis was further utilized to reveal the faceting of crystallites in samples. The composition of elements in samples was determined by using the electron energy loss spectroscopy (EELS) in TEM mode. However, the spatial distribution analysis of elements was carried out by setting the microscope in STEM mode along with simultaneous EELS spectroscopy. It is to be noted that O-K, La-M45, Ce-M45, Cu-L23 edges at the energy loss values of 532 eV, 832 eV, 883 eV, and 931 eV were selected for oxygen, lanthanum, cerium, and copper elements, respectively. During the data acquisition the scanning of beam was carried out with spatial pixel size of 0.55 nm for STEM and energy dispersion of 1 eV for corresponding EELS spectra. The STEM-EELS datasets were acquired and post processed in Gatan Microscopy Suite of version GMS 3.2. Multiple linear least-square (MLLS) routines incorporated in GMS 3.2 package were applied to acquired datasets in order to generate high quality and fidelity elemental maps for samples.

The EPR spectra were recorded under the following conditions: Frequency = 9.65 GHz, Power = 1.0 mW, Modulation Frequency = 100 kHz, Modulation Amplitude = 1 G, Time Constant = 20.48 ms, Conversion Time = 20.00 ms and ambient temperature.

The catalysts morphology was studied using JSM 7610F-Field Emission Scanning Electron Microscope (JEOL Ltd., Tokyo, JPN), where secondary electron imaging was employed. The instrument is equipped with energy dispersive X-ray spectroscopy (EDX) along with Oxford $XMax^N$ 50 mm^2 silicon drift detector, which was considered to perform qualitative analysis using AZtecEnergy analysis software (Oxford Instruments, United Kingdom).

The catalysts reducibility was evaluated through H_2-TPR experiments in which 10 vol%H_2/Ar gas mixture (30 NmL/min) is passed over 60 mg sample at a temperature rate of 30°C/min after the pre-treatment with 20 vol%O_2/He (500°C, 1h) while the Thermal Conductivity Detector (TCD) signal was recorded simultaneously. The sample was mounted on a quartz wool bedding in a U-shaped tube and placed in a flow through the reactor (Autochem 2920, Micromeritics, Atlanta,

USA). The H_2 (m/z=2) and H_2O (m/z=18) signals were tracked using an online Cirrus mass spectrometer.

The catalysts surface basicity was probed using Autochem 2920, (Micromeritics, Atlanta, USA). Similar to TPR, the sample was mounted on a quartz wool bedding in a U-shaped tube and placed in a flow through the reactor. The gas mixture 5 vol.% CO_2/Ar was allowed to pass over 60 mg sample for 30 min (30 NmL/min) after the pre-treatment with 20 vol%O_2/He (500°C, 1h) using a temperature ramp of 30°C/min. The CO (m/z=28) and CO_2 (m/z=44) signals were tracked using an online Cirrus mass spectrometer.

The catalysts surface acidity was probed using Autochem 2920, (Micromeritics, Atlanta, USA). Similar to TPR, the sample was mounted on a quartz wool bedding in a U-shaped tube and placed in a flow through the reactor. The gas mixture 2 vol.% NH_3/He was allowed to pass over 60 mg sample for 1 h (30 NmL/min) after the pre-treatment with 20 vol%O_2/He (500°C, 1h) using a temperature ramp of 30°C/min. The NH_3 (m/z=17) signal was tracked using an online Cirrus mass spectrometer.

The surface elemental concentrations and chemical states were analyzed by XPS that was performed on a ThermoFisher Scientific (East Grinstead, UK) K-Alpha$^+$ spectrometer. All the XPS spectra were obtained using a monochromated Al Kα X-ray source (hν=1486.6 eV), with an X-ray spot of 400 μm radius. The survey spectra were acquired using a pass energy of 300 eV, while the high-resolution core-level spectra had a pass energy of 50 eV with energy step size of 0.1 eV. The C 1s peak at 285.0 eV was considered for charge reference to correct any charging effect during the run. The quantitative surface chemical analysis was calculated based on the high-resolution core-level spectra peak fitting, using the manufacturer's Avantage software.

The dispersion of the Ni supported catalysts was assessed using H_2-TPD (temperature programmed desorption). The H_2-TPD technique was used to investigate the hydrogen adsorption states over the supported Ni catalysts. The catalyst sample was initially calcined at 750 ◦C/4 h. Then, 0.1 g of sample was loaded in the reactor, followed by temperature increase under He gas flow to 750 ◦C. The next step was reduction of the sample under hydrogen gas flow (1 bar) at 750 ◦C/2 h, followed by purging under He at 750 ◦C until the H_2-mass spectrometer signal get its background value. Cooling of the catalyst followed till 30 ◦C under He flow and a 30-min exposure to a 0.5 vol% H_2/He adsorption gas. H-spillover was avoided under these chemisorption conditions; use of lower

or higher adsorption times in H_2/He resulted in very similar chemisorption amounts. After H2 chemisorption step, purge of the sample followed for 10 min and its temperature was then increased to 700 ◦C (β = 30 ◦C min− 1, H_2-TPD). The H_2 signal (*m/z* = 2) was monitored in continuous basis with online mass spectrometer (MS, Balzers, Omnistar 1-200 amu) and converted into concentration (mol%) using a certified gas mixture (0.95 vol% H_2/He). The rate of H2 desorption was estimated based on the material balance (open-flow reactor). Based on the nickel dispersion, D_{Ni} (%), the mean particle size, (dNi, nm) was estimated.

2. Results and Discussion

Structural Properties-Doping Effect: **Figure S1(A)** compares the **XRD patterns** of the mixed metal oxide catalysts, which are acquired following calcination of 500˚C for 6 h. By comparing the XRD pattern of the pure CeO_2 (JCPDS- 34-0394) with the mixed oxide catalysts, it can be stated that enhanced microwave synthesis resulted in the formation of solid solutions with cubic structure. The main ceria fluorite cubic lattice has characteristic reflections of (111), (200), (220), (311), (222) and (400) at diffraction angles of 28.5˚, 33˚, 47.5˚, 56.3˚, 59.1˚ and 69.3˚, respectively. However, in the case of mixed oxides, they have been shifted to lower diffraction angles due to the insertion of larger sized La^{3+} (1.1 Å) ions into CeO_2 cubic structure (Ce^{4+}: 0.97 Å)[3], which caused increase of the cell lattice parameter upon doping (Table 1). For the **Ce-La-10Cu-O catalyst**, additional minor peaks at 35.1˚ and 38.2˚ are observed, which indicates the formation of separate crystalline CuO phase due to uneven dispersion of Cu species[4]. It is suggested that in the case of this oxide a further lattice distortion and crystallite size contraction to 11 nm is also caused by the simultaneous incorporation of the La^{3+} (bigger than Ce^{4+}) and smaller Cu^{2+} dopant (Cu^{2+}: 0.73 Å) into ceria lattice [54]. This in fact determines the significance of Cu to suppress Ce-La-O/Cu-doped CeO_2 crystal growth and enhance its thermal stability[4]. **SEM studies** (**Figure S1B&C)** showed that doping ceria with La leads to the formation of flake-like morphology. It should be emphasized that doping with 10% Cu yields to formation of rather spherical CuO nanoparticles which were confirmed through EDS analysis[4]. These results are in agreement with the presented XRD results which supported the existence of CuO phase impurity which inhibits the growth of ceria-based phase, giving rise to particles with crystal size of only 11.3 nm.

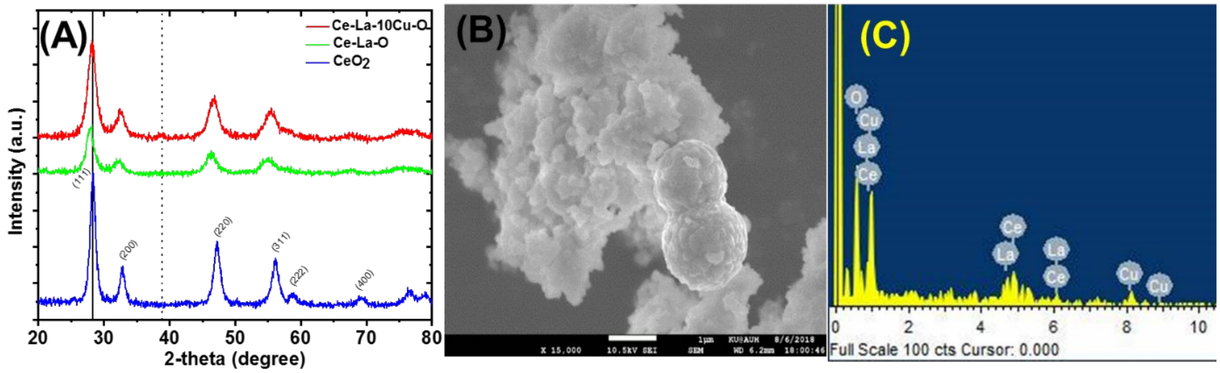

Figure S1: (A) XRD profiles of the Ce-La10Cu-O and the reference oxides Ce-La-O and CeO2; **(B-C)** SEM microphotograph along with EDX analysis of the Ce-La10Cu-O.

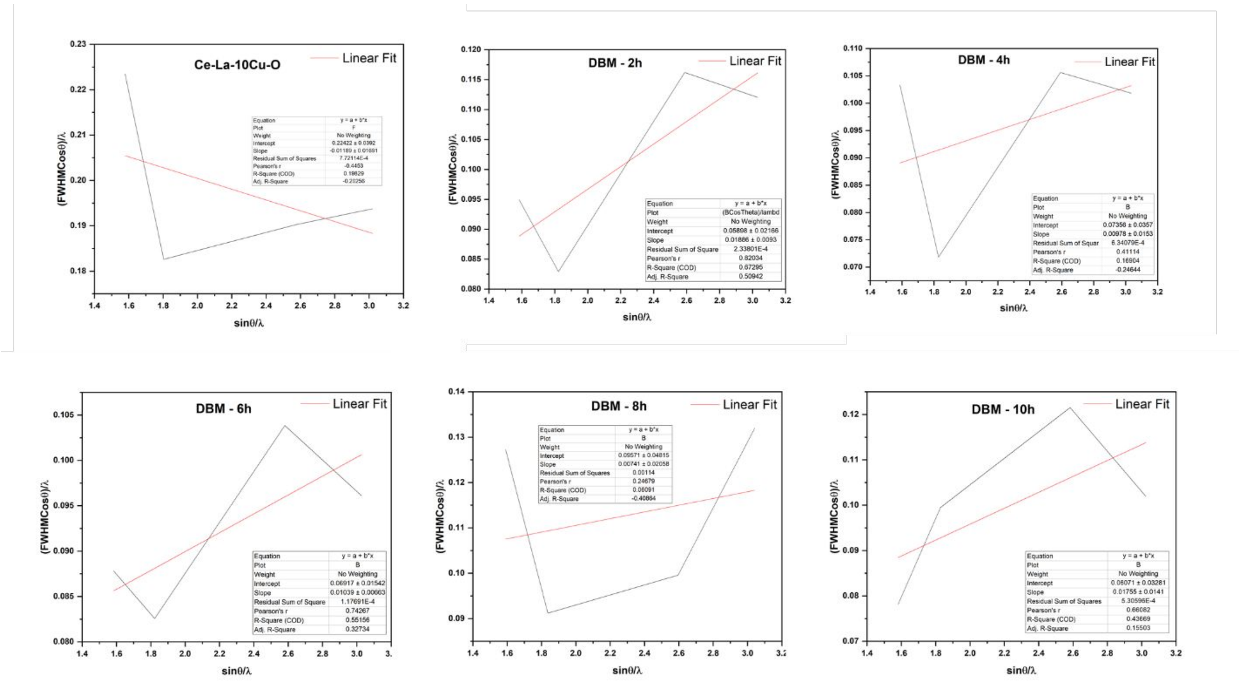

Figure S2: Williamson-Hall plots

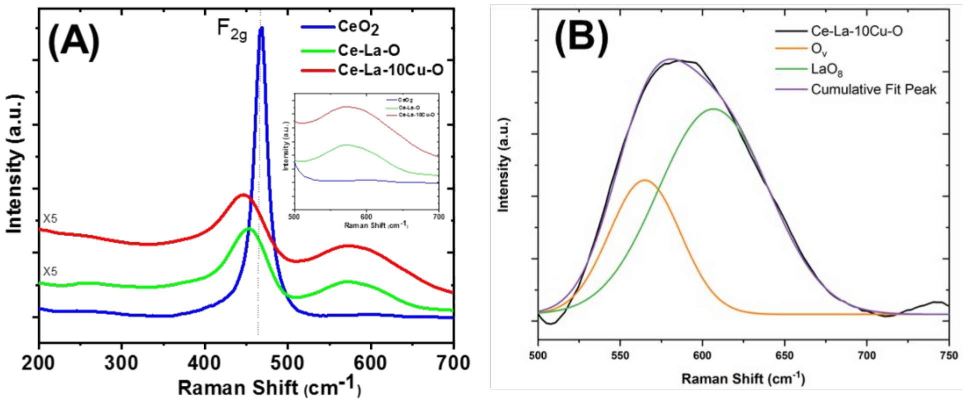

Figure S3: Raman spectra of the reference oxides CeO_2, Ce-La and Ce-La-Cu-O

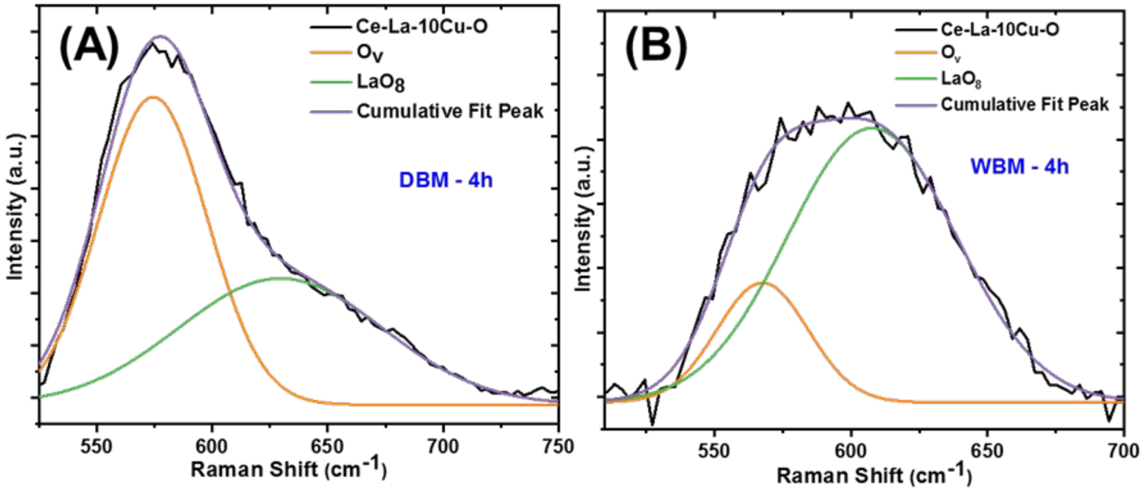

Figure S4: Deconvoluted Raman spectra in the defect region of 550-650 cm^{-1} for the DBM **(A)** and WBM **(B)**, respectively.

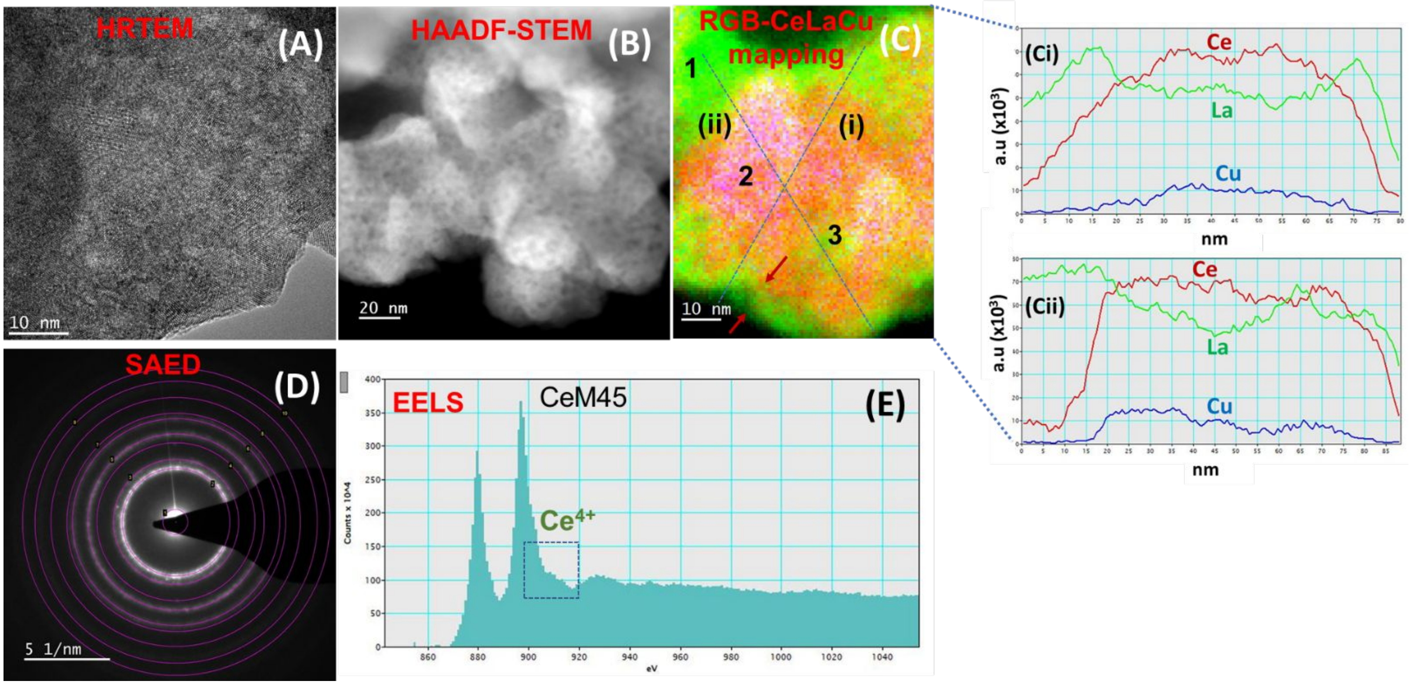

Figure S5: (A) HRTEM, **(B)** STEM-HAADF, **(C)** Red Green Blue (RGB) mapping, **(D)** SAED, **(E)** EELS obtained over the fresh Ce-La-10Cu-O oxide.

Table S1: Results are collected from a Ce-O near the surface Ov at site 2.

Strain	Ce-O Length (Angstroms)	O-Ce-O Angle (degrees)
System: CeO_2		
+5%	2.34	165.49
0%	2.26	166.05
-5%	2.19	164.29
System: Ce-La-O		
+5%	2.54	164.04
0%	2.44	169.66
-5%	2.29	169.39
System: Ce-La-Cu-O		
+5%	2.51	157.73
0%	2.63	160.47
-5%	2.24	167.94

Table S2: SAED data for Ce-La-10Cu-O

Ring	Radius (1/nm)	1/Radius (nm)
1	3.140	0.318
2	3.647	0.274
3	4.603	0.217

4	5.140	0.195
5	6.028	0.166
6	6.365	0.157
7	7.258	0.138
8	8.215	0.122
9	8.939	0.112

Table S3: SAED data for DBM/4 h Ce-La-10Cu-O

Ring	Radius (1/nm)	1/Radius (nm)
1	3.190	0.314
2	3.677	0.272
3	5.204	0.192
4	6.072	0.165
5	7.309	0.137
6	7.996	0.125
7	9.041	0.111
8	10.827	0.092

Textural studies

The textural properties including particle size and specific surface area (m^2/g) are presented in **Table 1 and Fig. S6**. Generally, doping ceria with La and Cu results in a significant decrease in BET surface area from 36.6 m^2/g to 5.8 and 4.9 m^2/g, respectively. This can be justified based on the high dopant content, where both La and Cu has a nominal composition ratio of 1.2:1 compared to the host material (Ce). Thus, it is anticipated that some agglomerates or phases undetectable by the XRD form and block the active surface area[4]. Researchers have investigated the effect of surface area and interestingly they found that it is of a significance in the total OSC of ceria-based catalysts[5]. It has been reported that the addition of La and Cu generally improves the oxygen storage capacity of ceria which is attributed to the increase of relative surface concentration of Ce^{3+} species[6,7]. Hence, more oxygen vacant sites are expected to form, which will eventually facilitate oxygen mobility from the bulk towards the surface.

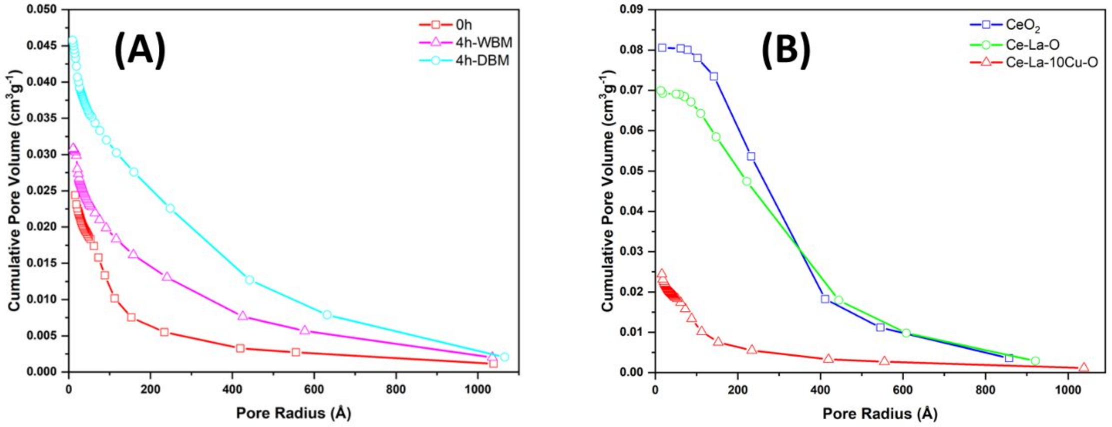

Figure S6: Pore size distribution of **(A)** pristine and ball milled samples; **(B)** reference oxides

Figure 5A presents the H_2-TPR profiles of all the catalytic oxides of this study. Based on the literature, pure ceria shows a reduction peak around 247°C which is correlated to sub surface reduction[8], and other two peaks at higher temperatures (III) around 467 and 1000°C correspond to the formation of non-stoichiometric oxides of ceria[9]. Zhu et al.[10] and Fu et al.[11] found that cerium oxide can be reduced between 280 and 300°C, so the low reduction temperature profiles starting at 100°C are associated to the activation of oxygen species of the cubic fluorite structure with the incorporation of La^{3+}/Cu^{2+} species and the $Cu^{2+} \rightarrow Cu^{+} \rightarrow Cu^{0}$ successive reduction processes involved[1]. A shoulder peak around 210°C emerges upon doping with Cu, which is linked to either the reduction of surface ceria species where Cu oxides are dispersed on it or to the formation of separate CuO crystalline phase on ceria, according to Li et al.[12]. It is noted that doping Ce-La-O with 10% Cu enhances the catalyst reducibility, particularly in the low (<250°C) and medium (<500°C) temperature regimes, while no significant changes observed at higher (>550°C) regimes compared to ceria. The results obtained here are consistent with the literature where it is reported that integrating dopants into ceria lattice basically facilitates the reducibility of the lattice[13].

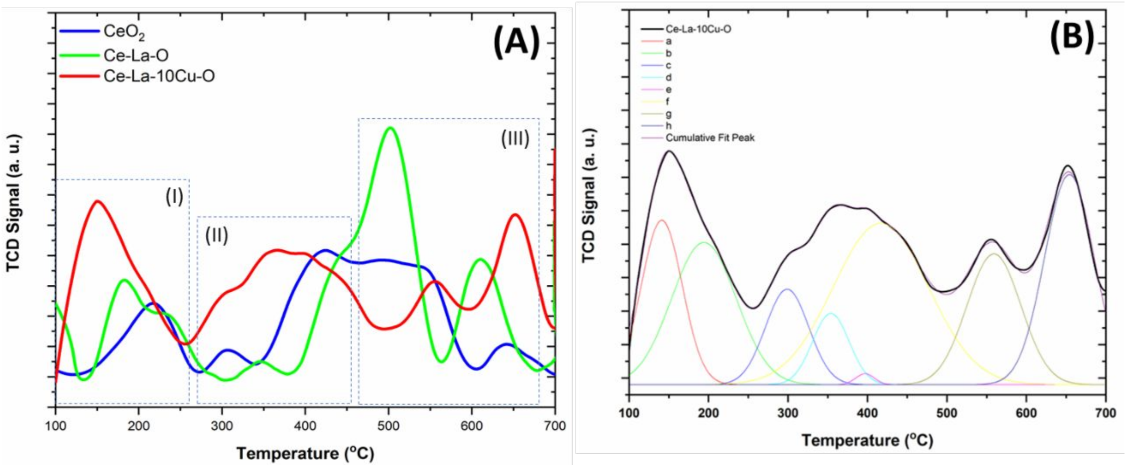

Figure S7: (A) H_2-TPR profiles of the refence oxides; **(B)** Deconvoluted H_2-TPR for the pristine Ce-La-10Cu-O oxide.

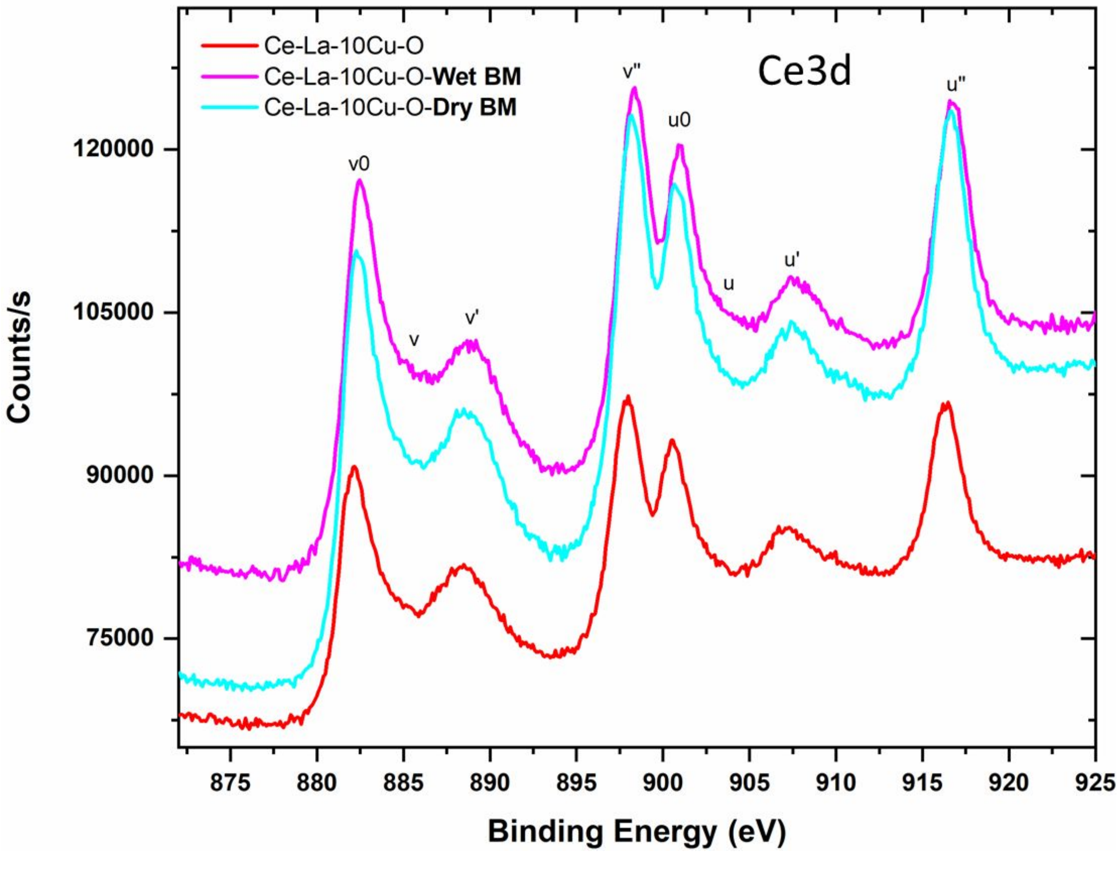

Figure S8: XPS Ce3d core level spectrum obtained over the Ce-La-10Cu-O oxide before and after ball milling (WBM, DBM).

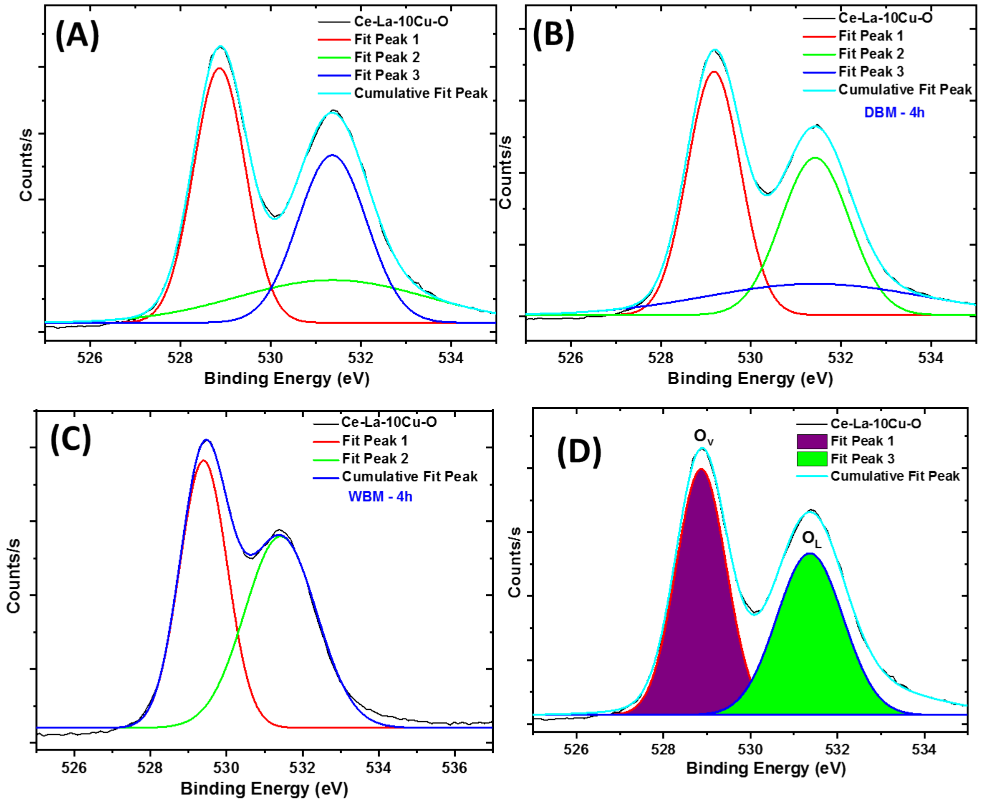

Figure S9: XPS O1s core level spectrum of the **(A)** pristine Ce-La-Cu-O; **(B)** Ce-La-Cu-O DBM; **(C)** Deconvoluted Ce-La-Cu-O WBM; **(D)** areas calculation of the deconvoluted O1s spectrum.

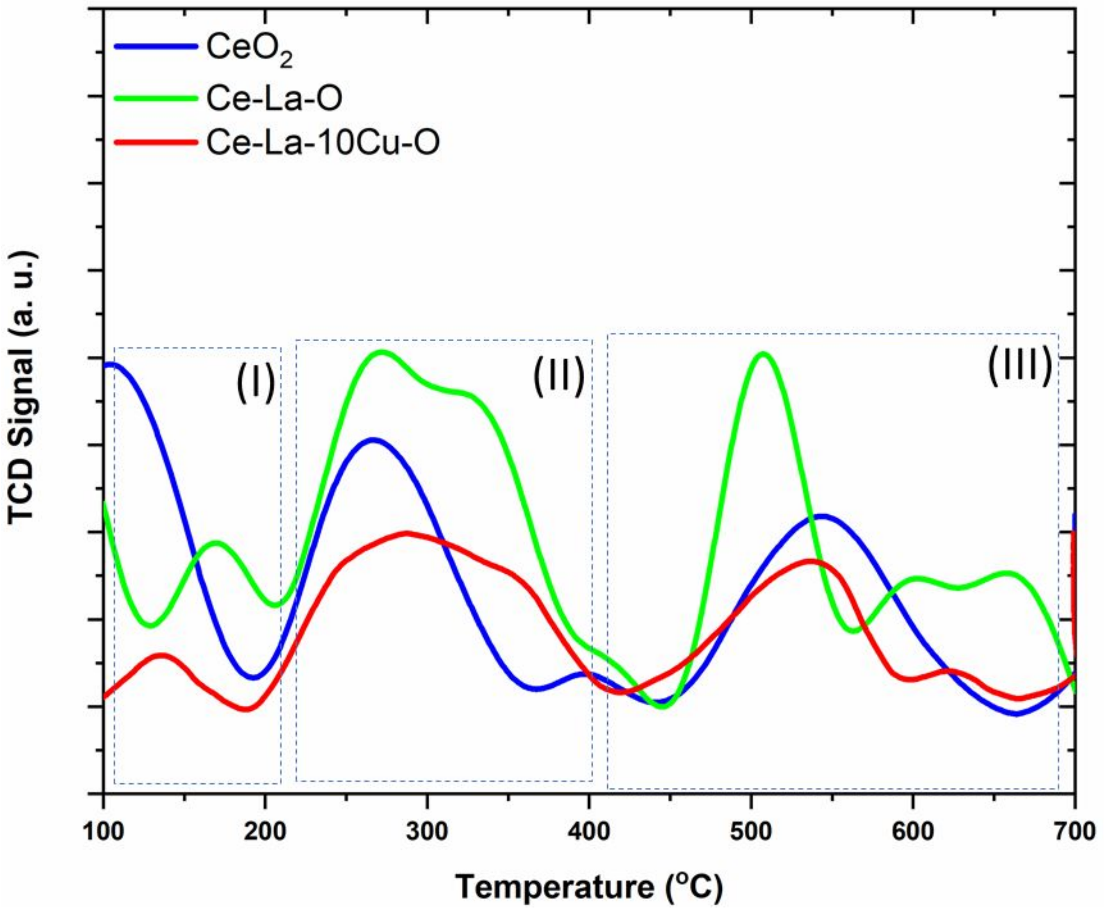

Figure S10: CO2-TPD recorded over the reference oxides of this study.

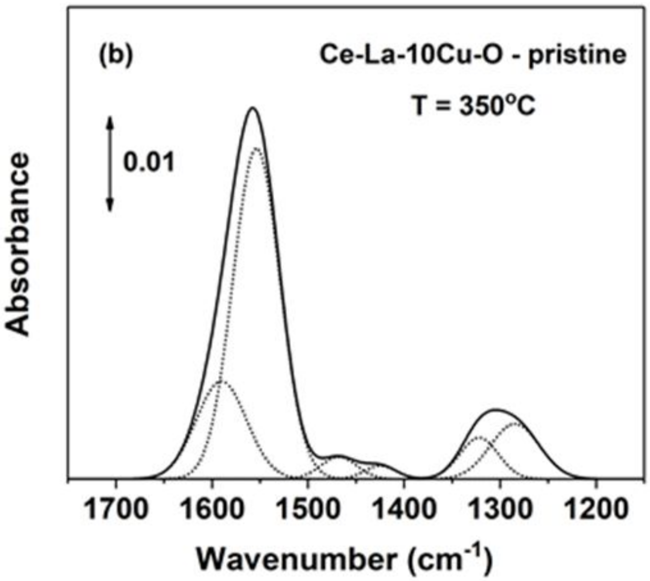

Figure S11: Deconvoluted IR spectra of Ce-La-10Cu-O – pristine recorded under 5%CO_2/He (30 min) gas mixture.

Table S3: Peak maximum (cm^{-1}) and area of IR bands in the 1750-1150 cm^{-1} range.

**Pristine**		**Dry BM**	
**Peak maximum (cm^{-1})**	**Area**	**Peak maximum (cm^{-1})**	**Area**
1590	0.68	1590	1.10
1554	2.14	1554	0.56
1468	0.11	1468	0.06
1425	0.06	-	-
1322	0.22	1322	0.08
1285	0.37	1285	0.23

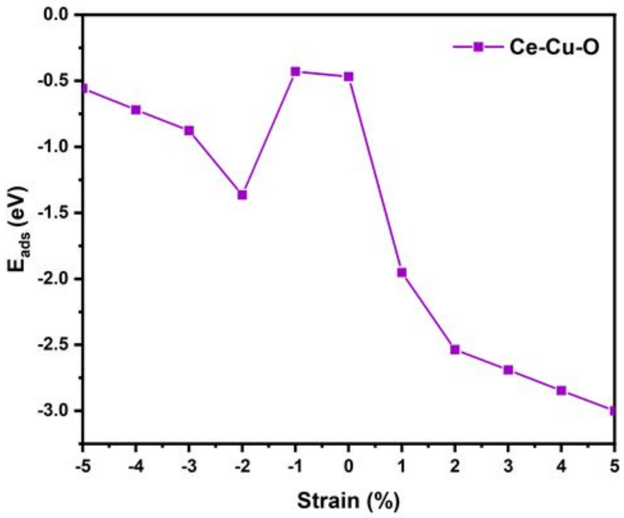

Figure S12: (2×2) Ce-Cu-O oxygen vacancy energy of formation (E_{Ov}) under the biaxial strain for single surface (SSV).

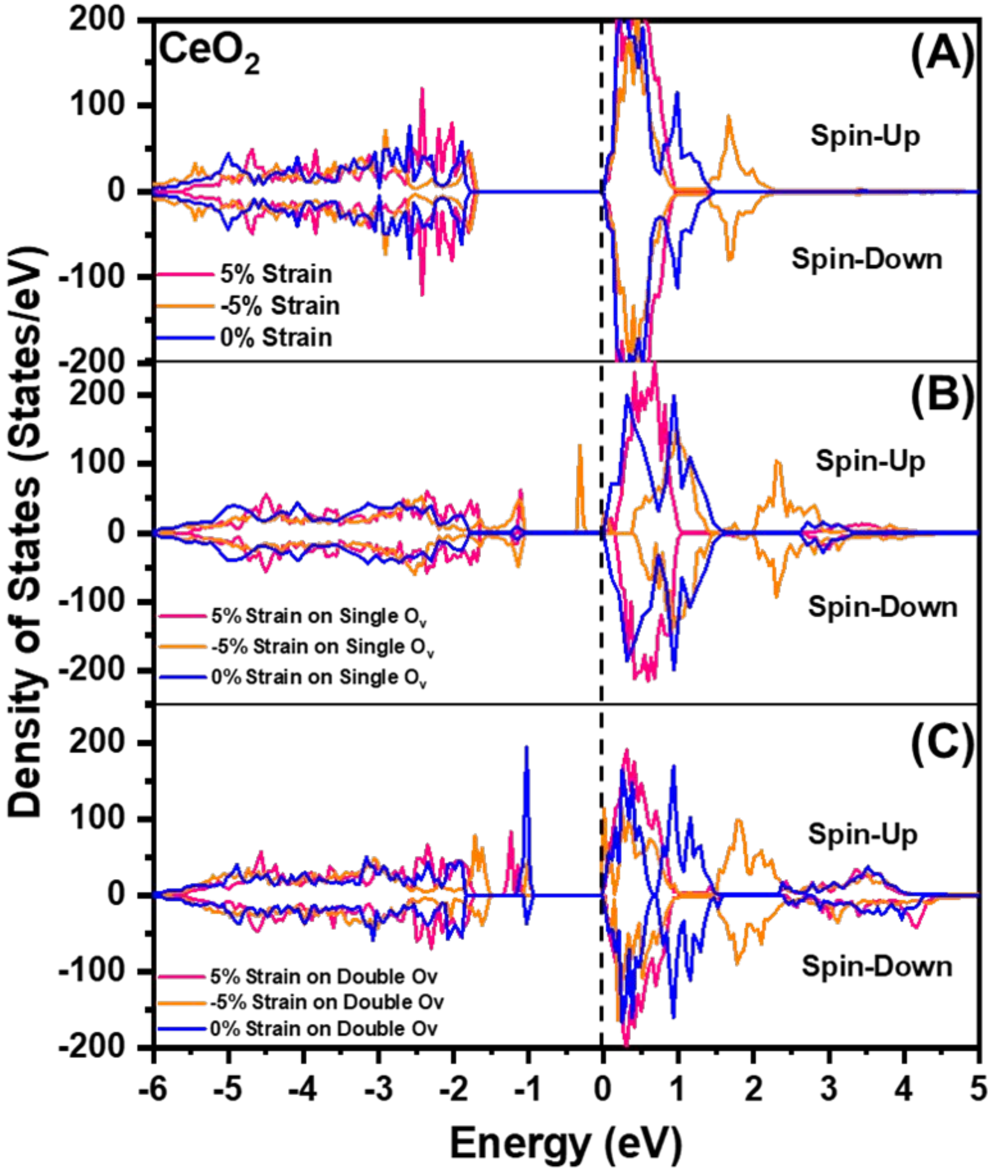

Figure S13. Density of states (DOS) of CeO_2(111) under −5, 0, and +5% strain with different configurations of **(A)** perfect surface with no defects, **(B)** single oxygen vacancies, and **(C)** double oxygen vacancies. The dashed vertical line represents the Fermi level.

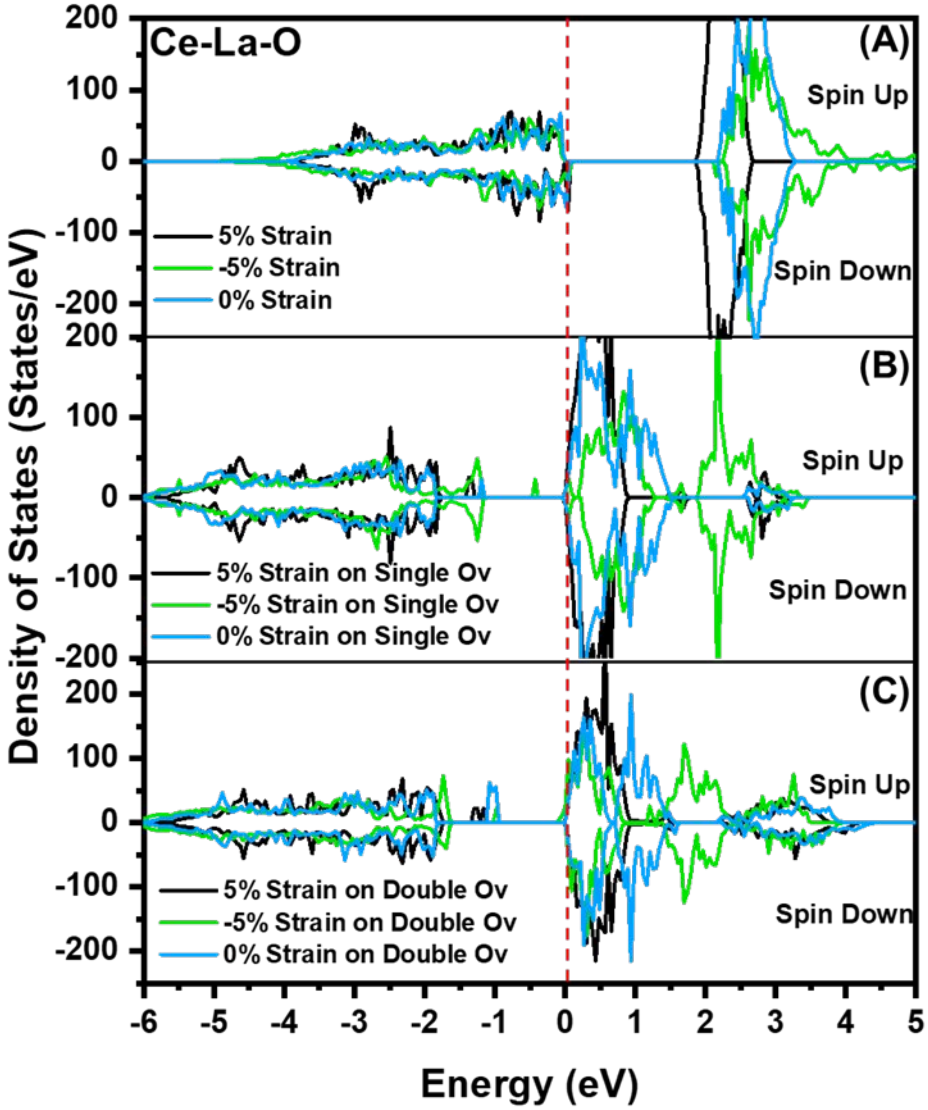

Figure S14. Density of states (DOS) of Ce-La-O dopped CeO_2(1 1 1) under −5, 0, and +5% strain with different configurations of **(A)** perfect surface with no defects, **(B)** single oxygen vacancies, and **(C)** double oxygen vacancies. The Dashed vertical line represents the Fermi level.

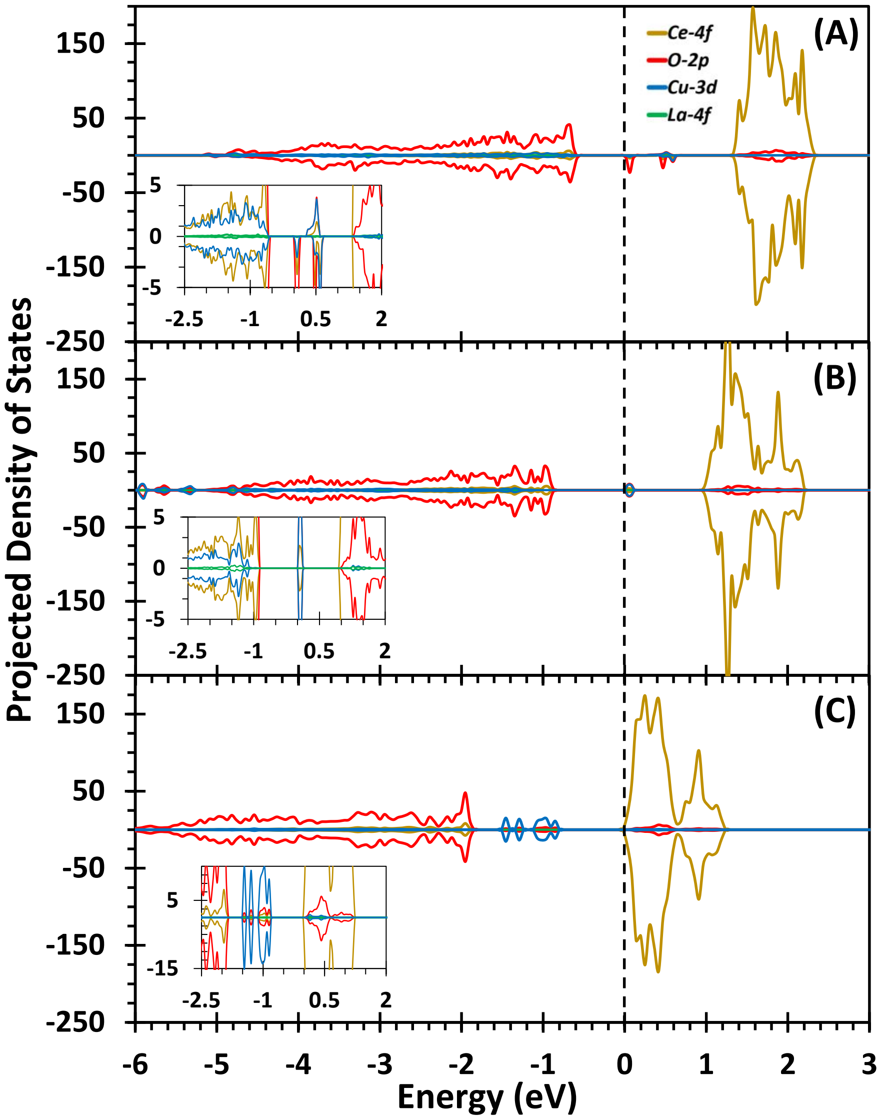

Figure S15: PDOS of Ce-La-Cu-O (1 1 1) under 0% strain level with different configurations of **(A)** clean surface, **(B)** reduced surface, single oxygen vacancy, and **(C)** reduced surface, double oxygen vacancies. The dashed vertical line represents the Fermi level (Inserted figure is a partial enlarged view).

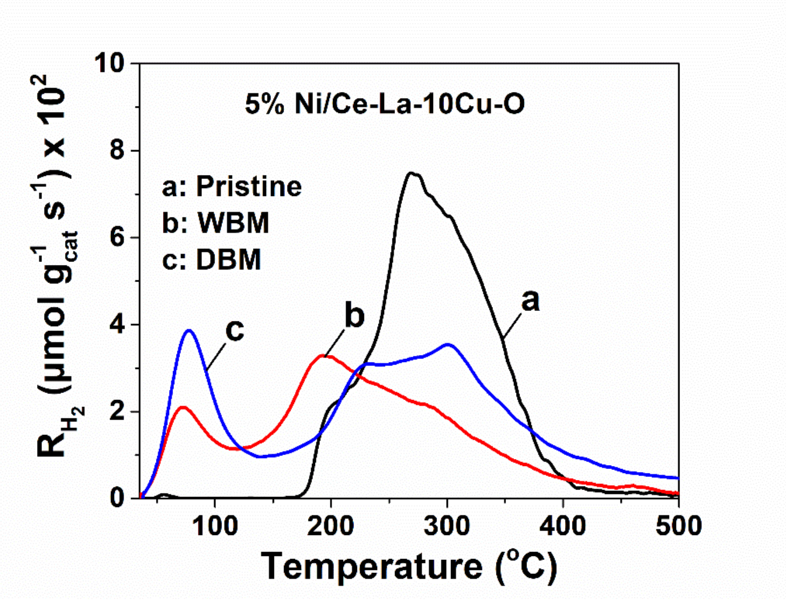

Fig. S16. H_2-TPD traces in terms of hydrogen desorption rate (μmol g^{-1}_{cat} s^{-1}) obtained on 5 wt% Ni/Ce-La-10Cu-O catalysts: (a) Pristine, (b) WBM and (c) DBM. F_{He} = 50 NmL min^{-1} ; β = 30 ◦C min^{-1}; W_{cat} = 0.1 g.

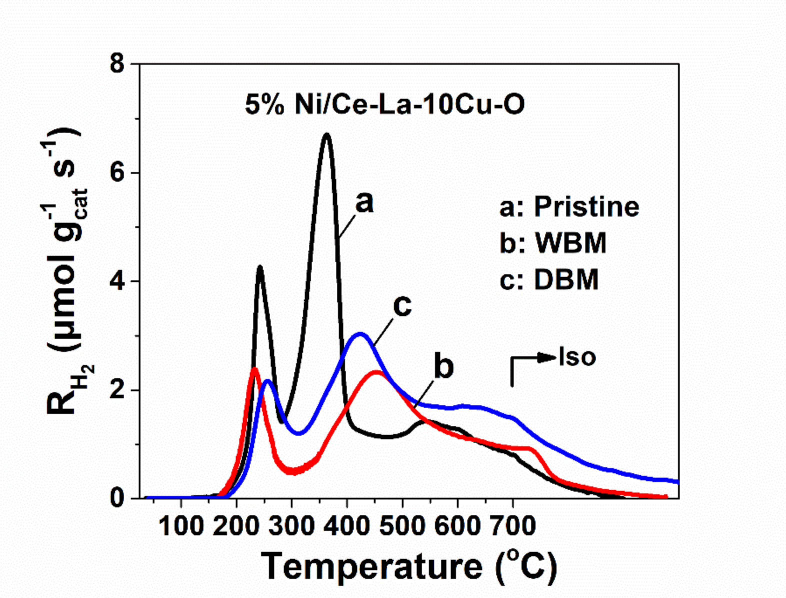

Fig. S17. H_2-TPR traces in terms of hydrogen reduction rate (μmol g^{-1}_{cat} s^{-1}) obtained over the 5 wt% Ni/Ce-La-10Cu-O catalysts: (a) Pristine, (b) WBM and (c) DBM. F_T = 50 NmL min^{-1} of 5.18 vol% H_2/He; β = 30 °C min^{-1}; W_{cat} = 0.1 g.

Calculated Total Energies of CeO_2, Ce-La-O and Ce-La-Cu-O (111) Surfaces by DFT

Table S4: Total energies in eV of CeO_2 (111) with single surface oxygen vacancies at locations 1,2,3 and 4 under tensile and compressive strain.

	Single Surface Oxygen Vacancies			
**Strain (%)**	**1**	**2**	**3**	**4**
**+5**	1.37	1.16	1.37	1.37
**+4**	1.64	1.64	1.63	1.63
**+3**	1.90	1.88	1.89	1.88
**+2**	2.11	2.12	2.13	2.11
**+1**	2.36	2.36	2.36	2.36
**0**	2.64	2.66	2.65	2.65
**-1**	2.82	2.96	2.82	2.69
**-2**	3.19	3.21	3.02	3.02
**-3**	3.41	3.41	3.41	3.22
**-4**	3.40	3.41	3.65	3.50
**-5**	3.90	3.89	3.87	3.75

Table S5: Total energies in eV of CeO_2 (111) with single sub-surface oxygen vacancies at locations 5, 6, 7 and 8 under tensile and compressive strain.

	Single Subsurface Oxygen Vacancies			
**Strain (%)**	**5**	**6**	**7**	**8**
**+5**	1.53	1.11	1.53	1.53
**+4**	1.37	1.80	1.74	1.44
**+3**	1.75	1.47	1.47	1.75
**+2**	1.70	2.10	1.70	1.69
**+1**	1.92	2.09	1.92	2.11
**0**	2.20	1.96	2.20	1.95
**-1**	2.08	2.23	2.38	2.36
**-2**	2.28	2.60	2.57	2.27
**-3**	2.77	2.55	2.77	2.55
**-4**	2.80	2.80	2.61	2.70
**-5**	2.95	3.32	2.87	2.76

Table S6: Total energies in eV of CeO_2 (111) with double surface oxygen vacancies at locations 1-2 and 3-4 under tensile and compressive strain.

Double Surface Oxygen Vacancies		
**Strain (%)**	**1-2**	**3-4**
**+5**	1.76	1.76
**+4**	2.02	1.96
**+3**	2.15	2.15
**+2**	2.34	2.41
**+1**	2.53	2.53
**0**	2.76	2.76
**-1**	2.89	2.89
**-2**	3.06	3.06
**-3**	2.97	2.97
**-4**	2.98	3.13
**-5**	2.91	2.95

Table S7: Total energies in eV of CeO_2 (111) with double sub-surface oxygen vacancies at locations 6-8, 5-6, 7-8 and 5-8 under tensile and compressive strain.

Double Subsurface Oxygen Vacancies				
**Strain (%)**	**6-8**	**5-6**	**7-8**	**5-8**
**+5**	1.54	1.54	1.54	1.54
**+4**	1.70	1.70	1.79	1.79
**+3**	1.85	1.85	1.85	1.85
**+2**	2.19	2.10	2.10	2.10
**+1**	2.36	2.36	2.18	2.18
**0**	2.32	2.17	2.34	2.37
**-1**	2.42	2.27	2.27	2.27
**-2**	2.40	2.40	2.40	2.40
**-3**	2.55	2.53	2.53	2.53
**-4**	2.65	2.65	2.65	2.65
**-5**	2.75	2.75	2.75	2.75

Table S8: Total energies in eV of CeO_2 (111) with double sub-surface and surface oxygen vacancies at locations 1-5, 1-7, 3-8, 7-4, 2-6 and 2-5 under tensile and compressive strain.

Double Surface-Subsurface Oxygen Vacancies						
**Strain (%)**	**1-5**	**1-7**	**3-8**	**7-4**	**2-6**	**2-5**
**+5**	2.28	1.78	2.31	2.31	2.31	1.78
**+4**	2.47	1.97	2.49	2.50	2.49	2.08

**+3**	2.62	2.25	2.67	2.66	2.66	2.17
**+2**	2.83	2.42	2.70	2.85	2.83	2.33
**+1**	2.99	2.51	3.00	2.77	3.04	2.51
**0**	3.30	2.72	3.35	3.20	3.35	2.74
**-1**	3.44	2.94	3.44	3.44	3.44	2.85
**-2**	3.54	2.86	3.54	3.15	3.54	2.86
**-3**	3.60	3.12	3.61	3.60	2.59	3.03
**-4**	3.06	3.18	3.67	3.67	3.59	3.14
**-5**	2.64	3.22	3.70	3.70	2.84	3.33

Table S9: Total energies in eV of Ce-La-O (111) with single surface oxygen vacancies at locations 1,2,3 and 4 under tensile and compressive strain.

**Single Surface Oxygen Vacancies**				
**Strain (%)**	**1**	**2**	**3**	**4**
**+5**	0.49	0.57	0.27	0.28
**+4**	0.66	0.43	0.41	0.66
**+3**	0.81	0.79	0.56	0.79
**+2**	0.94	0.94	0.73	0.94
**+1**	1.11	1.09	0.82	0.91
**0**	1.25	1.30	1.09	1.25
**-1**	1.37	1.51	1.19	1.54
**-2**	1.70	1.68	1.38	1.86
**-3**	1.97	1.83	1.58	1.84
**-4**	2.00	2.00	1.81	1.99
**-5**	2.18	2.17	2.10	2.19

Table S10: Total energies in eV of Ce-La-O (111) with single sub-surface oxygen vacancies at locations 5, 6, 7 and 8 under tensile and compressive strain.

**Single Subsurface Oxygen Vacancies**				
**Strain (%)**	**5**	**6**	**7**	**8**
**+5**	0.25	0.65	0.25	0.20
**+4**	0.77	0.34	0.82	0.29
**+3**	0.43	0.43	0.44	0.34
**+2**	0.53	0.83	0.53	0.49
**+1**	1.04	0.63	0.80	0.62
**0**	0.89	1.02	1.11	0.52
**-1**	0.81	1.21	1.21	0.89
**-2**	1.04	1.03	0.90	0.72
**-3**	1.09	1.26	1.09	0.83
**-4**	1.15	1.15	1.15	0.97
**-5**	1.19	1.19	1.19	1.10

Table S11: Total energies in eV of Ce-La-O (111) with double surface oxygen vacancies at locations 1-2 and 3-4 under tensile and compressive strain.

Double Surface Oxygen Vacancies		
**Strain (%)**	**1-2**	**3-4**
**+5**	1.32	1.33
**+4**	1.46	1.54
**+3**	1.60	1.61
**+2**	1.74	1.76
**+1**	1.89	1.90
**0**	2.05	2.07
**-1**	2.11	2.17
**-2**	2.21	2.31
**-3**	2.30	2.18
**-4**	2.21	2.07
**-5**	2.50	2.11

Table S12: Total energies in eV of Ce-La-O (111) with double sub-surface oxygen vacancies at locations 6-8, 5-6, 7-8 and 5-8 under tensile and compressive strain.

Double Subsurface Oxygen Vacancies				
**Strain (%)**	**6-8**	**5-6**	**7-8**	**5-8**
**+5**	1.21	1.21	1.10	1.10
**+4**	1.28	1.28	1.21	1.30
**+3**	1.37	1.37	1.36	1.32
**+2**	1.45	1.45	1.43	1.43
**+1**	1.54	1.54	1.54	1.55
**0**	1.64	1.65	1.48	1.49
**-1**	1.66	1.66	1.56	1.56
**-2**	1.73	1.73	1.65	1.66
**-3**	1.80	1.88	1.75	1.75
**-4**	1.86	1.86	1.83	1.85
**-5**	1.90	1.89	1.90	1.90

Table S13: Total energies in eV of Ce-La-O (111) with double sub-surface and surface oxygen vacancies at locations 1-5, 1-7, 3-8, 7-4, 2-6 and 2-5 under tensile and compressive strain.

Double Surface-Subsurface Oxygen Vacancies						
**Strain (%)**	**1-5**	**1-7**	**3-8**	**7-4**	**2-6**	**2-5**
**+5**	1.89	1.46	1.76	1.88	1.88	1.35
**+4**	2.00	1.47	1.91	2.00	2.00	1.47
**+3**	2.22	1.61	2.10	2.14	2.14	1.69
**+2**	2.24	1.74	1.70	2.24	2.24	1.81
**+1**	1.56	1.87	2.38	2.36	2.46	1.87

**0**	2.65	2.01	2.12	2.44	2.60	2.03
**-1**	1.64	2.11	1.63	2.51	2.39	2.11
**-2**	2.50	2.24	2.44	2.58	2.58	2.22
**-3**	2.82	2.40	1.87	2.82	2.65	2.32
**-4**	2.84	2.32	2.81	2.87	1.83	2.40
**-5**	2.85	2.31	2.01	2.88	2.88	2.46

Table S14: Total energies in eV of Ce-La-Cu-O (111) with single surface oxygen vacancies at locations 1,2,3 and 4 under tensile and compressive strain.

	**Single Surface Oxygen Vacancies**			
**Strain (%)**	**1**	**2**	**3**	**4**
**+5**	-1.07	-1.07	-1.07	0.45
**+4**	-1.03	-1.03	0.33	0.57
**+3**	-1.01	-1.01	-1.02	0.70
**+2**	-1.05	-1.05	0.22	0.80
**+1**	-1.47	-1.47	-0.17	0.52
**0**	-1.62	-1.62	-0.82	0.56
**-1**	-1.62	-1.62	-1.10	-1.62
**-2**	-1.64	-1.64	-1.09	-1.64
**-3**	-1.56	-1.56	-0.86	-1.56
**-4**	-1.57	-1.57	-1.00	-1.57
**-5**	-1.58	-1.58	-0.80	-1.58

Table S15: Total energies in eV of Ce-La-Cu-O (111) with single sub-surface oxygen vacancies at locations 5, 6, 7 and 8 under tensile and compressive strain.

	**Single Subsurface Oxygen Vacancies**			
**Strain (%)**	**5**	**6**	**7**	**8**
**+5**	0.69	0.69	-0.99	0.22
**+4**	-1.04	-1.04	-0.93	-0.04
**+3**	-1.02	-1.02	-0.75	-0.19
**+2**	-1.04	-0.07	0.35	-0.39
**+1**	-0.58	-0.58	-1.10	-0.98
**0**	-0.87	-0.87	-0.31	-1.32
**-1**	-1.09	-1.09	-0.53	-1.62
**-2**	-1.06	-1.06	-0.41	-1.64
**-3**	-0.97	-0.97	-1.01	-1.56
**-4**	-0.97	-0.97	-1.00	-1.57
**-5**	-1.02	-1.01	-0.98	-1.58

Table S16: Total energies in eV of Ce-La-Cu-O (111) with double surface oxygen vacancies at locations 1-2 and 3-4 under tensile and compressive strain.

Double Surface Oxygen Vacancies		
**Strain (%)**	**1-2**	**3-4**
**+5**	-0.01	0.50
**+4**	0.25	0.85
**+3**	0.16	0.66
**+2**	0.23	0.72
**+1**	-0.14	0.067
**0**	0.03	0.18
**-1**	-0.25	0.03
**-2**	-0.03	0.11
**-3**	0.03	0.54
**-4**	0.04	0.54
**-5**	0.02	0.52

Table S17: Total energies in eV of Ce-La-Cu-O (111) with double sub-surface oxygen vacancies at locations 6-8, 5-6, 7-8 and 5-8 under tensile and compressive strain.

Double Subsurface Oxygen Vacancies				
**Strain (%)**	**6-8**	**5-6**	**7-8**	**5-8**
**+5**	0.35	0.76	0.16	0.22
**+4**	0.40	0.33	0.61	0.48
**+3**	0.31	0.34	0.63	0.58
**+2**	0.33	0.31	0.63	0.49
**+1**	0.45	0.25	0.24	0.20
**0**	0.39	0.07	0.16	0.02
**-1**	0.24	-0.06	0.09	-0.13
**-2**	0.33	-0.07	0.17	0.008
**-3**	0.39	0.05	0.34	0.08
**-4**	0.43	0.04	0.32	0.06
**-5**	0.46	0.003	0.17	0.05

Table S18: Total energies in eV of Ce-La-Cu-O (111) with double sub-surface and surface oxygen vacancies at locations 1-5, 1-7, 3-8, 7-4, 2-6 and 2-5 under tensile and compressive strain.

Double Surface-Subsurface Oxygen Vacancies						
**Strain (%)**	**1-5**	**1-7**	**3-8**	**7-4**	**2-6**	**2-5**
**+5**	-0.02	0.17	-0.02	0.91	-0.02	0.35
**+4**	-0.02	0.18	-0.004	1.10	0.005	0.37
**+3**	0.02	0.19	0.52	0.66	0.27	0.37

**+2**	0.03	0.18	0.02	0.66	0.02	0.18
**+1**	-0.16	-0.02	0.19	1.21	-0.16	-0.02
**0**	-0.22	-0.10	0.11	0.01	-0.21	-0.10
**-1**	-0.26	-0.16	0.05	0.03	-0.26	-0.16
**-2**	-0.09	0.005	-0.16	0.37	0.55	-0.09
**-3**	0.10	0.06	0.11	0.43	0.39	0.09
**-4**	0.07	0.07	0.24	0.44	0.07	0.08
**-5**	0.05	0.08	0.28	0.42	0.04	-0.03

Table S19. Geometric characteristics of the adsorbed CO_2 species on CeO_2 (111)

	**C-O_{surf} length (Å)**	**O_{surf}-C-O right side angle (deg)**	**O-C-O left side angle (deg)**
**-5%**	No Bonding with the surface	No Bonding with the surface	No Bonding with the surface
**0%**	1.38	114.46	114.47
**5%**	1.35	116.27	116.33

Table S20. Geometric characteristics of the adsorbed CO_2 species on Ce-La-O (111)

	**C-O_{surf} length (Å)**	**O_{surf}-C-O right side angle (deg)**	**O_{surf}-C-O left side angle (deg)**
**-5%**	1.35	119.48	116.83
**0%**	1.34	116.19	117.36
**5%**	1.28	119.93	120.02

Table S21. Geometric characteristics of the adsorbed CO_2 species on Ce-La-Cu-O (111)

	**C-O_{surf} length (Å)**	**O_{surf}-C-O right side angle (deg)**	**O_{surf}-C-O left side angle (deg)**
**-5%**	1.32	116.86	117.57
**0%**	1.30	119.14	120.80
**5%**	1.29	118.87	121.24

Table S22. Structural and textural characteristics of 5 wt% Ni supported on Ce-La-10Cu-O carriers.

5Ni/Ce-La-10Cu-O		
**Support**	**D_{Ni} (%)**	**d_{ni} (nm)**
**Pristine**	4.1[a]	23.5
**WBM**	5.2[a]	18.6
**DBM**	4.3[a]	22.5

[a] Based on H_2 chemisorption followed by TPD.

Table S23. Total amount of oxygen removed from the solids during H_2-TPR.

**Catalyst 5wt % Ni/Ce-La-10Cu-O**		**^{16}O Total (mmol g^{-1})**	**Support alone (mmol g^{-1})**
**La**	**Pristine**	2.14	1.29
	**WBM**	1.15	0.3
	**DBM**	2.24	1.39

References

(1) Alketbi, M.; Polychronopoulou, K.; Abi Jaoude, M.; Vasiliades, M. A.; Sebastian, V.; Hinder, S. J.; Baker, M. A.; Zedan, A. F.; Efstathiou, A. M. Cu-Ce-La-Ox as efficient low-temperature CO oxidation catalysts: Effect of Cu content, Appl. Surf. Sci. 2019, 505, 144474.

(2) Suranarayana, C. and Norton, M.G. (1998) X-Ray Diffraction: A Practical Approach. Springer, New York.

(3) Lercher, J.A.; Bitter, J.H.; Hally, W.; Niessen, W.; Seshan, K. Design of stable catalysts for methane-carbon dioxide reforming, *S. Surf. Sc. Catal.* **1996**, 101, 463.

(4) Zhang, Z.; Verykios, X.; A stable and active nickel-based catalyst for carbon dioxide reforming of methane to synthesis gas," *J. Chem. Soc. Chem. Commun.* **1995**, 71.

(5) Shen, P.; Yin, S.; Li, Z.; Chen, C. Preparation and performance of nanosized tungsten carbides for electrocatalysis, *Electrochim. Acta* **2010**, 55, 7969.

(6) Cao, D.; Zeng, F.L.; Zhao, Z.J.; Cai, W.J.; Li, Y.; Yu, H.; Zhang, S.Y.; Qu, F.Z.; Cu based catalysts for syngas production from ethanol dry reforming: effect of oxide supports, *Fuel* **2018**, 219, 406.

(7) Leitenburg, Cd.; Trovarelli, A.; Llorca, J.; Cavani, F.; Bini, G. The effect of doping CeO_2 with zirconium in the oxidation of isobutane, *Appl. Catal. A: Gen.* **1996**,139,161.

(8) Pfau, A.; Schierbaum, K.D. The electronic structure of stoichiometric and reduced CeO2 surfaces: an XPS UPS and HREELS study, *Surf. Sci.* **1994**, 321, 71.

(9) Reddy, B.M.; Katta, L.; Thrimurthulu, G. Novel nanocrystalline Ce1−xLaxO2 (x=0.2) solid solutions: structural characteristics and catalytic performance, *Chem. Mater.* **2010**, 22, 467.

(10) Zhu, H., Pd/CeO_2-TiO_2 catalyst for CO oxidation at low temperature: a TPR study with H_2 and CO as reducing agents, *J. Catal.* **2004**, 225, 267.

(11) Fu, Q.; Weber, A.; Flytzani-Stephanopoulos, M. *Catal. Lett.* **2001**, 77, 87.

(12) Li, L.; Zhan, Y.; Zheng, Q.; Zheng, Y.; Chen, C.; She, Y.; Lin, X.; Wei, K. Water–gas shift reaction over CuO/CeO2 catalysts: effect of the thermal stability and oxygen vacancies of CeO2 supports previously prepared by different methods, *Catal. Lett.* **2009**, 130, 532.

(13) Yang, Y.; Zhang, S.; Wang, S.; Zhang, K.; Wang, H.; Huang, J.; Deng, S.; Wang, B.; Wang, Y.; Yu, G. Ball milling synthesized MnOx as highly active catalyst for gaseous POPs removal: significance of mechanochemically induced oxygen vacancies, Environ Sci Technol. **2015**, 49(7), 4473.
